# Supplementary material for: Green and Effective Preparation of α-Hydroxyphosphonates by Ecocatalysis
Source: Molecules. 2022 May 11;27(10):3075. doi: 10.3390/molecules27103075 (PMC9146293; doi:10.3390/molecules27103075)
Supplement: Supplementary file 1 [file molecules-27-03075-s001.zip › molecules-1705289-supplementary.pdf]

The Supporting Information

Green and Effective Preparation of α-Hydroxyphosphonates by Ecocatalysis

Pola Cybulska [1], Yves-Marie Legrand [2], Alicja Babst-Kostecka [3], Sébastien Diliberto [4], Anna Leśniewicz [5], Erwan Oliviero [6], Valérie Bert [7], Clotilde Boulanger [4], Claude Grison [2],* and Tomasz K. Olszewski [1],*

1 Department of Physical and Quantum Chemistry, Faculty of Chemistry, Wrocław University of Science and Technology, Wybrzeże Wyspiańskiego 27, 50-370 Wrocław, Poland

2 Bio-inspired Chemistry and Ecological Innovations (ChimEco), UMR 5021 CNRS, University of Montpellier, Cap Delta, 1682 rue de la Valsière, 34790 Grabels, France

3 Department of Environmental Science, The University of Arizona, Tucson, AZ 85721, USA.

4 Institut Jean Lamour, UMR 7198 CNRS, University of Lorraine, 57000 Metz, France

5 Analytical Chemistry and Chemical Metallurgy Division, Faculty of Chemistry, Wrocław University of Science and Technology, Wybrzeże Wyspiańskiego 27, 50-370 Wrocław, Poland

6 ICGM, University of Montpellier, CNRS, Montpellier, France

7 Clean Technologies and Circular Economy Unit, SIT Department, INERIS, Parc Technologique Alata BP 2, 60550, Verneuil en Halatte, France

*Correspondence: claude.grison@cnrs.fr (CG); tomasz.olszewski@pwr.edu.pl (TKO)

Table of contents

Figure SI 1. XRPD diffraction pattern 2
Figure SI 2. HRTEM images................ 3
Characterisation of products................ 4
Optimization of the reaction conditions................ 8
Table SI1. Optimization of the reaction conditions................ 9
Table SI2. Recovery and reuse tests of the Eco-MgZnOx catalysts..10
NMR Spectra of the obtained products..11
Theoretical calculations..51

Figure SI 1. XRPD diffraction pattern of a) Eco-MgZnOx-P and b) Eco-MgZnOx-F

a)

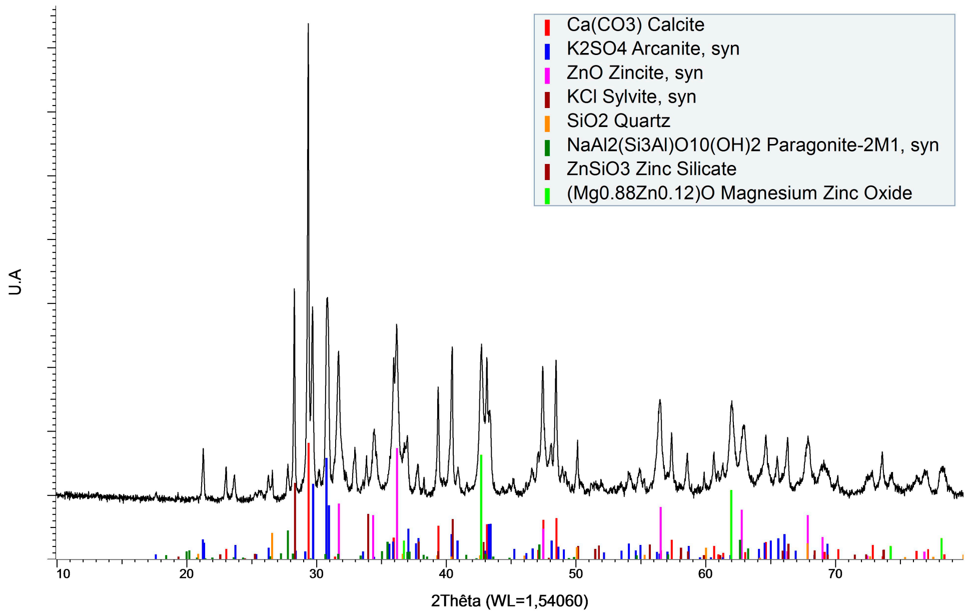

b)

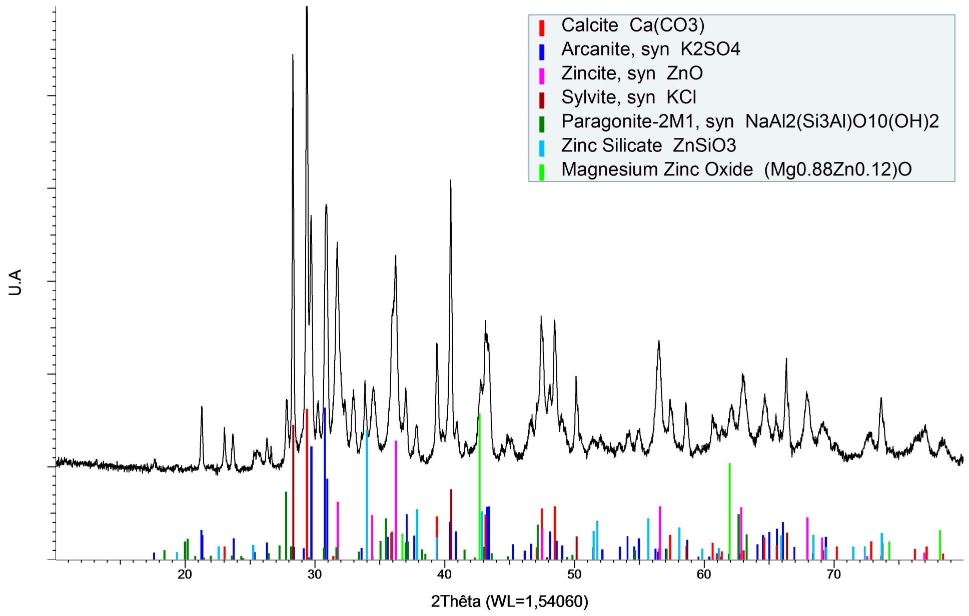

Figure SI 2. HRTEM images of (a) Eco-MgZnOx-P and (b) Eco-MgZnOx-F embedded in resin

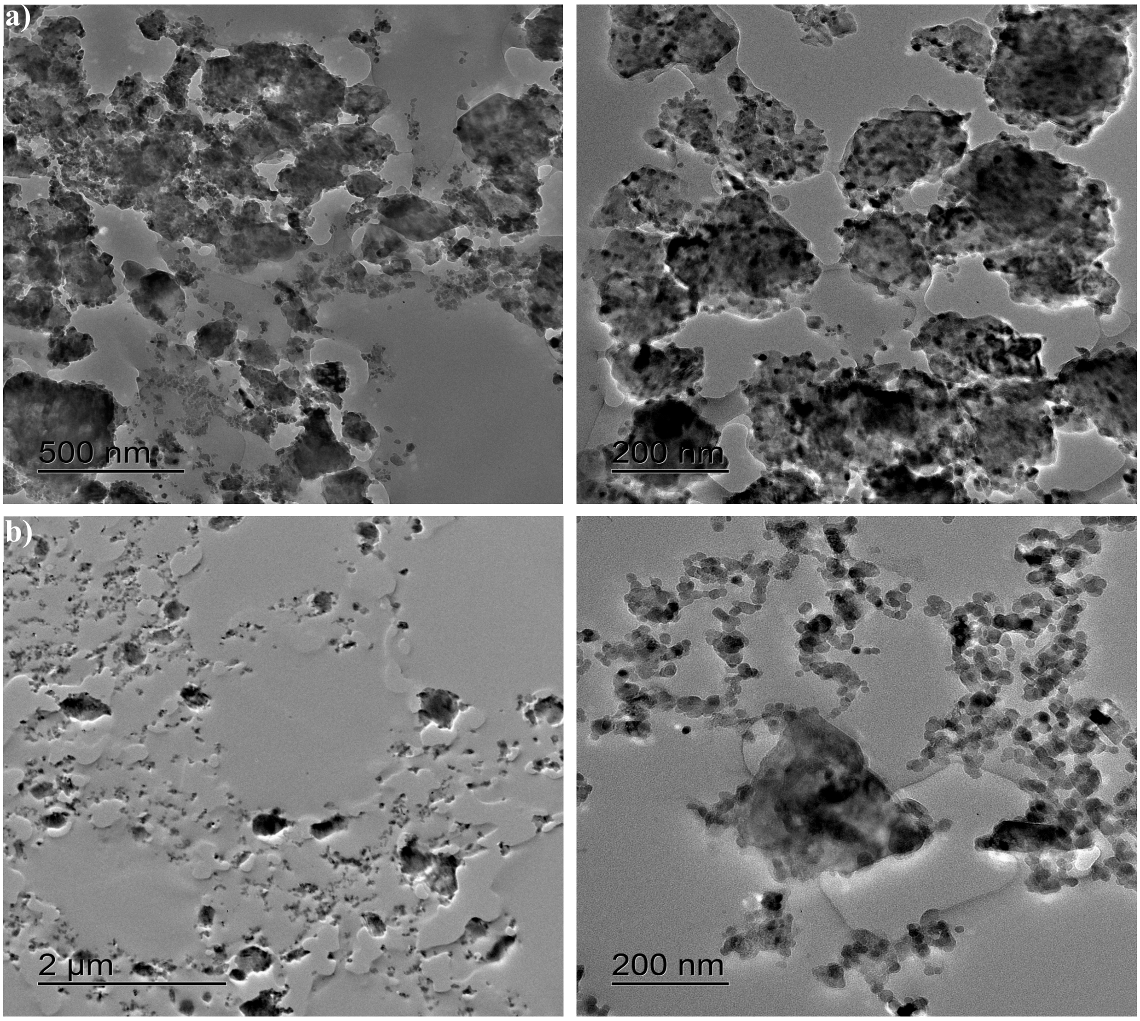

Characterisation of products

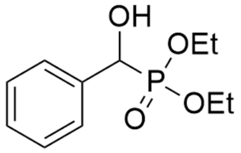

Diethyl (1-hydroxyphenylmethyl)phosphonate **(3a)**

White solid; yield = 82% (148 mg); mp. 83-85 °C; ^{1}H NMR (400 MHz, $CDCl_3$): δ 7.48-7.46 (m, 2H), 7.36-7.28 (m, 3H), 5.00 (d, *J*=11.2 Hz, 1H), 4.07-3.92 (m, 4H), 1.25 (t, *J*=7.0 Hz, 3H), 1.20 (t, *J*=7.0 Hz, 3H). ^{13}C NMR (100 MHz, $CDCl_3$): δ 136.8, 128.1, 127.3, 127.2, 71.6 (d, *J*=158.3 Hz), 63.5 (d *J*=7.4 Hz), 63.1 (d, *J*=7.4 Hz), 16.4 (d, *J*=2.9 Hz), 16.3 (d, *J*=2.3 Hz). ^{31}P NMR (243 MHz, $CDCl_3$): δ 22.1 (s). HRMS (ESI-TOF) calcd. for $C_{11}H_{17}O_4PNa$ $[M+Na]^+$ m/z: 267.0762 found: 267.0757.

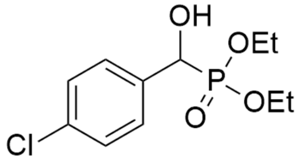

Diethyl (4-chloro-1-hydroxybenzyl)phosphonate **(3b)**

White solid; yield = 80% (214 mg); mp. 55-58 °C; ^{1}H NMR (400 MHz, $CDCl_3$): δ 7.43-7.38 (m, 2H), 7.32 (d, *J*=8.8 Hz, 2H), 5.00 (d, *J*=10.8 Hz, 1H), 4.11-3.96 (m, 4H), 1.29-1.20 (m, 6H). ^{13}C NMR (100 MHz, $CDCl_3$): δ 135.9, 133.2 (d, *J*=3.4 Hz), 128.5 (d, *J*=2.9 Hz), 128.4, 70.1 (d, *J*=159.5 Hz), 63.8 (d, *J*=6.8 Hz), 63.4 (d, *J*=7.4 Hz), 16.5 (d, *J*=5.2 Hz). ^{31}P NMR (243 MHz, $CDCl_3$): δ 22.1 (s); HRMS (ESI-TOF) calcd. for $C_{11}H_{16}ClO_4PNa$ $[M+Na]^+$ m/z: 301.0372 found: 301.0375.

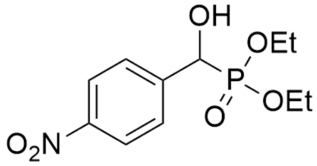

Diethyl (4-nitro-1-hydroxybenzyl)phosphonate (**3c**)

Yellow solid; yield = 72% (200mg); mp. 86-88°C; ^{1}H NMR (400 MHz, $CDCl_3$): δ 8.21 (d, *J*= 8.4 Hz, 2H), 7.66-7.63 (m, 2H), 5.18 (d, *J*=12.0 Hz, 1H), 4.99 (bs, 1H) 4.16-4.02 (m, 4H), 1.30-1.22 (m, 6H); ^{13}C NMR (100 MHz, $CDCl_3$): δ 147.6 (d, *J*= 3.5 Hz), 144.2, 127.8 (d, *J*=4.5 Hz), 123.4 (d, *J*=2.2 Hz), 70.1 (d, *J*= 157.7 Hz), 64.2 (d, *J*= 6.8 Hz), 63.5 (d, *J*= 8.0 Hz), 16.5 (m); ^{31}P NMR (243 MHz, $CDCl_3$): δ 20.4 (s); HRMS (ESI-TOF) calcd. for $C_{11}H_{16}NO_6PNa$ $[M+Na]^+$ m/z: 312.0613 found: 312.0608.

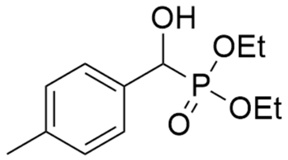

Diethyl (4-methyl-1-hydroxybenzyl)phosphonate (**3d**)

White solid; yield = 82% (203mg); mp. 94-97°C; ^{1}H NMR (400 MHz, $CDCl_3$): δ 7.37-7.33 (m, 2H), 7.15 (d, *J* = 8.0 Hz, 2H), 4.96 (d, *J* = 10.4 Hz, 1H), 4.08-3.96 (m, 4H), 2.33 (s, 3H), 1.26 (t, *J* = 7.2 Hz, 3H), 1.20 (t, *J* = 7.2 Hz, 3H); ^{13}C NMR (100 MHz, $CDCl_3$): δ 137.9, 133.6, 129.1 (d, *J*=1.7 Hz), 127.1 (d, *J*=6.3 Hz), 70.3 (d, *J*= 158.9 Hz), 63.4 (d, *J*= 7.4 Hz), 63.1 (d, J=7.5 Hz), 21.3, 16.4 (m); ^{31}P NMR (243 MHz, $CDCl_3$): δ 22.2 (s); HRMS (ESI-TOF) calcd. for $C_{12}H_{19}O_4PNa$ $[M+Na]^+$ m/z: 281.0919 found:281.0914.

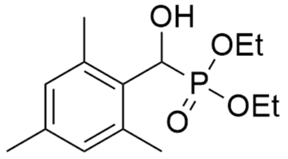

Diethyl (2,4,6-trimethyl-1-hydroxybenzyl)phosphonate **(3e)**

White solid; yield = 75% (201mg); mp. 90-92°C; ^{1}H NMR (400 MHz, $CDCl_3$): δ 6.81 (s, 2H), 5.45 (d, *J*=15.6 Hz, 1H), 4.16-4.03 (m, 2H), 4.02-3.90 (m, 1H), 3.83-3.72 (m, 1H), 2.45 (s, 6H), 2.23 (m, 3H), 1.29 (t, *J*=7.0 Hz, 3H), 1.12 (t, *J*=6.8 Hz, 3H); ^{13}C NMR (100 MHz, $CDCl_3$): δ 135.2, 137.6 (d, *J*=3.4 Hz), 129.7, 68.7 (d, *J*=158,3 Hz), 63.1 (d, *J*=6.8 Hz), 62.7 (d, *J*=7.5 Hz), 21.1, 20.9, 16.5 (d, *J*=5.8 Hz), 16.3 (d, *J*=5.7 Hz); ^{31}P NMR (243 MHz, $CDCl_3$): δ 24.3 (s); HRMS (ESI-TOF) calcd. for $C_{14}H_{23}O_4PNa$ [M+Na]+ m/z: 309.1232 found: 309.1240.

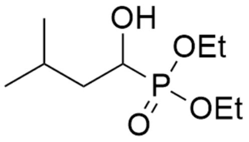

Diethyl 1-hydroxy-3-methyl-butylphosphonate **(3f)**

Yellow oil; yield = 84% (181mg); ^{1}H NMR (400 MHz, $CDCl_3$): δ 4.17-4.06 (m, 4H), 3.93-3.87 (m, 1H), 1.94-1.84 (m, 1H), 1.71-1601 (m, 1H), 1.44-1.35 (m, 1H), 1.30-1.28 (m, 6H), 0.91 (d, *J*=6.8 Hz, 3H) 0,86 (d, *J*=6.6 Hz, 3H); ^{13}C NMR (100 MHz, $CDCl_3$): δ 66.0 (d, *J*=161.0 Hz), 62.6, 40.0, 24.1 (d, *J*=13.7 Hz), 23.5, 21.1, 16.5 (d, *J*=4.6 Hz); ^{31}P NMR (243 MHz, $CDCl_3$): δ 24.3 (s); HRMS (ESI-TOF) calcd. for $C_9H_{21}O_4PNa$ $[M+Na]^+$ m/z: 247.1075 found: 247.1070.

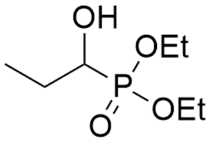

Diethyl 1-hydroxypropylphosphonate (**3g**)

Yellow oil; yield = 83% (156mg); ^{1}H NMR (400 MHz, $CDCl_3$): δ 4.16-4.07 (m, 4H), 3.78-3.70 (m, 1H), 1.83-1.60 (m, 2H), 1.31-1.6 (m, 6H), 1.04-1.00 (m, 3H) ; ^{13}C NMR (100 MHz, $CDCl_3$): δ 69.2 (d, J=159.4 Hz), 62.6 (m), 24.7, 16.5 (d, *J*=4.5 Hz), 10.5 (d, *J*=13.1 Hz); ^{31}P NMR (243 MHz, $CDCl_3$): δ 26.0 (s); HRMS (ESI-TOF) calcd. for $C_7H_{17}O_4PNa$ $[M+Na]^+$ m/z: 219.0762 found: 219.0760.

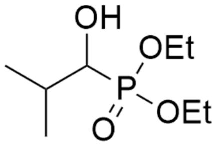

Diethyl 1-hydroxy-2-methyl-propylphosphonate **(3h)**

Yellow oil; yield = 81% (164mg); ^{1}H NMR (400 MHz, $CDCl_3$): δ 4.20-4.10 (m, 4H), 3.65-3.60 (m, 1H), 3.0 (bs, 1H), 2.11-2.00 (m, 1H), 1.33-1.21 (m, 6H), 1.02-1.05 (m, 6H); ^{13}C NMR (100 MHz, $CDCl_3$): δ 72.9 (d, *J*=156 Hz), 62.50 (d, *J*=7.0 Hz), 30.2 (d, *J*=2.3 Hz), 19.9 (d, *J*=9.7 Hz) 17.8 (d, *J*=7.4 Hz), 16.5 (d, *J*=5.7 Hz); ^{31}P NMR (243 MHz, $CDCl_3$): δ 26.0 (s); HRMS (ESI-TOF) calcd. for $C_8H_{19}O_4PNa$ $[M+Na]^+$ m/z: 233.0919 found: 233.0922.

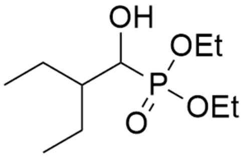

Diethyl 2-ethyl-1-hydroxybutylphosphonate (**3i**)

Yellow oil; yield = 84% (192mg); ^{1}H NMR (400 MHz, $CDCl_3$): δ 4.17-4.07 (m, 4H), 3.89-3.85 (m, 1H), 1.75-1.65 (m, 1H), 1.61-1.32 (m, 4H), 1.29 (t, *J*=7.0 Hz, 6H), 0.88-0.84 (m, 6H); ^{13}C NMR (100 MHz, $CDCl_3$): δ 69.4 (d, *J*=156.6 Hz), 62.5 (d, *J*=7.5Hz), 42.9 (d, *J*=2.8 Hz), 21.9 (d, *J*=9.7 Hz), 21.1 (d, *J*=5.7 Hz), 16.5 (d, *J*=5.7 Hz), 11.4 (d, *J*=16.6 Hz); ^{31}P NMR (243 MHz, $CDCl_3$): δ 26.6 (s); HRMS (ESI-TOF) calcd. for $C_{10}H_{23}O_4PNa$ $[M+Na]^+$ m/z: 261.1232 found: 261.1223.

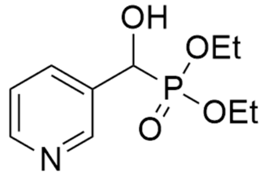

Diethyl 1-hydroxy(pyridine-3-yl)methylphosphonate **(3j)**

White solid; yield = 87% (205mg); mp. 125-127°C; ^{1}H NMR (400 MHz, $CDCl_3$): δ 8.60 (s, 1H), 8.47-8.44 (m, 1H), 7.88-7.83 (m, 1H), 7.28-7.24 (m, 1H), 5.04 (d, *J*=12.0 Hz, 1H), 4.11-3.99 (m, 4H), 1.25-1.18 (m, 6H); ^{13}C NMR (100 MHz, $CDCl_3$): δ 148.6 (d, *J*=2.3 Hz), 148.2 (d, *J*=6.2Hz), 135.4 (d, *J*=5.2Hz), 133.8, 123.4, 68.3 (d, *J*=162.9 Hz), 63.6 (d, *J*=7.4 Hz), 63.2 (d, *J*=7.4 Hz), 16.3 (m); ^{31}P NMR (243 MHz, $CDCl_3$): δ 21.1 (s); HRMS (ESI-TOF) calcd. for $C_{10}H_{16}NO_4PNa$ $[M+Na]^+$ m/z: 268.0715 found: 268.0724.

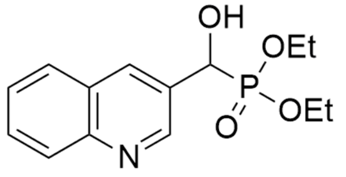

Diethyl 1-hydroxy(quinolin-3-yl)methylphosphonate **(3k)**

Beige solid; yield = 82% (232mg); mp. 76-77°C; ^{1}H NMR (400 MHz, $CDCl_3$): δ 9.01 (s, 1H), 8.46-8.38 (m, 1H), 8.22-8.12 (m, 1H), 7.86-7.80 (m, 1H), 7.78-7.70 (m, 1H), 7.61-7.57 (m, 1H), 5.36-5.29 (m, 1H), 4.17-4.06 (m, 4H), 1.29-1.20 (m, 6H); ^{13}C NMR (100 MHz, $CDCl_3$): δ 149.4 (d, *J*=4.6 Hz), 146.9, 134.9 (d, *J*=6.3 Hz), 130.9, 129.9, 128.5, 128.1, 127.8, 127.1, 68.6 (d, *J*=162.8 Hz), 63.7 (d, *J*=6.9 Hz), 63.3 (d, *J*=7.4 Hz), 16.4 (m); ^{31}P NMR (243 MHz, $CDCl_3$): δ 20.5 (s); HRMS (ESI-TOF) calcd. for $C_{14}H_{18}NO_4PNa$ $[M+Na]^+$ m/z: 318.0871 found: 318.0867.

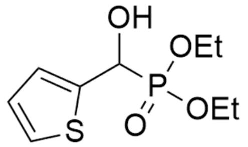

Diethyl 1-hydroxy(thiophen-2-yl)methylphosphonate **(3l)**

White solid; yield = 86% (207mg); mp. 28°C; ^{1}H NMR (400 MHz, $CDCl_3$): δ 7.40-7.38 (m, 1H), 7.29-7.26 (m, 1H), 7.19-7.16 (m, 1H), 5.08 (d, *J*=10.8 Hz, 1H), 4.14-3.99 (m, 4H), 1.26 (t, *J*=7.0 Hz, 3H), 1.20 (t, *J*=7.2 Hz, 3H); ^{13}C NMR (100 MHz, $CDCl_3$): δ 137.8, 126.8 (m), 125.7, 123.0 (d, *J*=9.2 Hz), 67.4 (d, *J*=162.3 Hz), 63.5 (d, *J*=6.8Hz) 63.2 (d, *J*=7.4Hz), 16.5 (m); ^{31}P NMR (243 MHz, $CDCl_3$): δ 21.7 (s); HRMS (ESI-TOF) calcd. for $C_9H_{15}O_4PSNa$ $[M+Na]^+$ m/z: 273.0327 found: 273.0331.

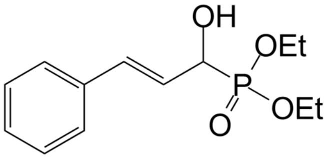

Diethyl (1-hydroxy-3-phenyl)prop-2-enyl-phosphonate (3**m)**

Pale yellow solid, yield = 90% (224mg); mp. 103-105°C; ^{1}H NMR (400 MHz, $CDCl_3$): δ 7.39-7.18 (m, 5H), 6.80-6.73 (m, 1H), 6.35-6.27 (m, 1H), 4.70-4.64 (m, 1H), 4.21-4.12 (m, 4H), 1.31-1.26 (m, 6H); ^{13}C NMR (100 MHz, $CDCl_3$): δ 136.6 (d, *J*=2.8 Hz), 132.2 (d, *J*=13.2 Hz), 128.6, 127.9, 126.7, 124.3 (d, *J*=4.0 Hz), 70.3, 69.4 (d, *J*=160.9 Hz), 63.5 (d, *J*=6.8 Hz), 63.2 (d, *J*=6.9 Hz), 16.6 (d, *J*=5.2 Hz); ^{31}P NMR (243 MHz, $CDCl_3$): δ 22.4.

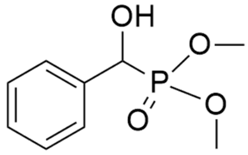

Dimethyl (1-hydroxyphenylmethyl)phosphonate (**5b**)

White solid; yield = 92% (182mg); mp. 84-86°C; ^{1}H NMR (400 MHz, $CDCl_3$): δ 7.49-7.45 (m, 2H), 7.36-7.24 (m, 3H), 5.06 (d, *J*=11.2 Hz, 1H), 3.71-3.64 (m, 6H); ^{13}C NMR (100 MHz, $CDCl_3$): δ 136.9, 128.4, 128.3, 127.1, 70.6 (d, *J*=158.8 Hz), 54.1 (d, *J*=7,4Hz), 53.7 (d, *J*=7,4Hz); ^{31}P NMR (243 MHz, $CDCl_3$): δ 24.1 (s); HRMS (ESI-TOF) calcd. for $C_9H_{13}NaO_4P$ [M+Na]+ m/z: 239.0449 found: 239.0445.

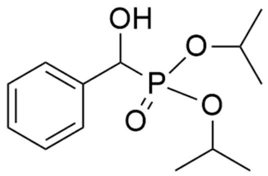

Diisopropyl (1-hydroxyphenylmethyl)phosphonate (**5c)**

White solid; yield = 62% (203mg); mp. 93-96°C; ^{1}H NMR (400 MHz, $CDCl_3$): δ 7.50-7.46 (m, 2H), 7.40-7.26 (m, 3H), 5.05 (d, *J*=10.8 Hz, 1H), 4.63-4.50 (m, 2H), 1.26-1.12 (m, 12H); ^{13}C NMR (100 MHz, $CDCl_3$): δ 136.9, 128.2, 128.0, 127.4, 71.5 (m), 24.2 (m); ^{31}P NMR (243 MHz, $CDCl_3$): δ 20.5 (s); HRMS (ESI-TOF) calcd. for $C_{13}H_{21}O_4PNa$ $[M+Na]^+$ m/z: 295.1075 found: 295.1072.

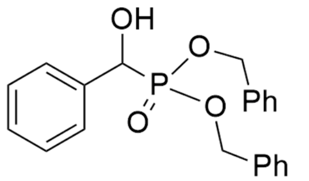

Dibenzyl (1-hydroxyphenylmethyl)phosphonate (**5d)**

Beige solid; yield = 85% (301mg); mp. 104-107°C; ^{1}H NMR (400 MHz, $CDCl_3$): δ 7.46-7.17 (m, 15H), 5.09-4.83 (m, 5H), 3.04 (bs, 1H); ^{13}C NMR (100 MHz, $CDCl_3$): δ 136.1, 128.6 (m), 127.3, 71.1 (d, *J*=157.7 Hz), 68.8 (m); ^{31}P NMR (243 MHz, $CDCl_3$): δ 22.7 (s).

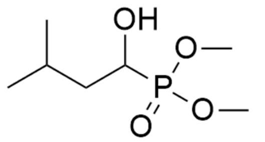

Dimethyl 1-hydroxy-3-methyl-butylphosphonate (**5e**)

Beige solid; yield = 87% (164mg); mp. 52-54°C; ^{1}H NMR (400 MHz, $CDCl_3$): δ 4.00-3.95 (m, 1H), 3.80-3.77 (m, 6H), 1.97-1.85 (m, 1H), 1.75-1.65 (m, 1H), 1.47-1.39 (m, 1H), 0.94-0.88 (m, 6H); ^{13}C NMR (100 MHz, $CDCl_3$): δ 65.8 (d, *J*=160.6 Hz), 53.3-53.5(m), 39.9, 24.1 (d, *J*=14.3 Hz), 23.5, 21.1; ^{31}P NMR (243 MHz, $CDCl_3$): δ 28.6 (s); HRMS (ESI-TOF) calcd. for $C_7H_{17}O_4PNa$ $[M+Na]^+$ m/z: 219.0762 found: 219.0760.

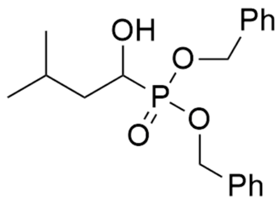

Dibenzyl 1-hydroxy-3-methyl-butylphosphonate (**5f)**

White solid; yield = 80% (168mg); mp. 72-74°C; ^{1}H NMR (400 MHz, $CDCl_3$): δ 7.35-7.30 (m, 10H), 5.13-5.00 (m, 4H), 4.01-3.95 (m, 1H), 1.94-1.85 (m, 1H), 1.77-1.65 (m, 1H), 1.48-1.39 (m, 1H), 0.90 (d, *J*=6.8 Hz, 3H), 0.83 (d, *J*=6.4 Hz, 3H); ^{13}C NMR (100 MHz, $CDCl_3$): δ 136.4 (m), 128.7, 128.5, 128.1 (m), 68.1 (t, *J*=6.8 Hz), 66.5 (d, *J*=158.3 Hz), 39.8, 24.2, 24.1, 23.5, 21.1; ^{31}P NMR (243 MHz, $CDCl_3$): δ 27.3 (s); HRMS (ESI-TOF) calcd. for $C_{19}H_{25}O_4PNa$ [M+Na]+ m/z: 371.1388 found: 371.1393.

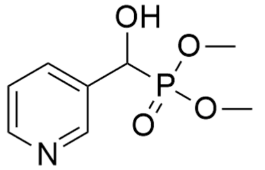

Dimethyl 1-hydroxy(pyridine-3-yl)methylphosphonate **(5g)**

Orange solid; yield = 90% (188mg); mp. 123-125°C; ^{1}H NMR (400 MHz, $CDCl_3$): δ 8.61 (s, 1H), 8.49-8.46 (m, 1H), 7.90-7.85 (m, 1H), 7.31-7.27 (m, 1H), 5.10 (d, *J*=12.4 Hz, 1H), 3.72-3.70 (m, 6H); ^{13}C NMR (100 MHz, $CDCl_3$): δ 148.8 (d, *J*=2.9 Hz), 148.1 (d, *J*=6.8 Hz), 135.5 (d, *J*=5.1 Hz), 133.4, 123.6, 68.2 (d, *J*=162.9 Hz), 54.1 (d, *J*=6.8 Hz), 53.8 (d, *J*=7.5 Hz); ^{31}P NMR (243 MHz, $CDCl_3$): δ 23.2(s); HRMS (ESI-TOF) calcd. for $C_8H_{12}O_4PNa$ $[M+Na]^+$ m/z: 240.0402 found: 240.0392.

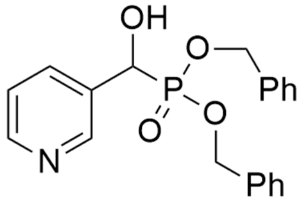

Dibenzyl 1-hydroxy(pyridine-3-yl)methylphosphonate (**5h**)

Dark brown solid; yield = 83% (294mg); mp. 115-118°C; ^{1}H NMR (400 MHz, $CDCl_3$): δ 8.11 (s, 1H), 8.02 (d, *J*=8.4 Hz, 1H), 7.64-7.40 (m, 2H), 7.20-7.10 (m, 10H), 5.31 (d, *J*=12.0 Hz, 1H), 5.09-4.88 (m, 4H); ^{13}C NMR (100 MHz, $CDCl_3$): δ 149.7 (d, *J*=4.6 Hz), 147.3, 136.0 (d, *J*=5.7 Hz), 134.9 (d, *J*=6.3 Hz), 130.6, 127.0 (m), 68.8 (m); ^{31}P NMR (243 MHz, $CDCl_3$): δ 22.2 (s); HRMS (ESI-TOF) calcd. for $C_{20}H_{20}O_4PNNa$ $[M+Na]^+$ m/z: 392.1028 found: 392.1020.

Optimization of the reaction conditions

Initially, reaction was performed under solventless conditions, and this turned out to be the best choice. Reaction proceeded already at room temperature but with poor to moderate conversions depending on the reaction time (Table SI1 entry 1 and 2). When heating was applied conversions were greatly improved and reaction time was limited to only 3h (Table SI1 entry 3 and 4). Further, the amount of plant-based catalyst could be decreased from initial Mg (28.1 mol%), Ca (54.5 mol%), Zn (22.8 mol%) for Eco-ZnOx-P and Mg (14.2 mol%), Ca (45.5 mol%), Zn (19.7 mol%) for Eco-ZnOx-F (Table SI1 entries 1-4) to optimal amount Mg (7.0 mol%), Ca (13.6 mol%), Zn (5.7 mol%) for Eco-ZnOx-P and Mg (3.5 mol%), Ca (11.4 mol%), Zn (4.9 mol%) for Eco-ZnOx-F (Table SI1, entries 6, 9-16). Lower amounts of Mg, Ca and Zn resulted in significant decrease in the reaction conversion (Table SI1, entry 7).

Thus, Eco-ZnOx-P [Mg (7.0 mol%), Ca (13.6 mol%), Zn (5.7 mol%)] and Eco-ZnOx-F [Mg (3.5 mol%), Ca (11.4 mol%), Zn (4.9 mol%)], 50 °C, 3h and solventless conditions was set as optimized parameters for the hydrophosphonylation of model substrates.

When diethyl *H*-phosphonate (**2**, R^2=Et) was changed to dibenzyl *H*-phosphonate (**2**, R^2=Bn) under the optimized reaction conditions high conversions were observed. However, changing the benzaldehyde to isovaleraldehyde (**1**, $R^1=(CH_3)_2(CH)CH_2$) or 3-pyridinecarboxaldehyde (**1**, R^1=3-pyridine) in the model reaction required increasing the reaction temperature from 50 °C to 70 °C to maintain high levels of conversion (Table SI1, entries 10-14). Furthermore, reactions without the Eco-ZnOx catalysts did not produce desired α-hydroxyphosphonate (Table SI1, entry 8). Replacing the diethyl *H*-phosphonate (**2**, R^2=Et) with triethylphosphite (R^2=Et) and application of optimized reaction conditions resulted in poor conversion of only 17% (Table SI1, entry 15). Importantly use of solvents such as toluene and ethyl acetate under optimized reaction conditions did not lead to the desired products (Table SI1, entry 16).

Table SI1. Optimization of the reaction conditions.

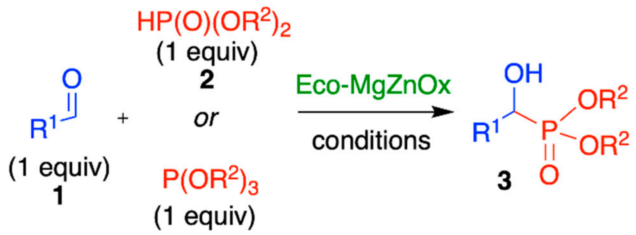

R^1 = Ph or $(CH_3)_2(CH)CH_2$ or 3-pyridine
R^2 = Et or Bn

Entry	Conditions[a]	plant-based catalyst	Conversion[b] (%)
1	R^1=Ph, R^2=Et,<br>1h, rt	Eco-ZnOx-P[d]<br>Eco-ZnOx-F[e]	28<br>22
2	R^1=Ph, R^2=Et,<br>24h, rt	Eco-ZnOx-P[d]<br>Eco-ZnOx-F[e]	86<br>80
3	R^1=Ph, R^2=Et,<br>1h, 50 °C	Eco-ZnOx-P[d]<br>Eco-ZnOx-F[e]	76<br>80
4	R^1=Ph, R^2=Et,<br>3h, 50 °C	Eco-ZnOx-P[d]<br>Eco-ZnOx-F[e]	97<br>96
5	R^1=Ph, R^2=Et,<br>3h, 50 °C	Eco-ZnOx-P[f]<br>Eco-ZnOx-F[g]	97<br>96
**6**	**R^1=Ph, R^2=Et,<br>3h, 50 °C**	Eco-ZnOx-P[h]<br>Eco-ZnOx-F[i]	97<br>96
7	R^1=Ph, R^2=Et,<br>3h, 50 °C	Eco-ZnOx-P[j]<br>Eco-ZnOx-F[k]	47<br>40
8	R^1=Ph, R^2=Et,<br>3h, 50 °C	-	0
9	R^1=Ph, R^2=Bn,<br>3h, 50 °C	Eco-ZnOx-P[h]<br>Eco-ZnOx-F[i]	99<br>86
10	R^1=$CH_2(CH)(CH_3)_2$, R^2=Et,<br>3h, 50 °C	Eco-ZnOx-P[h]<br>Eco-ZnOx-F[i]	70<br>79
11	R^1=$CH_2(CH)(CH_3)_2$, R^2=Et,<br>5h, 50 °C	Eco-ZnOx-P[h]<br>Eco-ZnOx-F[i]	79<br>89
**12**	**R^1=$CH_2(CH)(CH_3)_2$, R^2=Et,<br>3h, 70 °C**	Eco-ZnOx-P[h]<br>Eco-ZnOx-F[i]	**95<br>99**
13	R^1=3-pyridyl, R^2=Et,<br>3h, 50 °C	Eco-ZnOx-P[h]<br>Eco-ZnOx-F[i]	78<br>84
**14**	**R^1=3-pirydyl, R^2=Et,<br>3h, 70 °C**	Eco-ZnOx-P[h]<br>Eco-ZnOx-F[i]	**92<br>82**
15	R^1=Ph, R^2=Et, 3h, 50 °C<br>with $P(OEt)_3$ as nucleophile	Eco-ZnOx-P[h]<br>Eco-ZnOx-F[i]	17<br>17
16	R^1=Ph, R^2=Et, 3h, 50 °C,<br>solvent[c]	Eco-ZnOx-P[h]<br>Eco-ZnOx-F[i]	0<br>0

[a]Reaction conditions: aldehyde (0.23 mmol), *H*-phosphonate or $P(OEt)_3$ (0.23 mmol), Eco-ZnOx-P or Eco-ZnOx-F, solventless conditions. [b]Determined by ^{31}P NMR of the crude reaction mixture. [c]Toluene and ethyl acetate were used as solvents; reaction without the catalyst carried out in those solvents also did not produced the desired product. rt – room temperature. – no catalyst.[d] Mg (28.1 mol%), Ca (54.5 mol%), Zn (22.8 mol%). [e]Mg (14.2 mol%), Ca (45.5 mol%), Zn (19.7 mol%). [f]Mg (14.1 mol%), Ca (27.2 mol%), Zn (11.4 mol%). [g]Mg (7.1 mol%), Ca (22.7 mol%), Zn (9.9 mol%). [h]Mg (7.0 mol%), Ca (13.6 mol%), Zn (5.7 mol%). [i]Mg (3.5 mol%), Ca (11.4 mol%), Zn (4.9 mol%). [j]Mg (1.4 mol%), Ca (2.7 mol%), Zn (1.1 mol%). [k]Mg (0.7 mol%), Ca (2.3 mol%), Zn (1.0 mol%).

Table SI2. Recovery and reuse tests of the Eco-MgZnOx catalysts.[a]

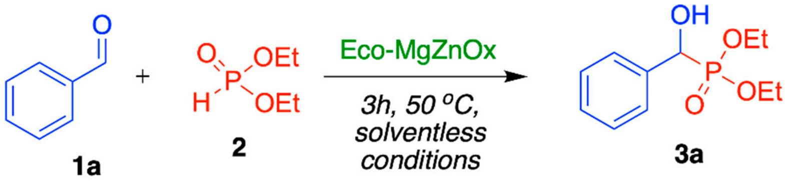

Eco-MgZnOx	Run	Conversion. (%)	Mineral composition of Eco-MgZnOx			
				Ca	Mg	Zn
**Eco-MgZnOx-P**	1	97	w%	16.21	4.32	8
			%RSD	0.47	0.63	0.07
**Eco-MgZnOx-F**	1	96	w%	14.77	1.69	7.22
			%RSD	1.2	0.53	0.41
**Eco-MgZnOx-P**	2	96	w%	15.92	3.72	6.96
			%RSD	0.74	0.2	0.43
**Eco-MgZnOx-F**	2	58	w%	14.46	1.26	5.23
			%RSD	1.07	0.38	1.62
**Eco-MgZnOx-P**	3	94	w%	13.65	2.94	5.27
			%RSD	1.01	0.43	0.94
**Eco-MgZnOx-F**	3	14	w%	15.42	0.79	3.45
			%RSD	0.24	0.3	0.91

[a]Reaction conditions: aldehyde (0.23 mmol), *H*-phosphonate (0.23 mmol), Eco-MgZnOx, 3h, 50 °C solventless. [b]Conversion determined by ^{31}P NMR of the crude reaction mixture. [c]Mineral compositions were determined by using a microwave plasma-atomic emission spectroscopy (MP-AES).

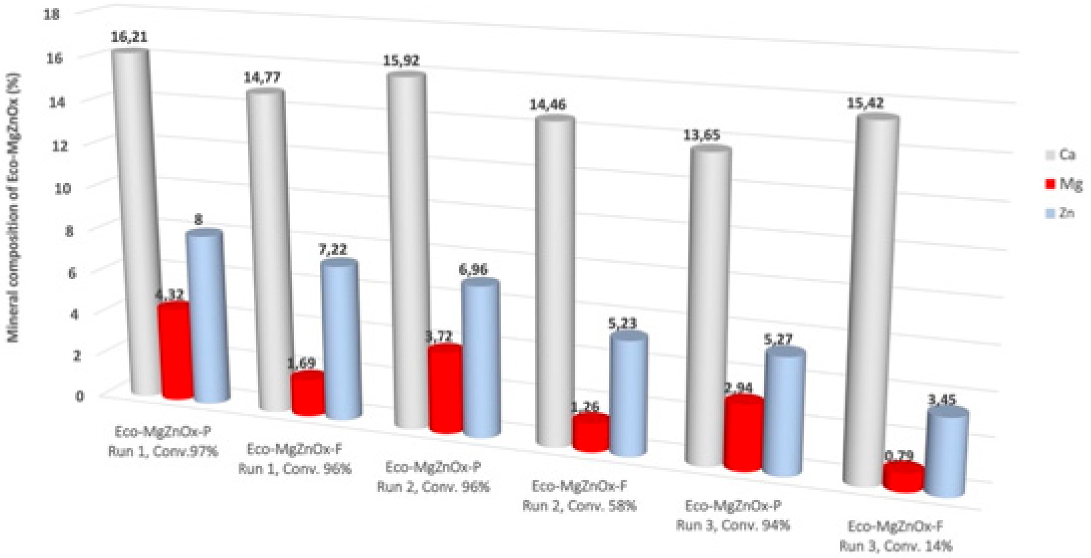

NMR Spectra of the obtained products

Diethyl (1-hydroxyphenylmethyl)phosphonate (3a)

^{1}H NMR spectrum of **3a**

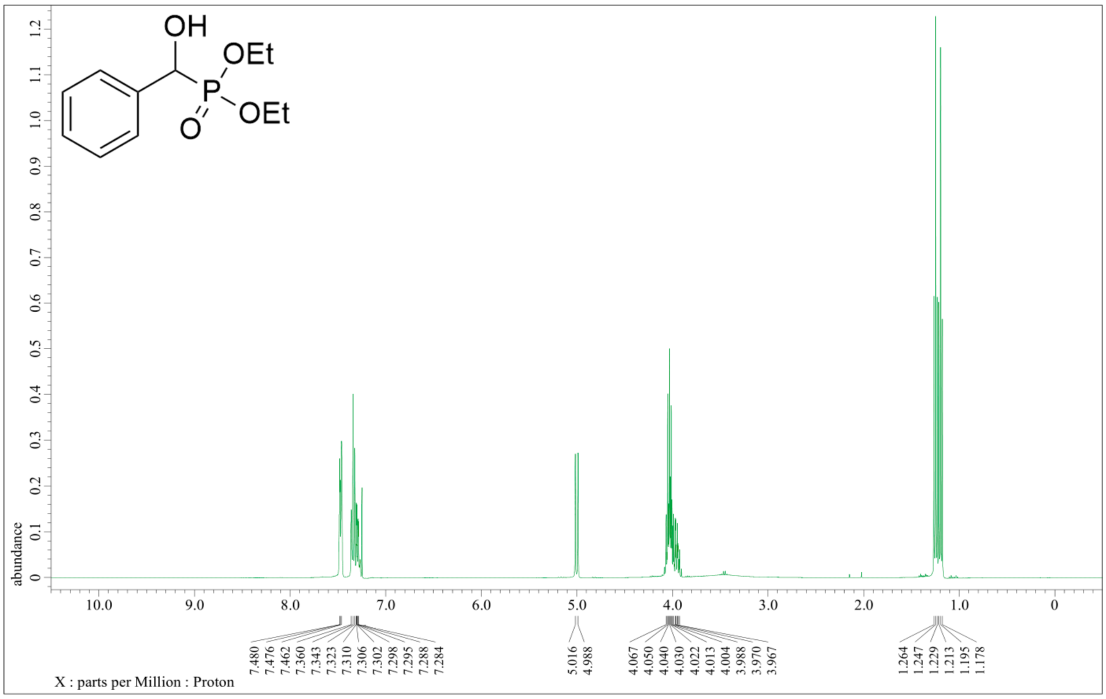

^{13}C NMR spectrum of **3a**

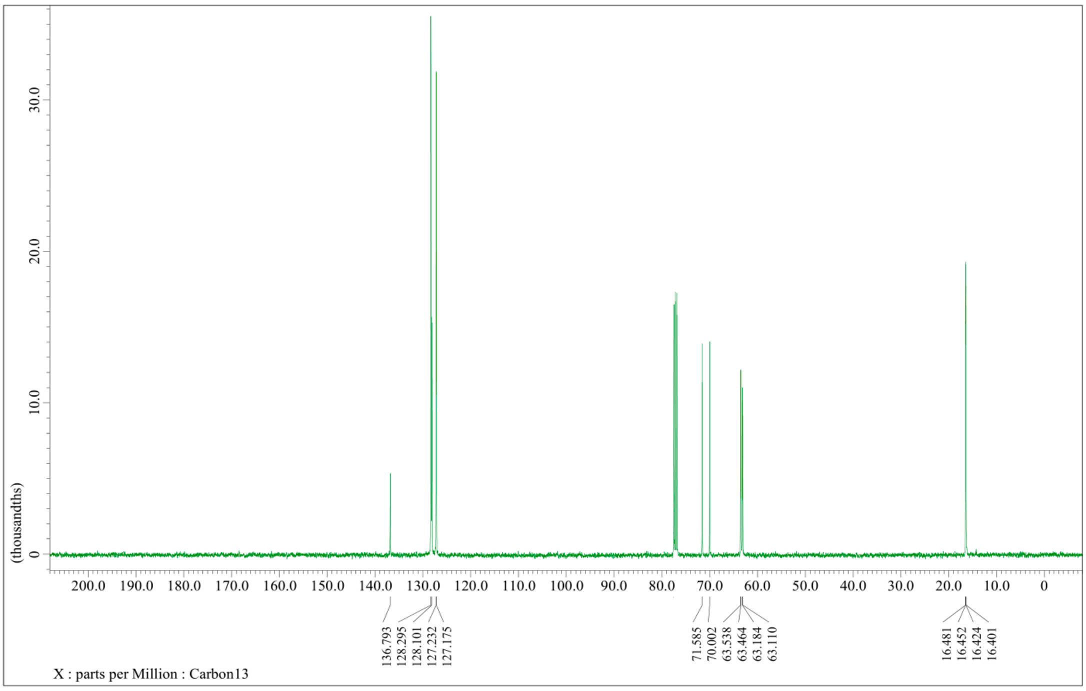

^{31}P NMR spectrum of **3a**

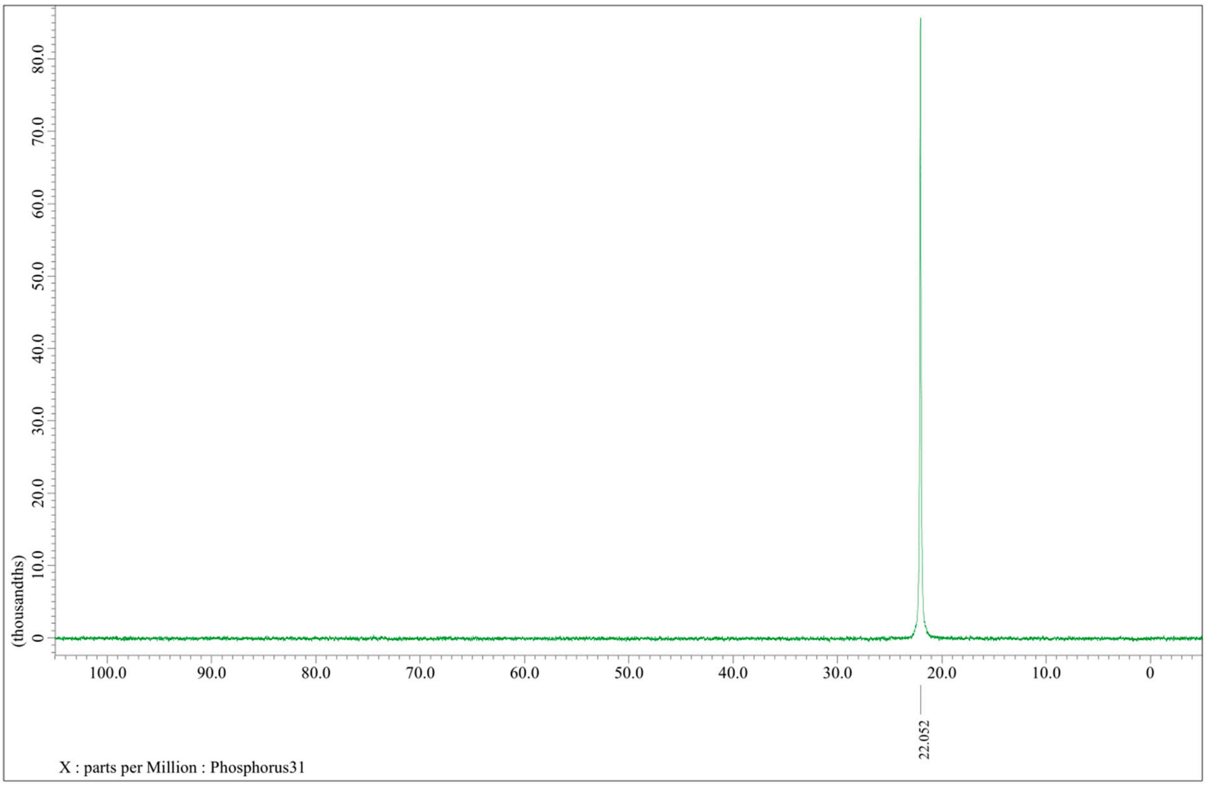

Diethyl (4-chloro-1-hydroxybenzyl)phosphonate (3b)

^{1}H NMR spectrum of **3b**

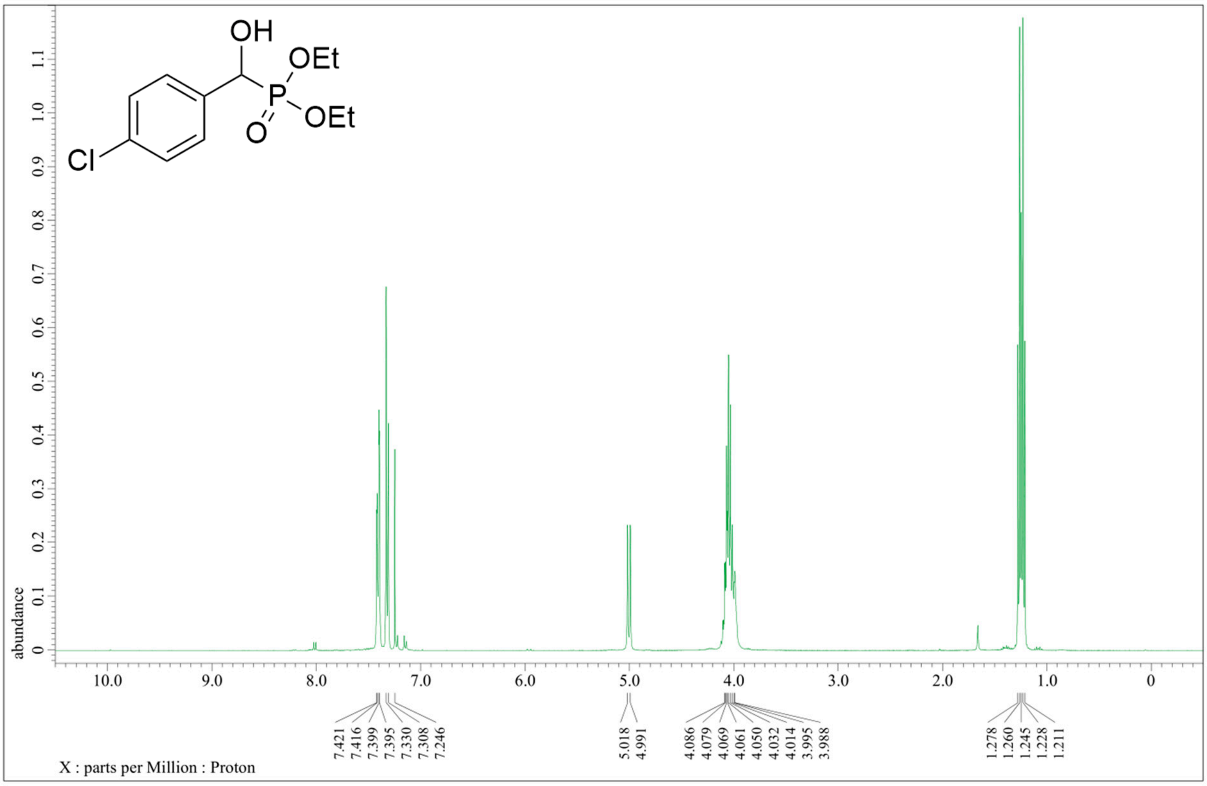

^{13}C NMR spectrum of **3b**

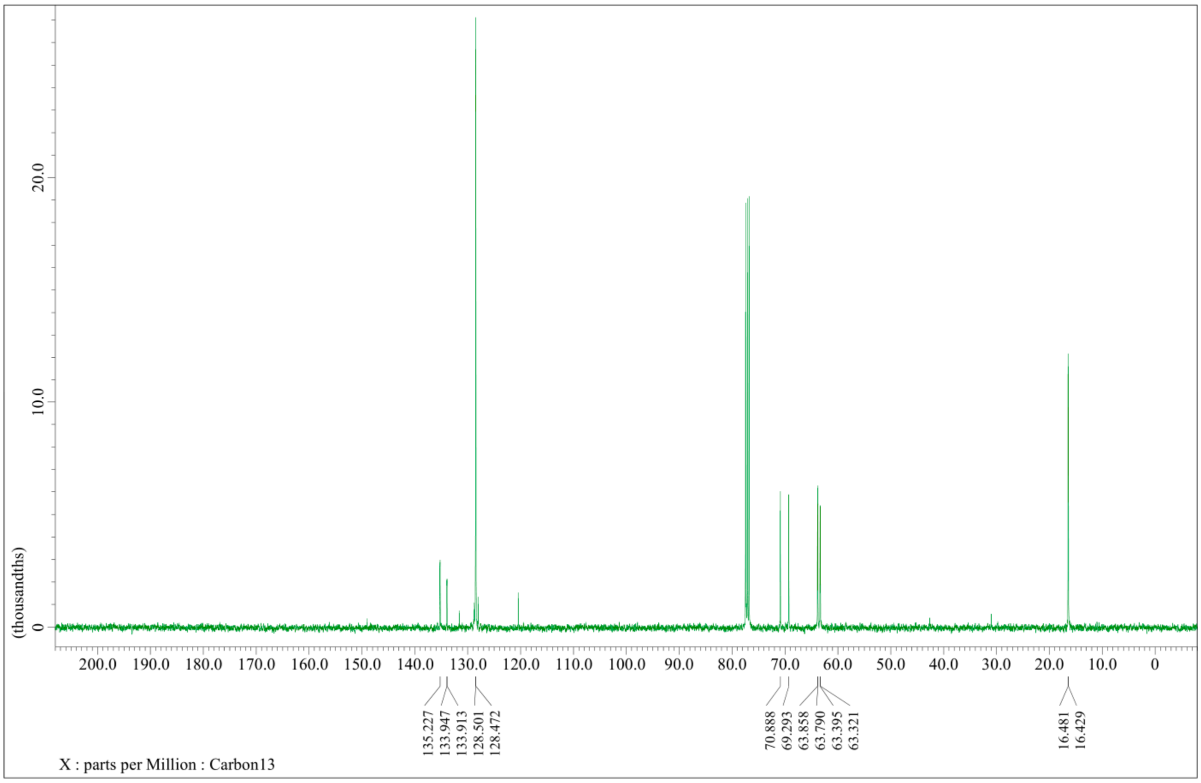

^{31}P NMR spectrum of **3b**

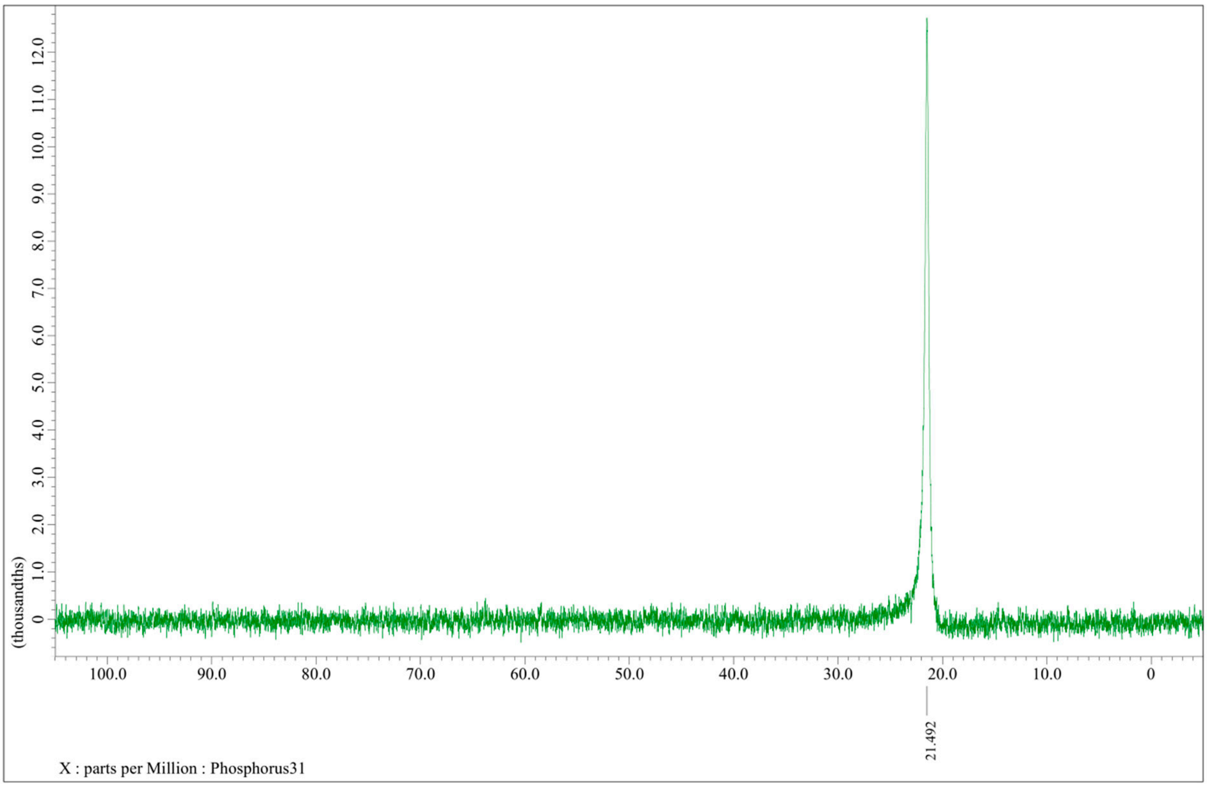

Diethyl (4-nitro-1-hydroxybenzyl)phosphonate (3c)

^{1}H NMR spectrum of **3c**

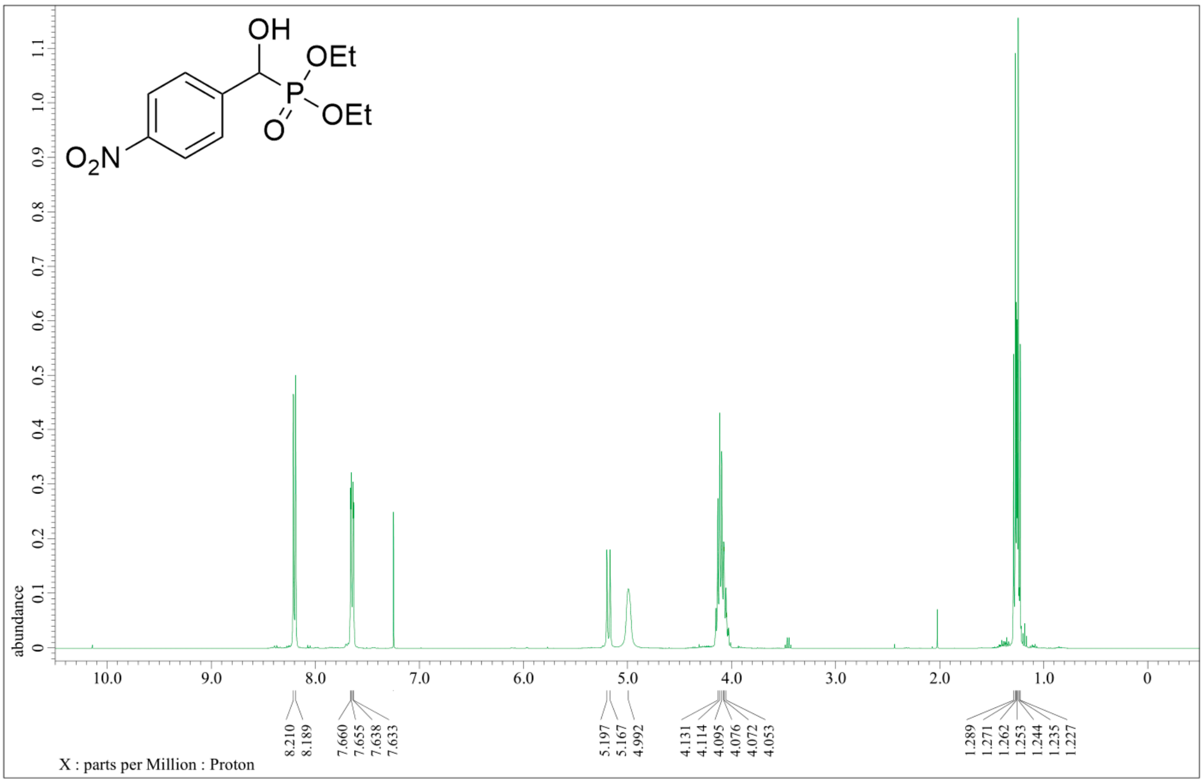

^{13}C NMR spectrum of **3c**

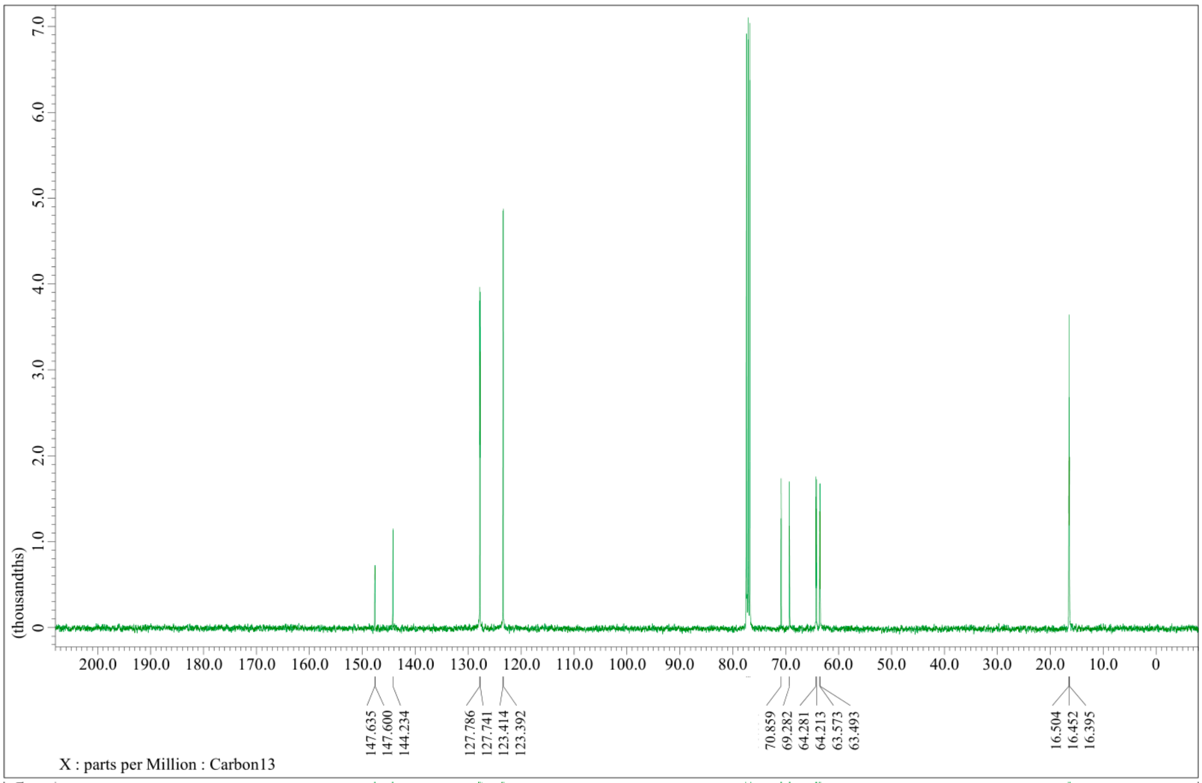

^{31}P NMR spectrum of **3c**

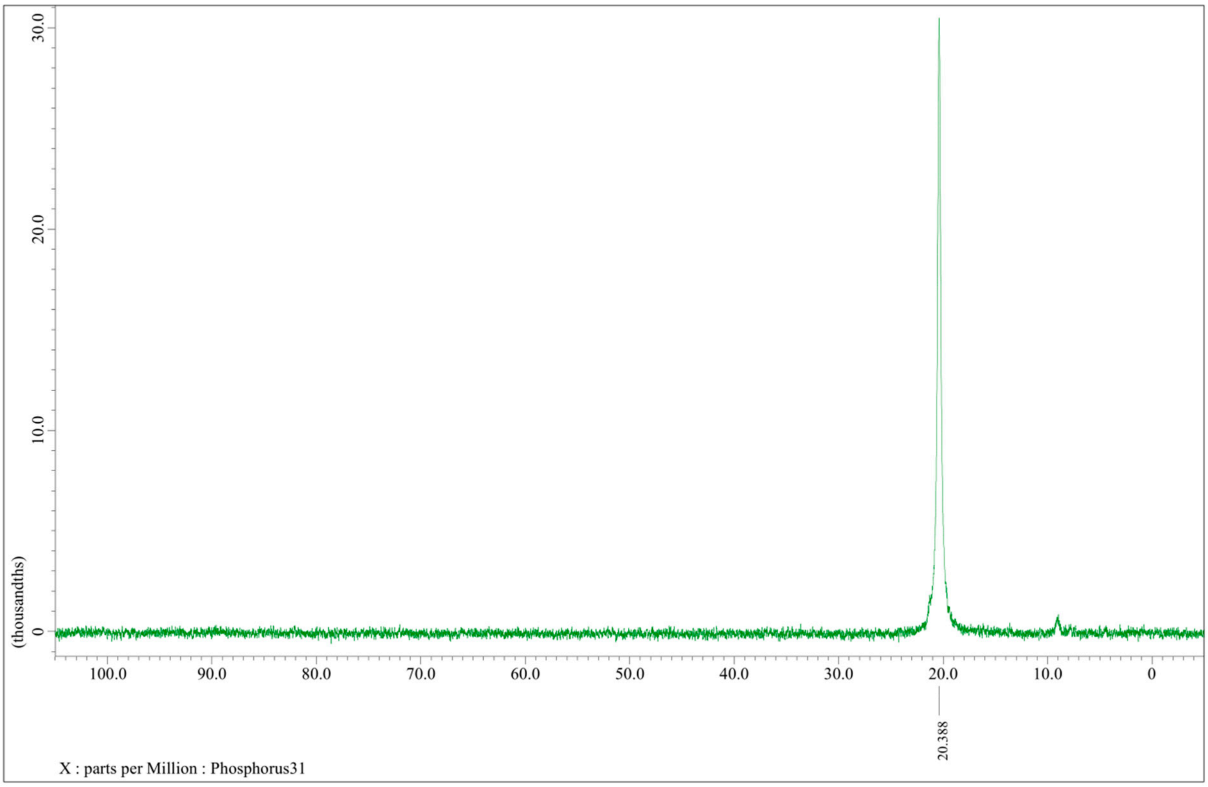

Diethyl (4-methyl-1-hydroxybenzyl)phosphonate (**3d**)

^{1}H NMR spectrum of **3d**

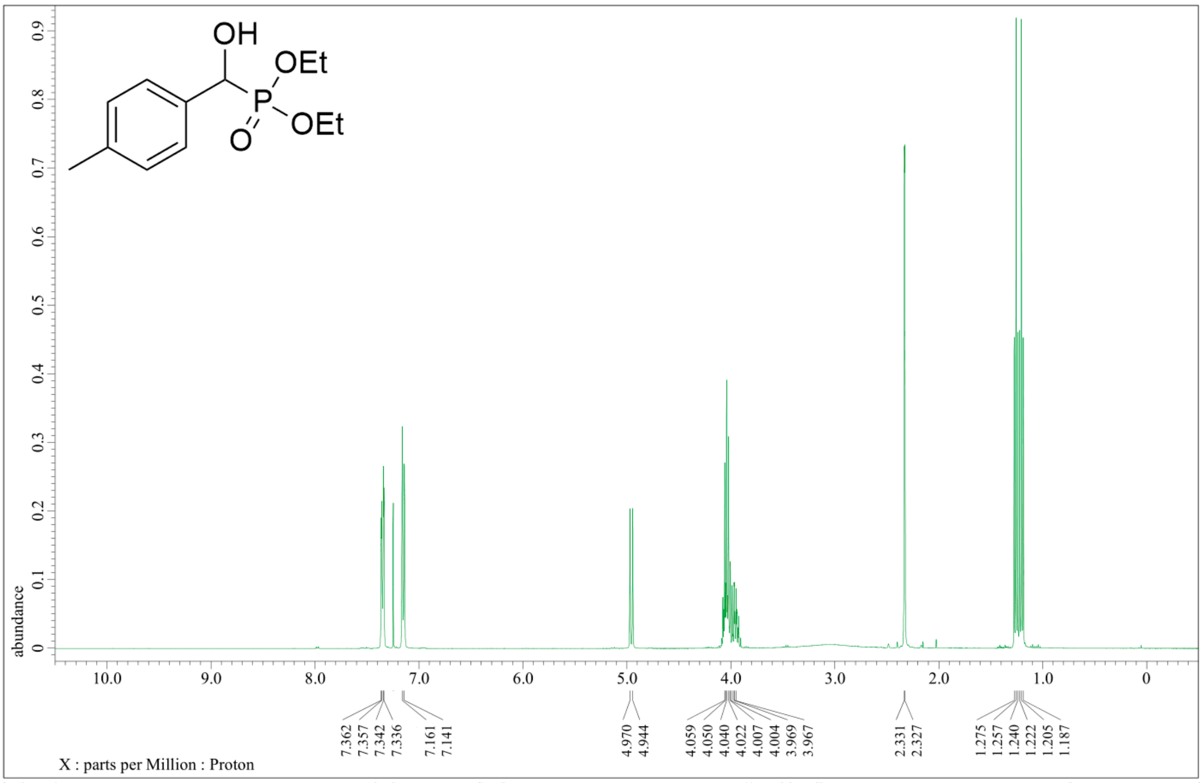

^{13}C NMR spectrum of **3d**

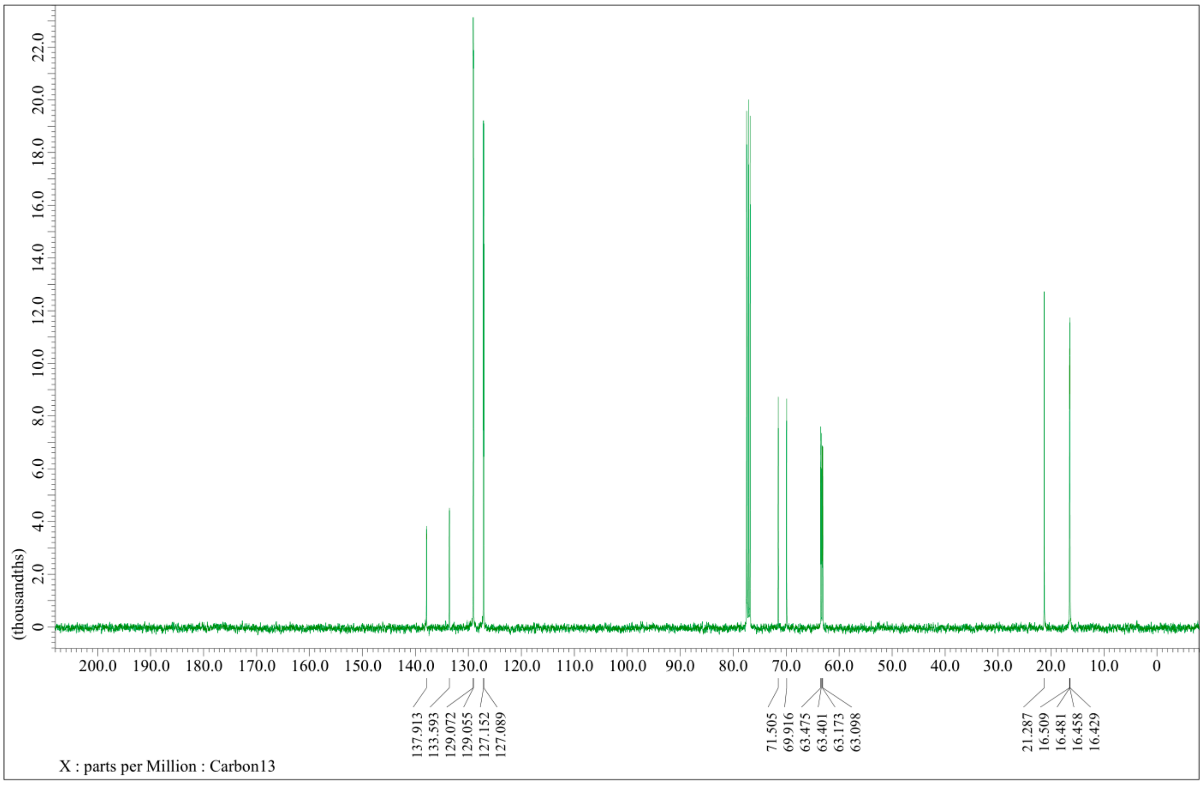

^{31}P NMR spectrum of **3d**

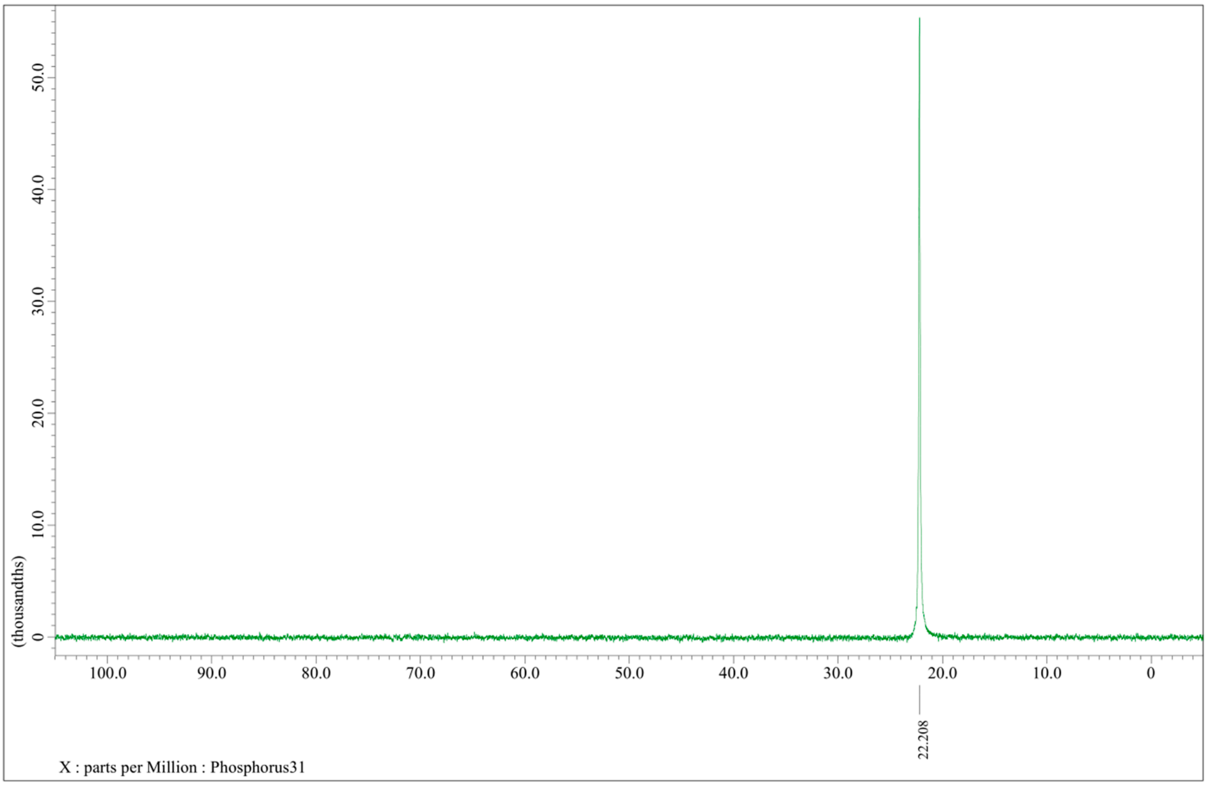

Diethyl (2,4,6-trimethyl-1-hydroxybenzyl)phosphonate (3e)

^{1}H NMR spectrum of **3e**

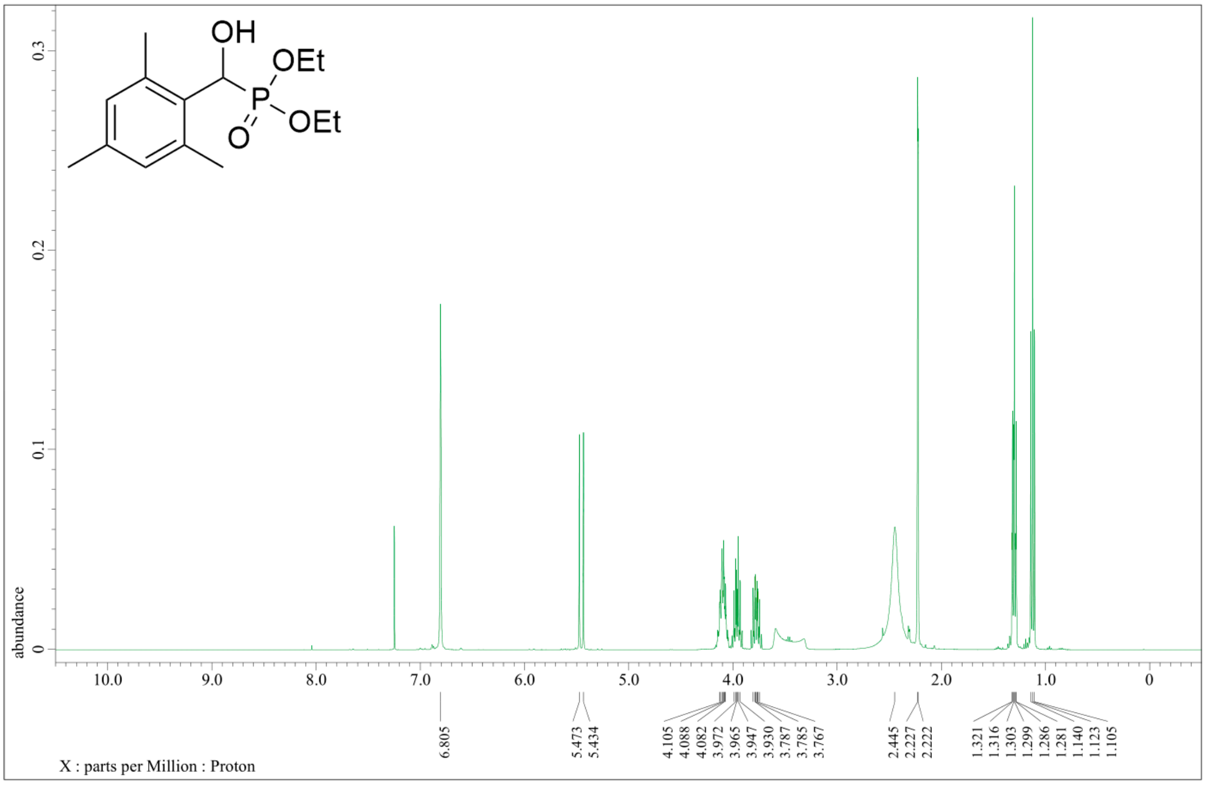

^{13}C NMR spectrum of **3e**

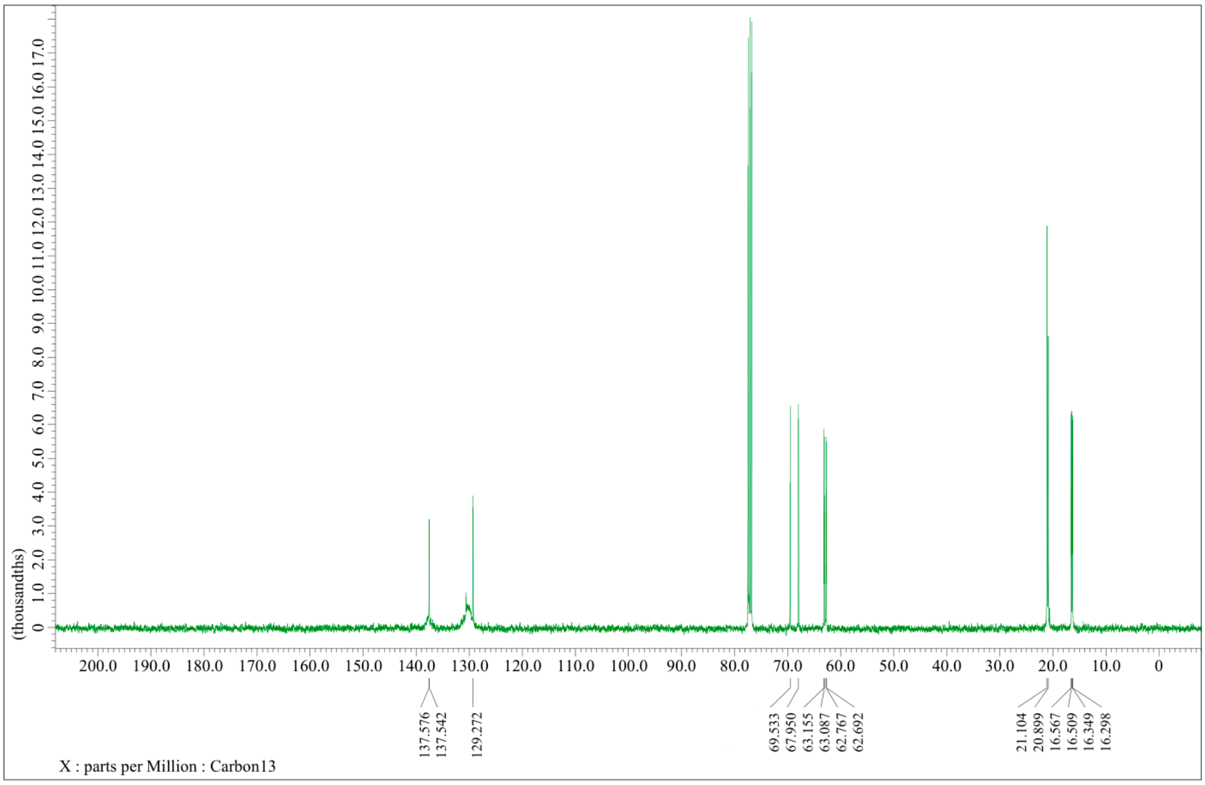

^{31}P NMR spectrum of **3e**

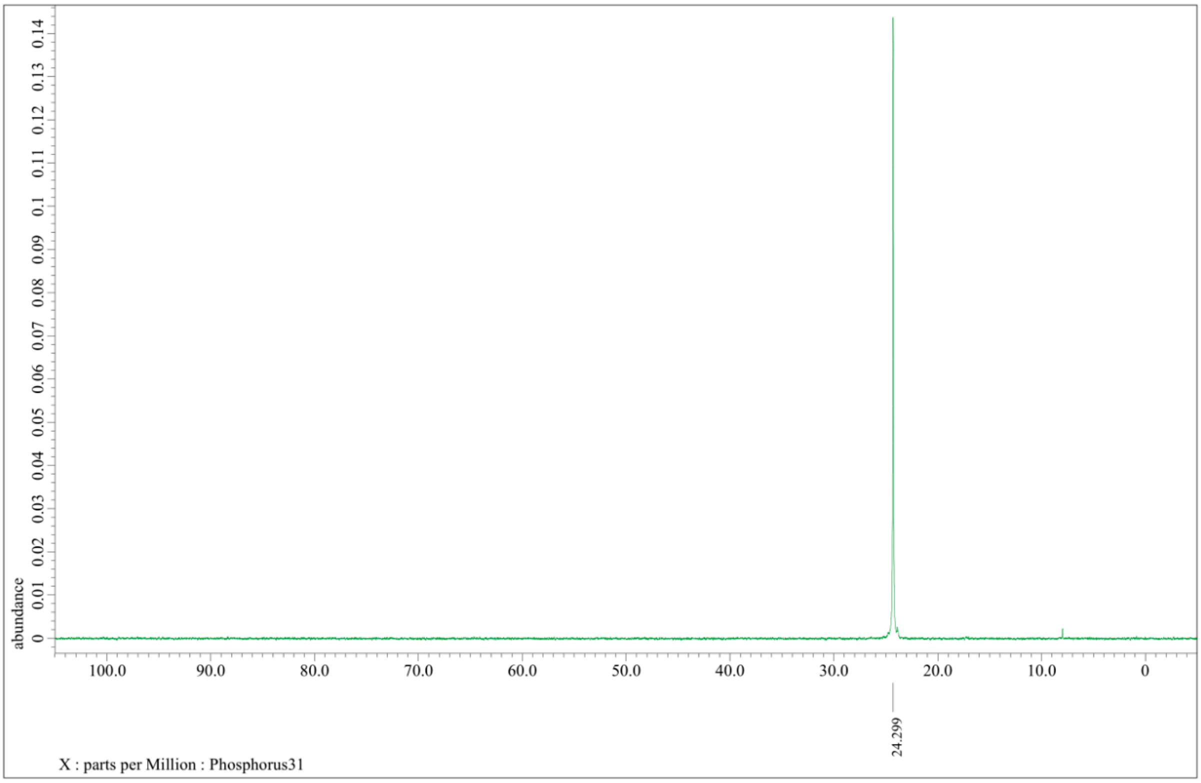

Diethyl 1-hydroxy-3-methyl-butylphosphonate (3f)

^{1}H NMR spectrum of **3f**

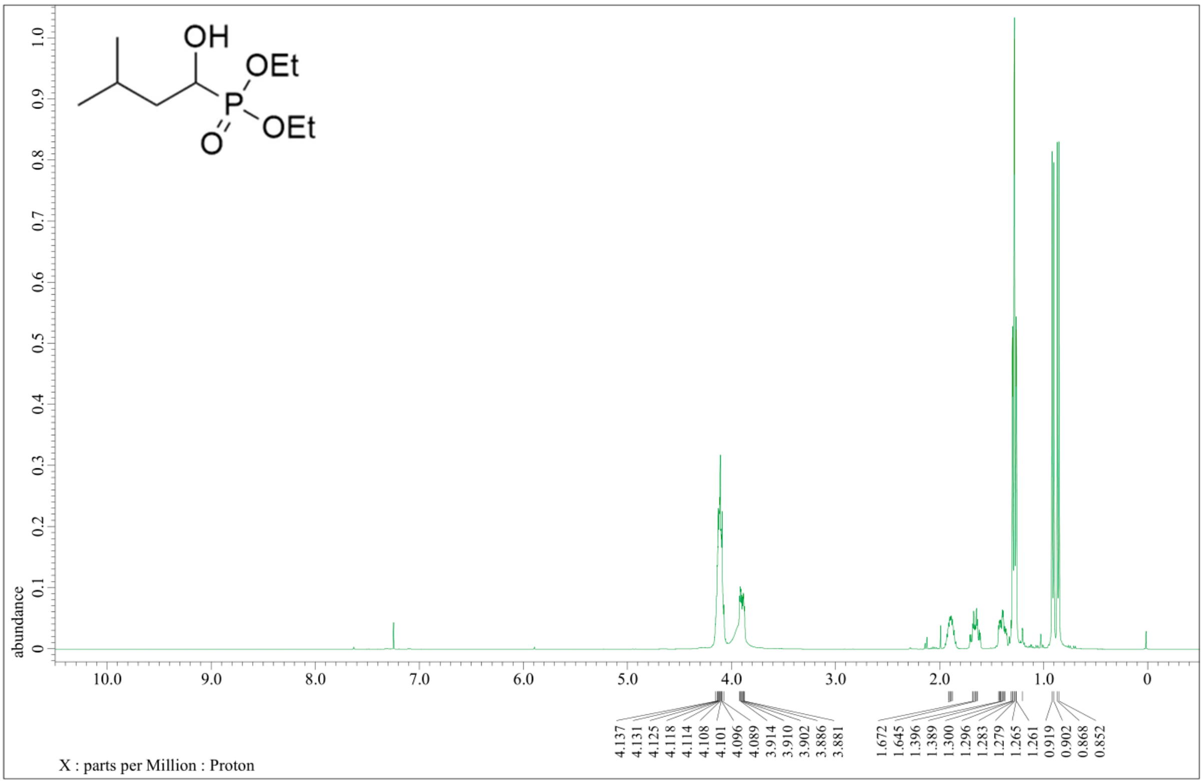

^{13}C NMR spectrum of **3f**

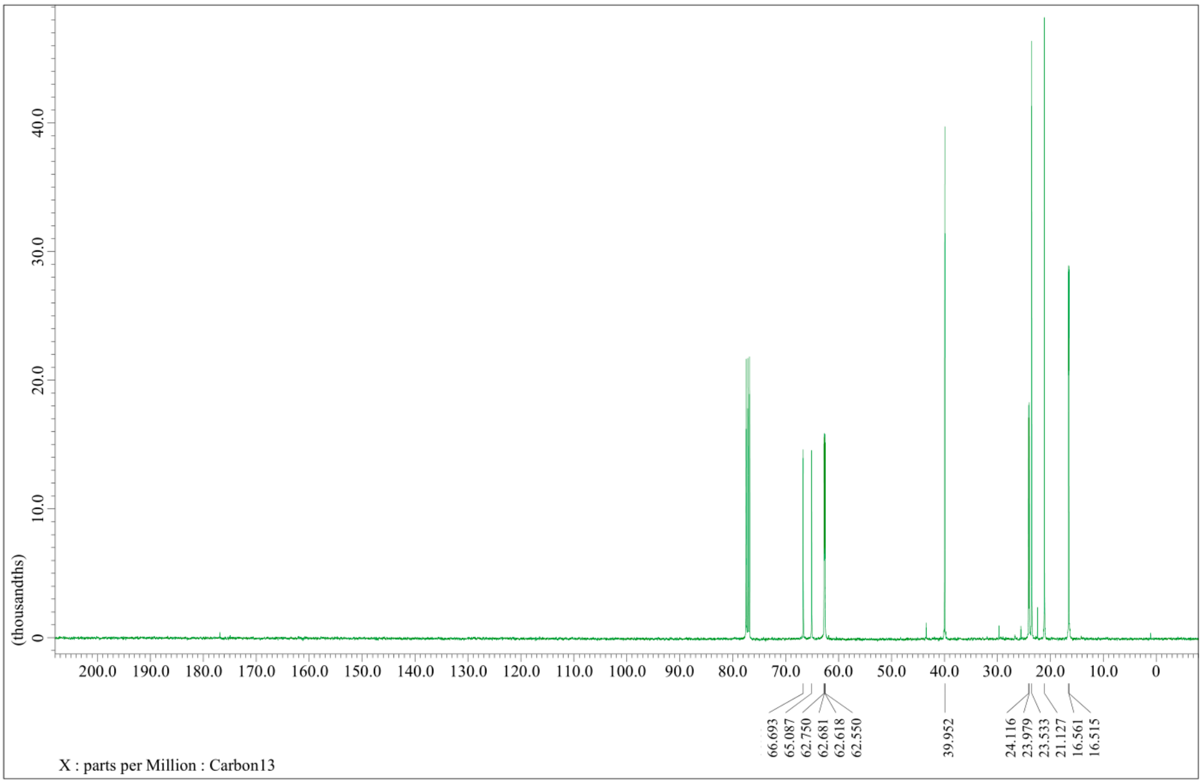

^{31}P NMR spectrum of **3f**

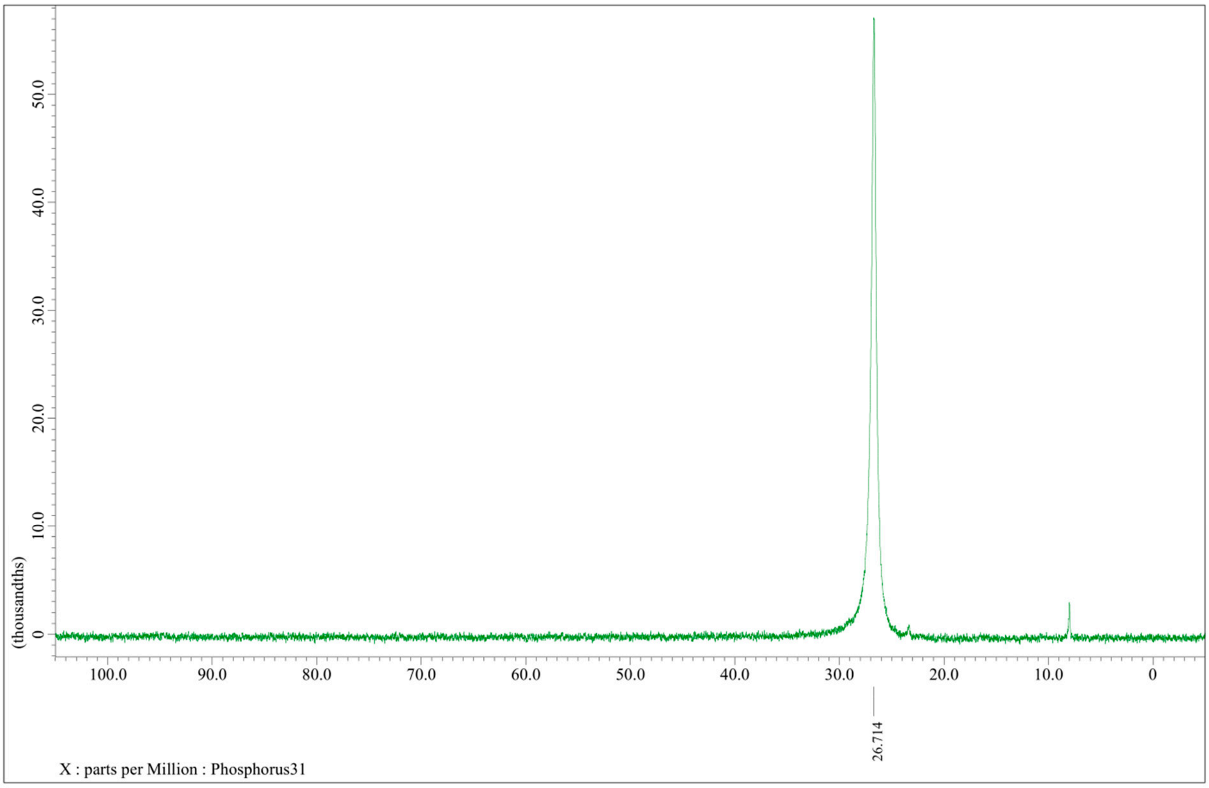

Diethyl 1-hydroxypropylphosphonate (**3g**)

^{1}H NMR spectrum of **3g**

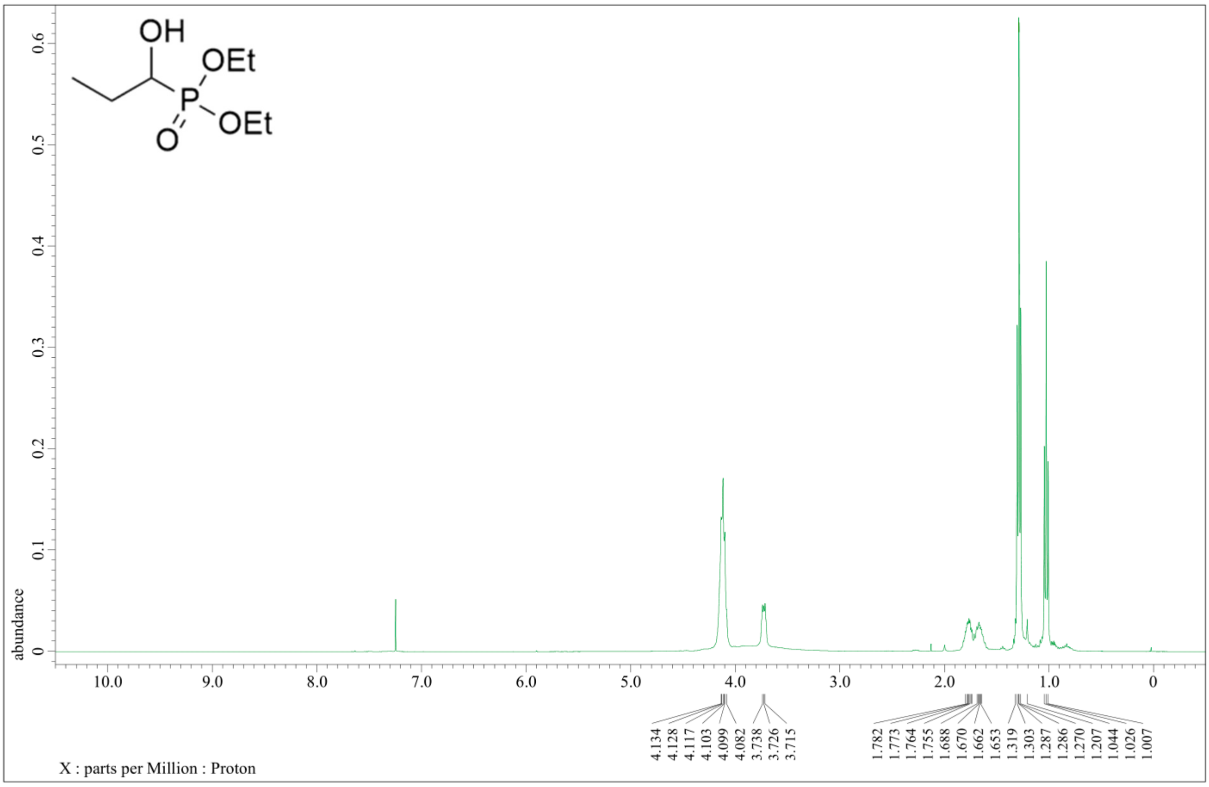

^{13}C NMR spectrum of **3g**

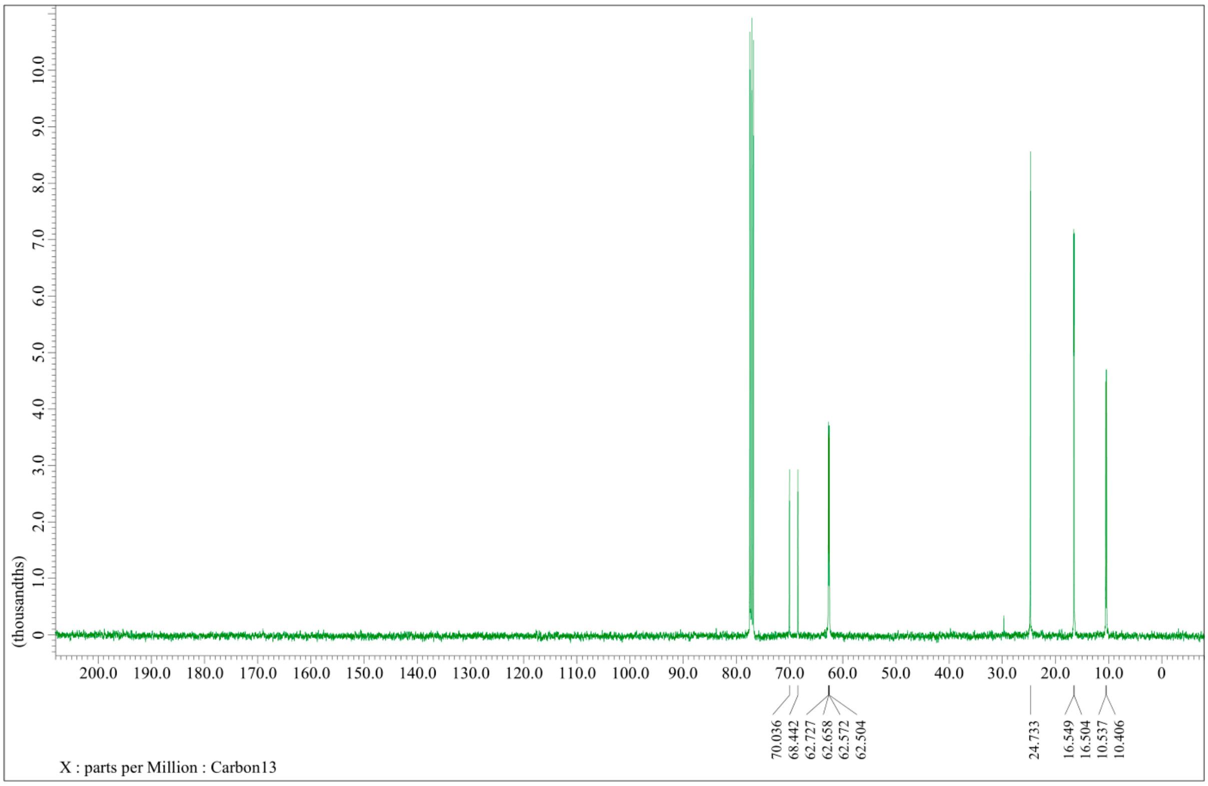

^{31}P NMR spectrum of **3g**

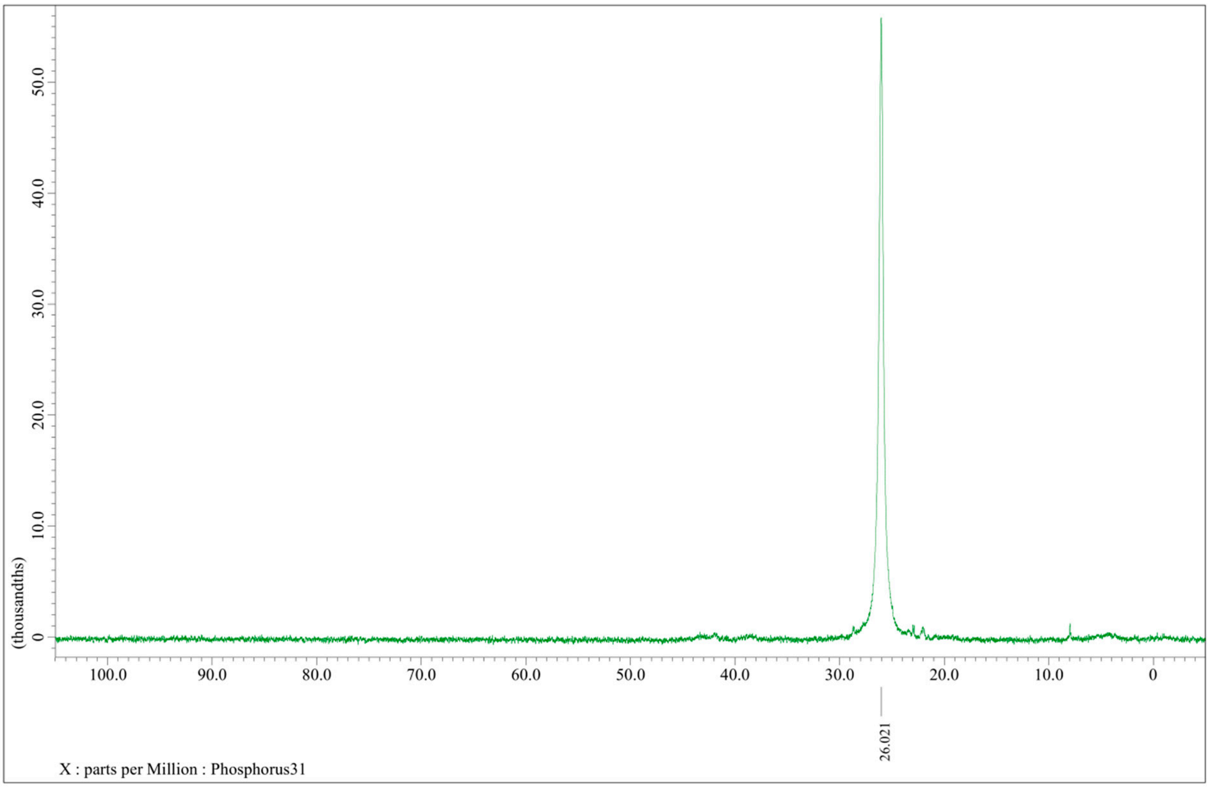

Diethyl 1-hydroxy-2-methyl-propylphosphonate (3h)

^{1}H NMR spectrum of **3h**

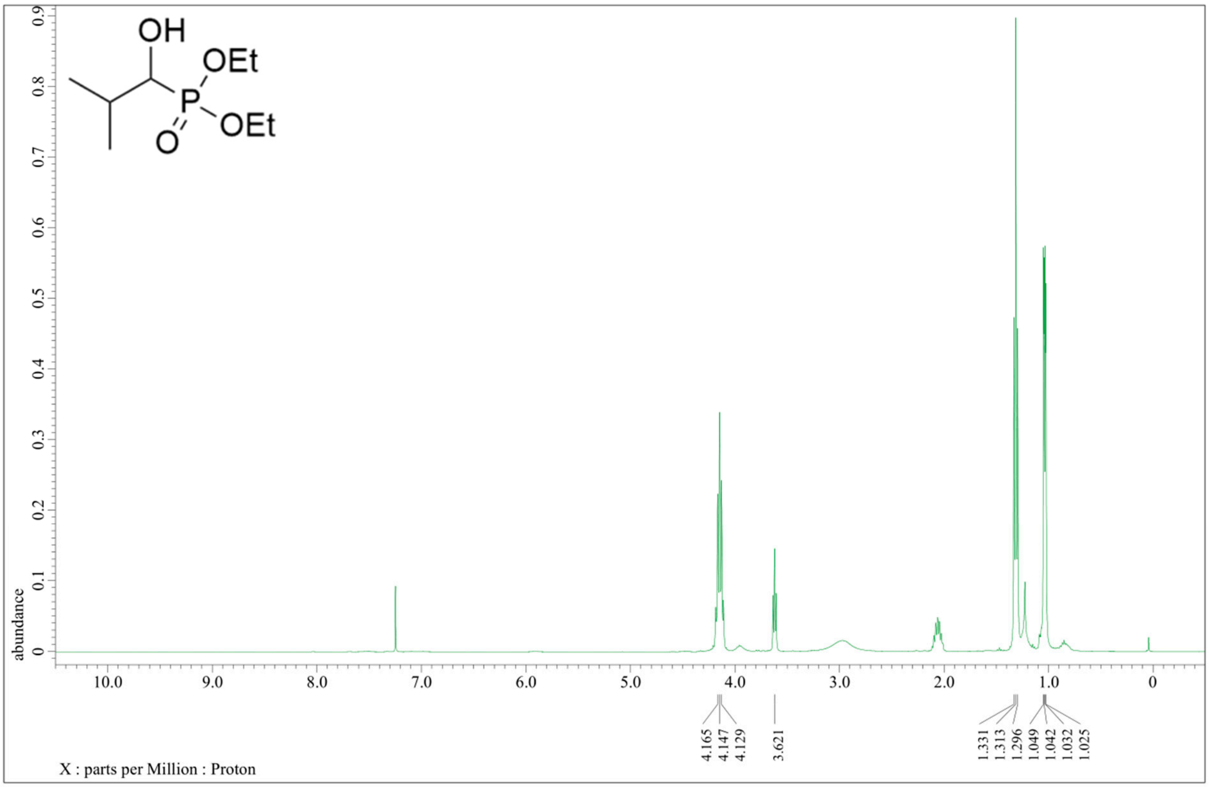

^{13}C NMR spectrum of **3h**

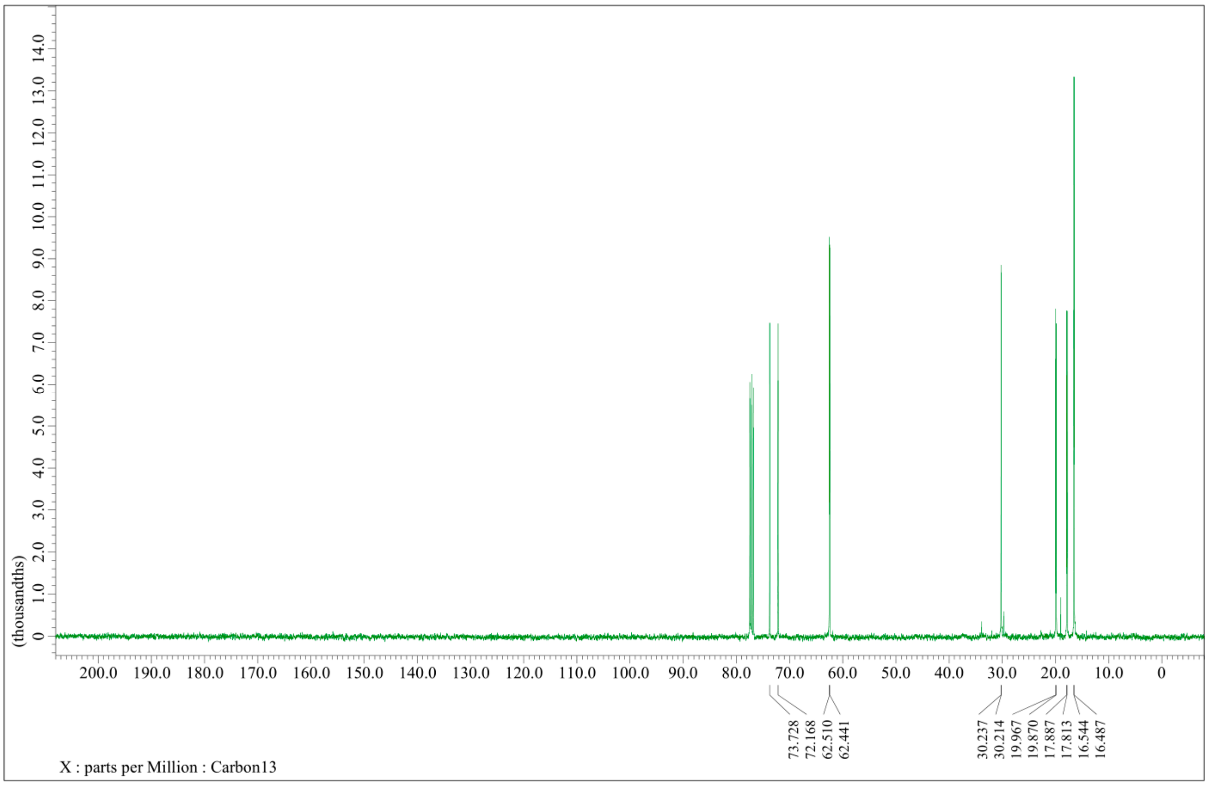

^{31}P NMR spectrum of **3h**

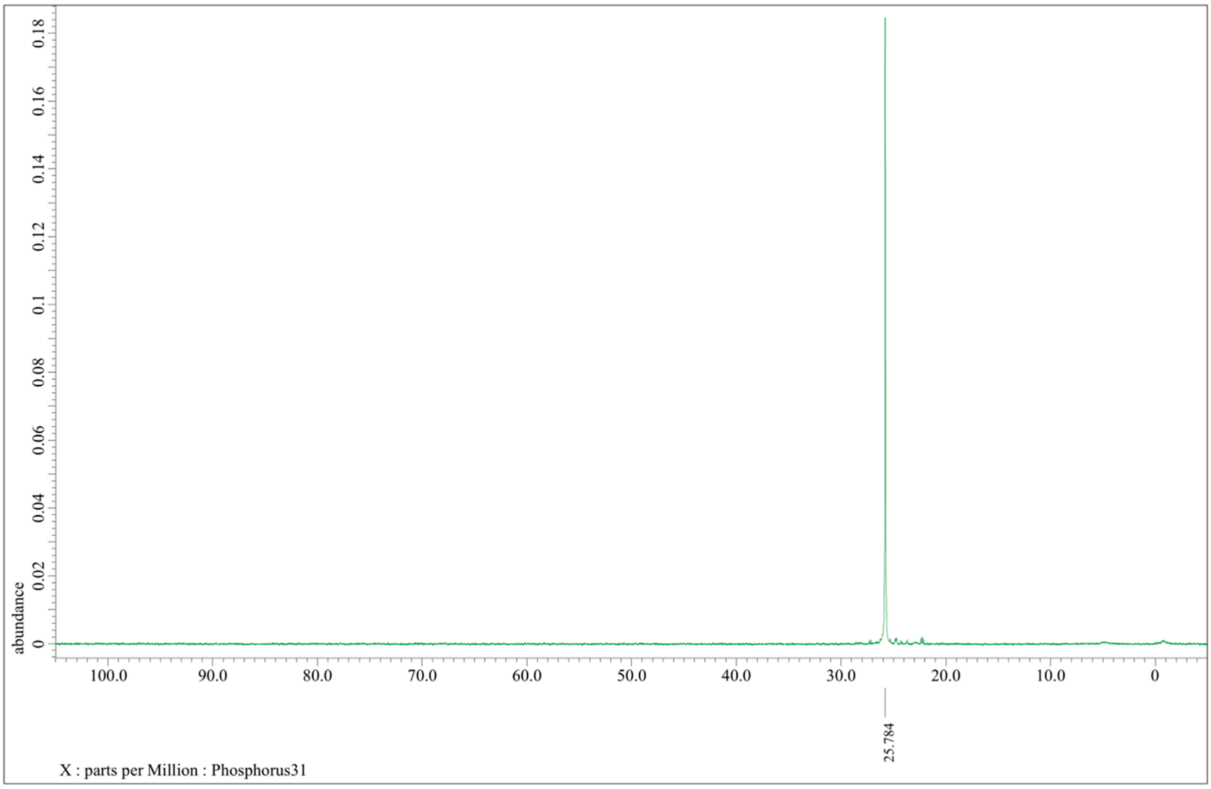

Diethyl 2-ethyl-1-hydroxybutylphosphonate (**3i**)

^{1}H NMR spectrum of **3i**

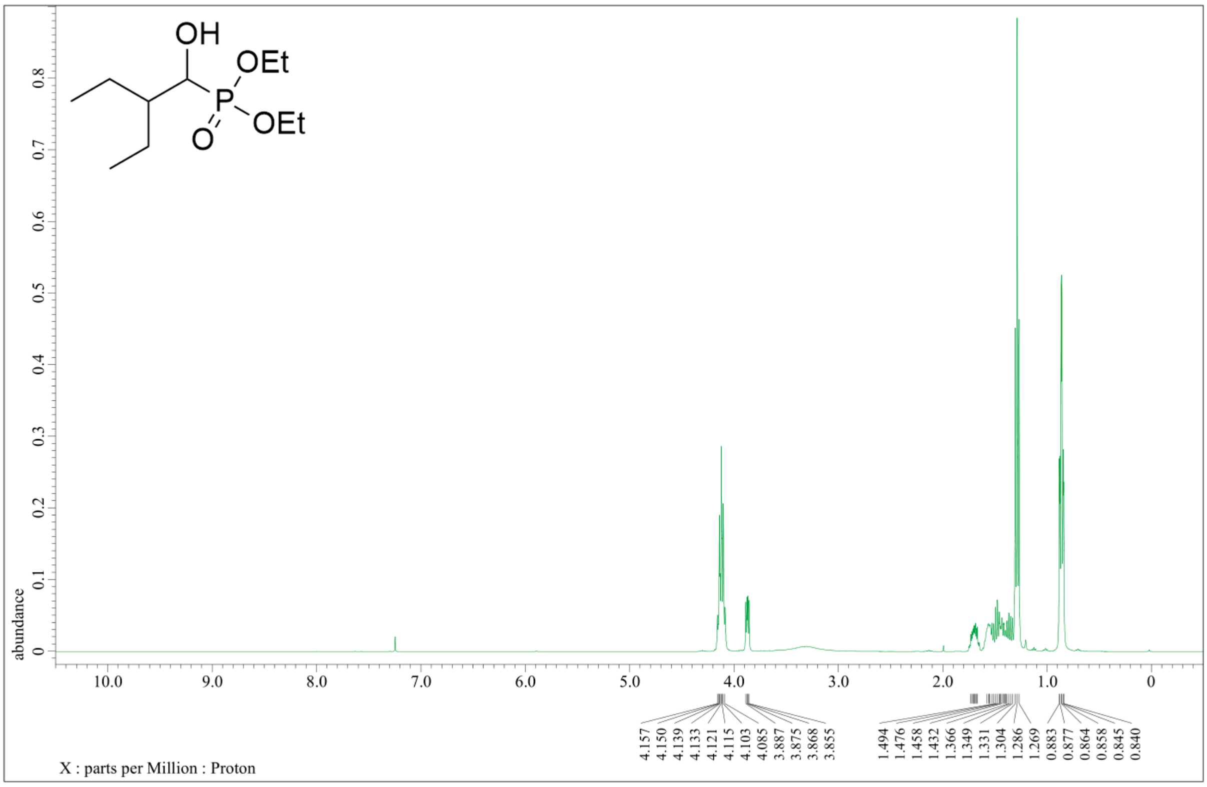

^{13}C NMR spectrum of **3i**

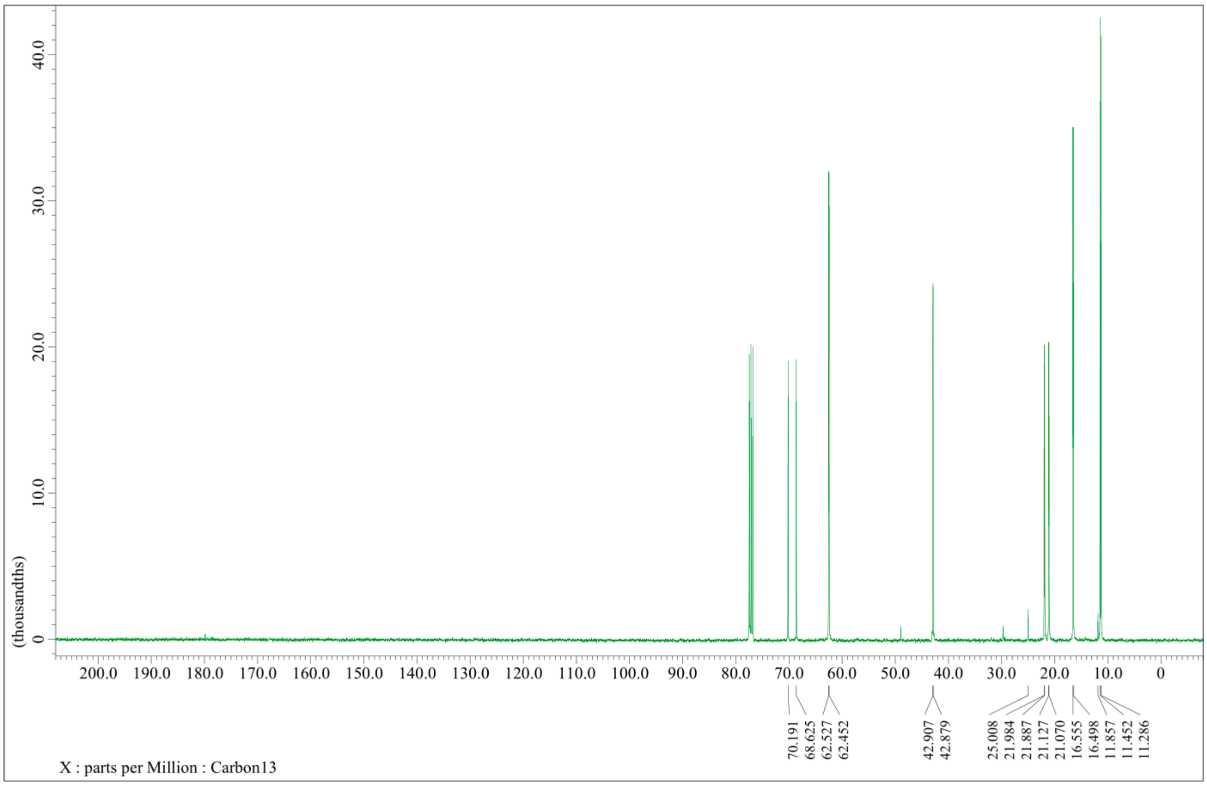

^{31}P NMR spectrum of **3i**

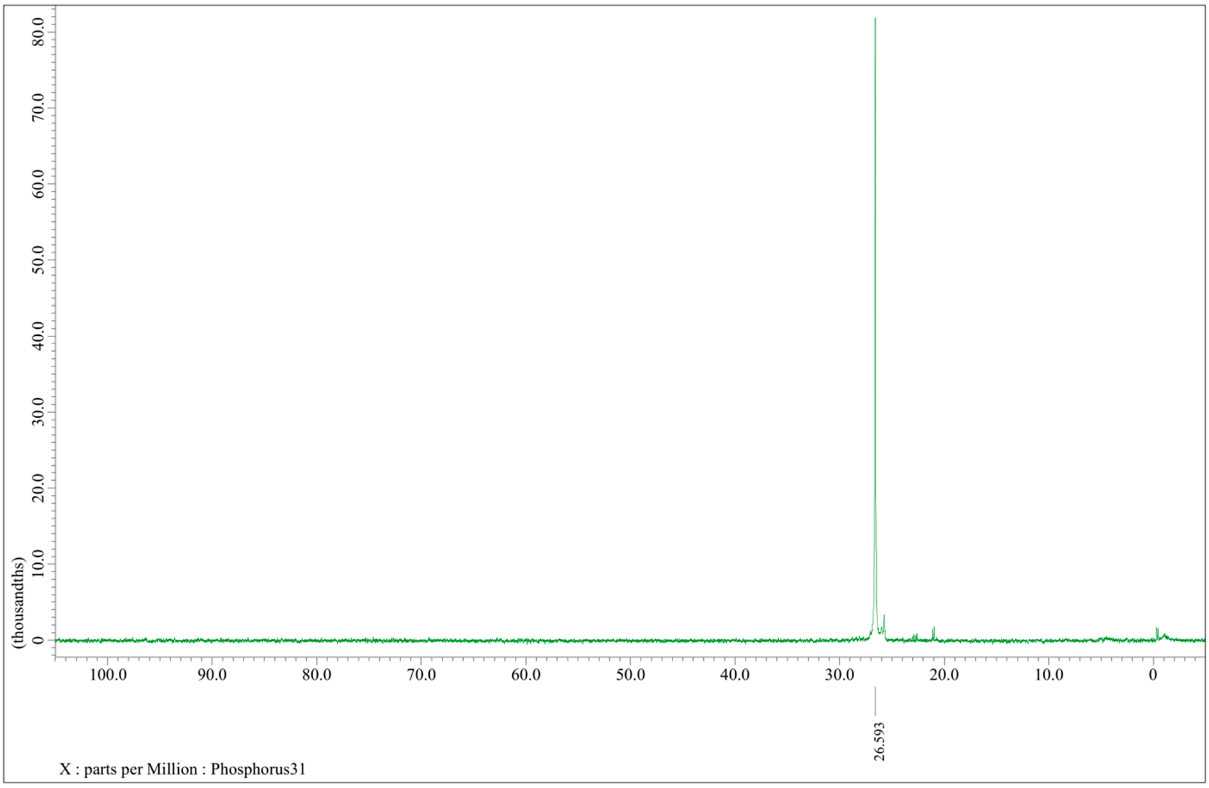

Diethyl 1-hydroxy(pyridine-3-yl)methylphosphonate **(3j)**

^{1}H NMR spectrum of **3j**

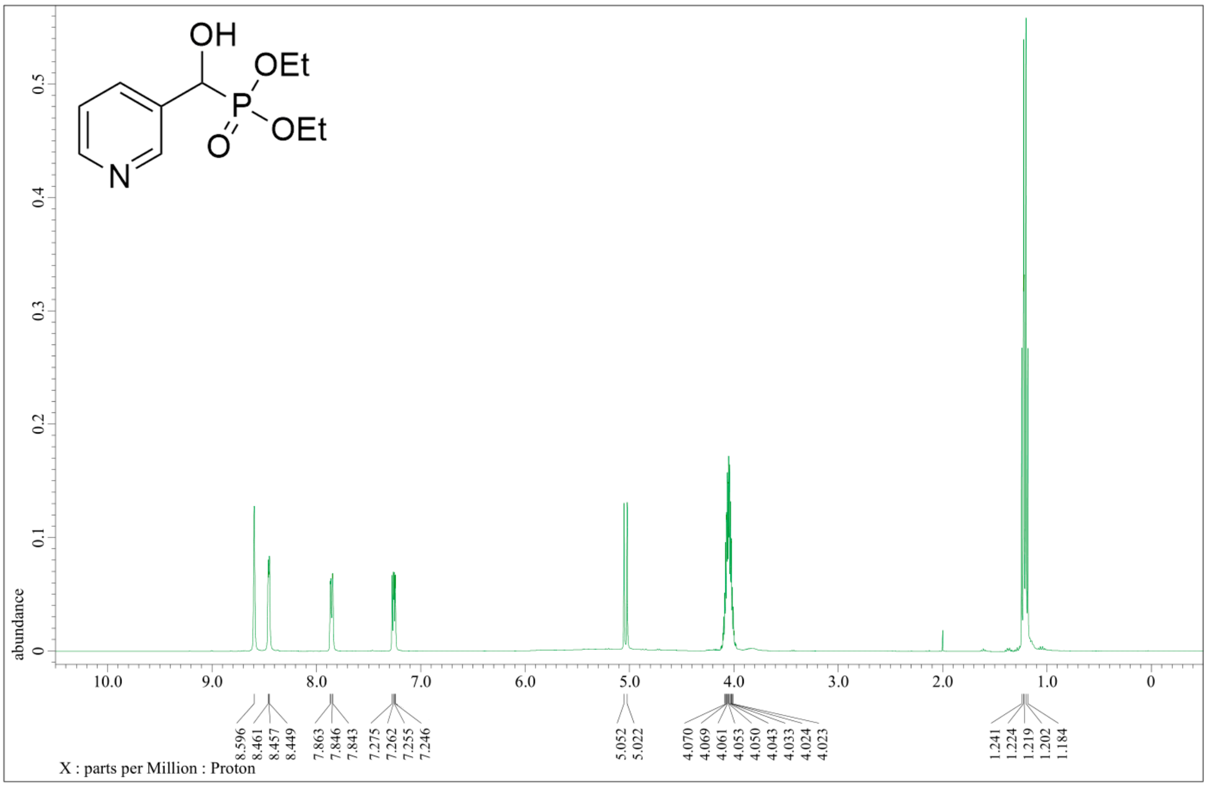

^{13}C NMR spectrum of **3j**

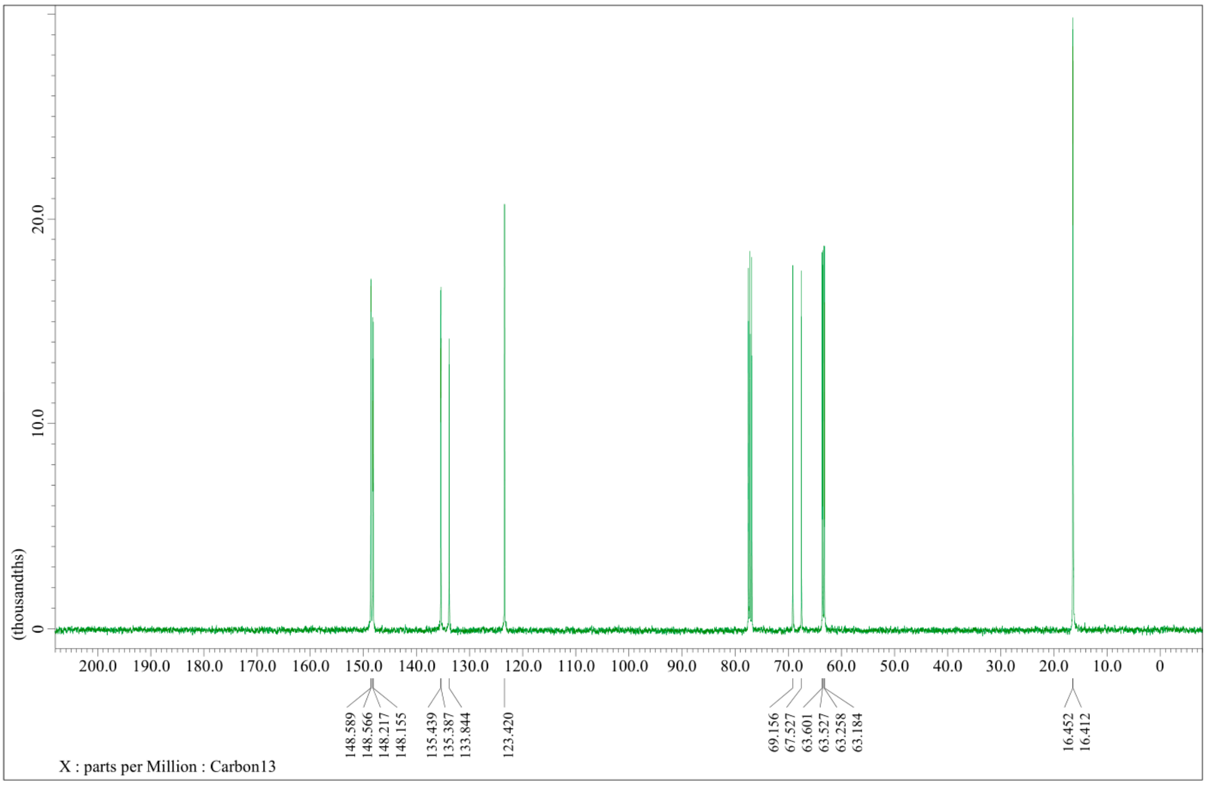

^{31}P NMR spectrum of **3j**

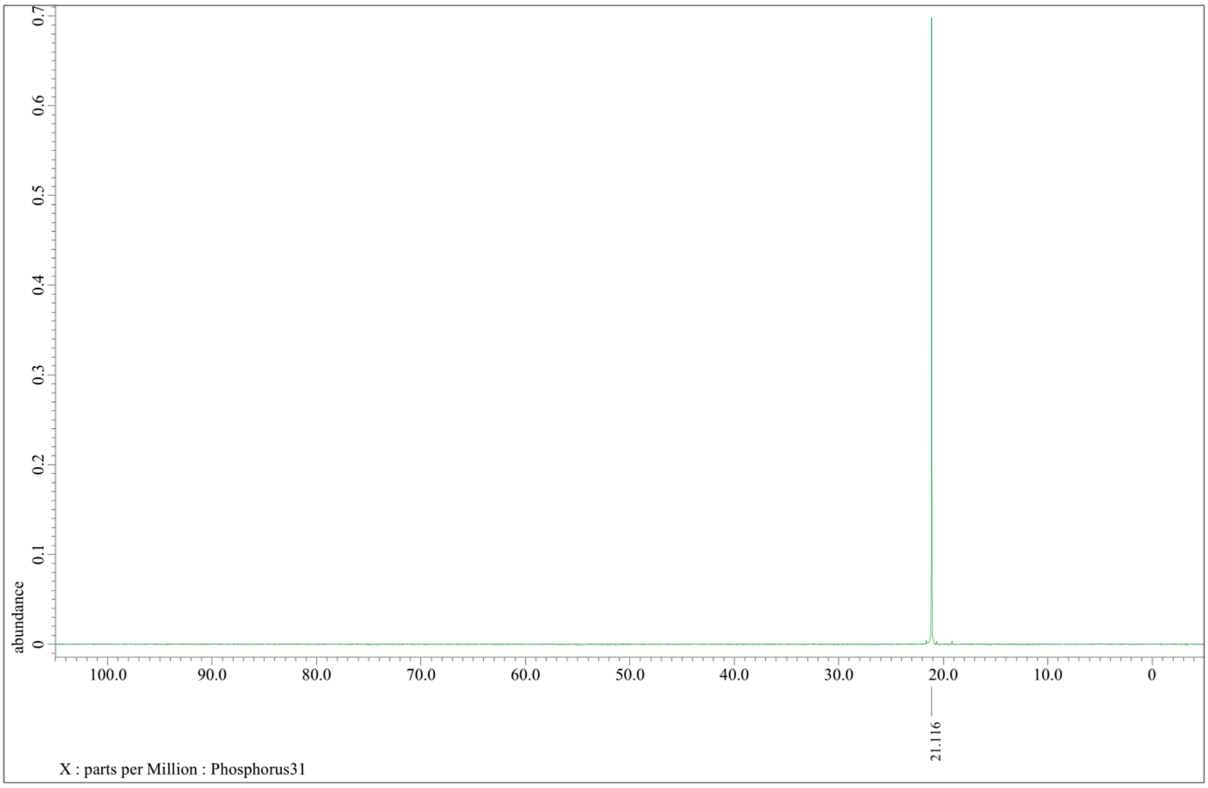

4 Diethyl 1-hydroxy(quinolin-3-yl)methylphosphonate (3k)

^{1}H NMR spectrum of **3k**

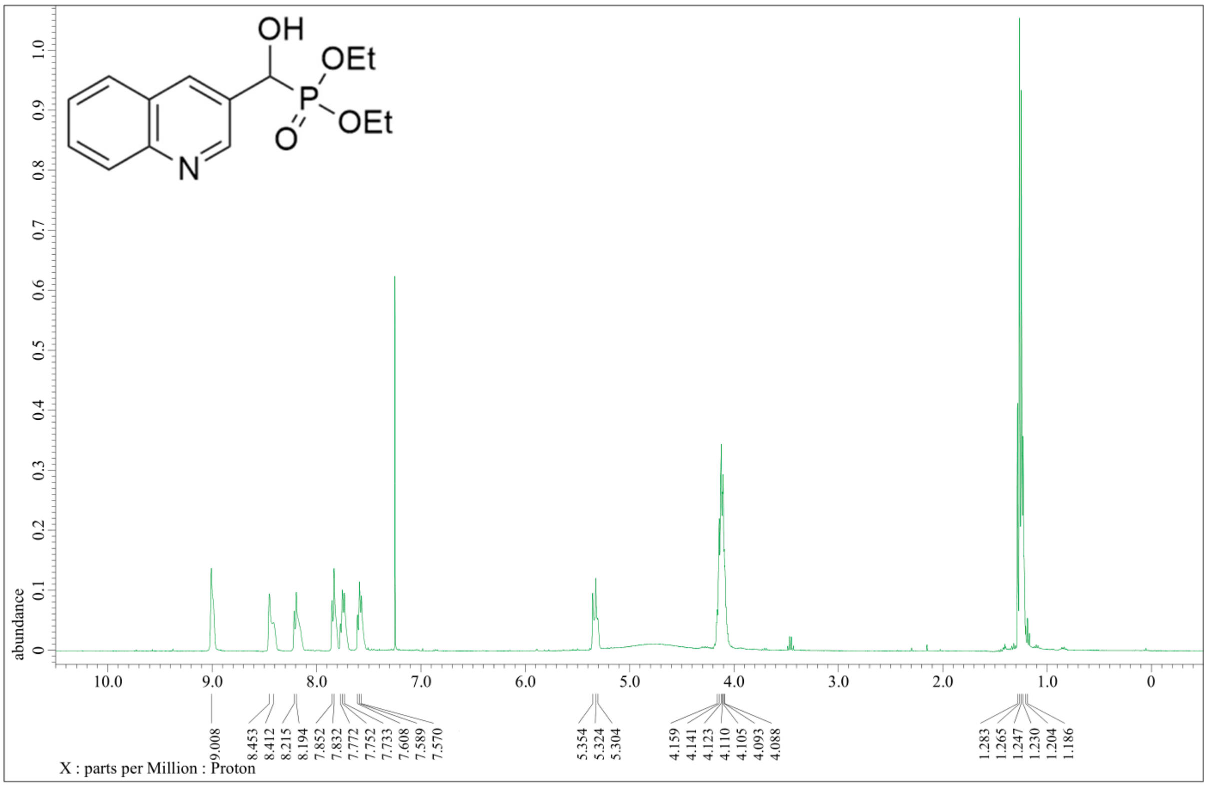

^{13}C NMR spectrum of **3k**

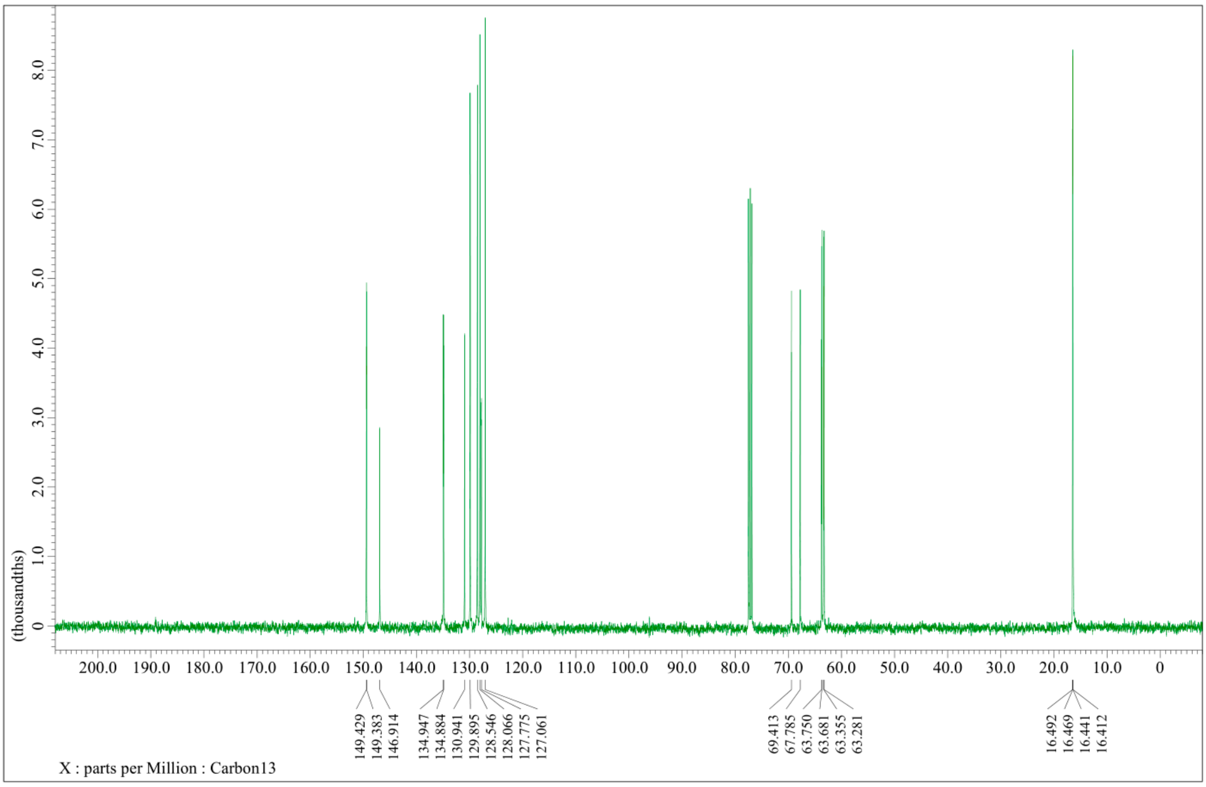

^{31}P NMR spectrum of **3k**

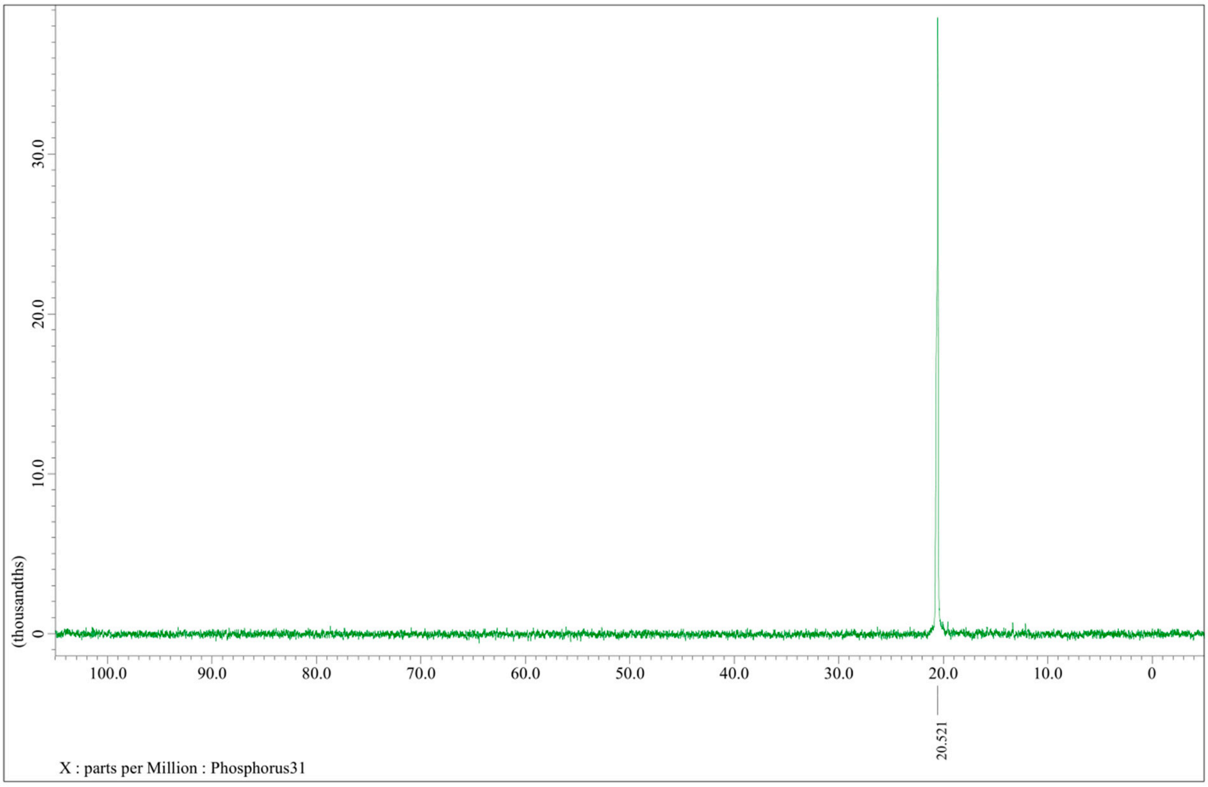

Diethyl 1-hydroxy(thiophen-2-yl)methylphosphonate (3l)

^{1}H NMR spectrum of **3l**

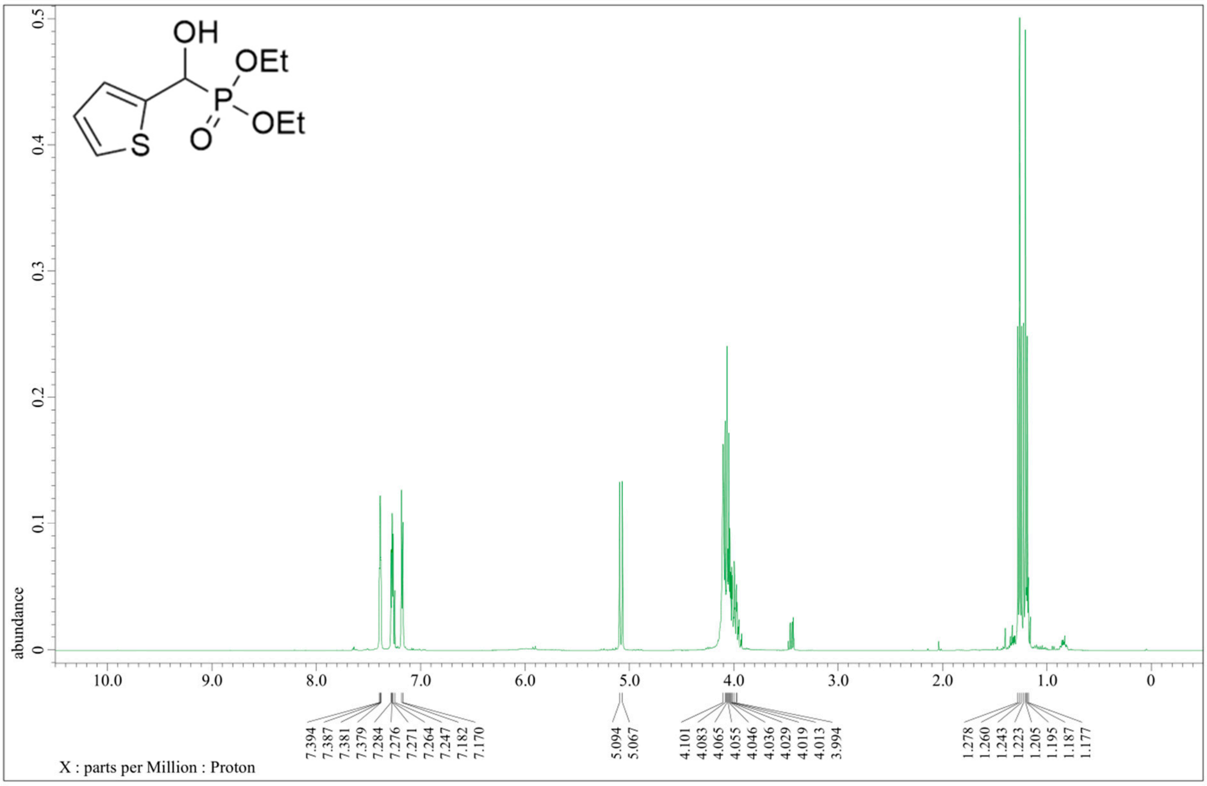

^{13}C NMR spectrum of **3l**

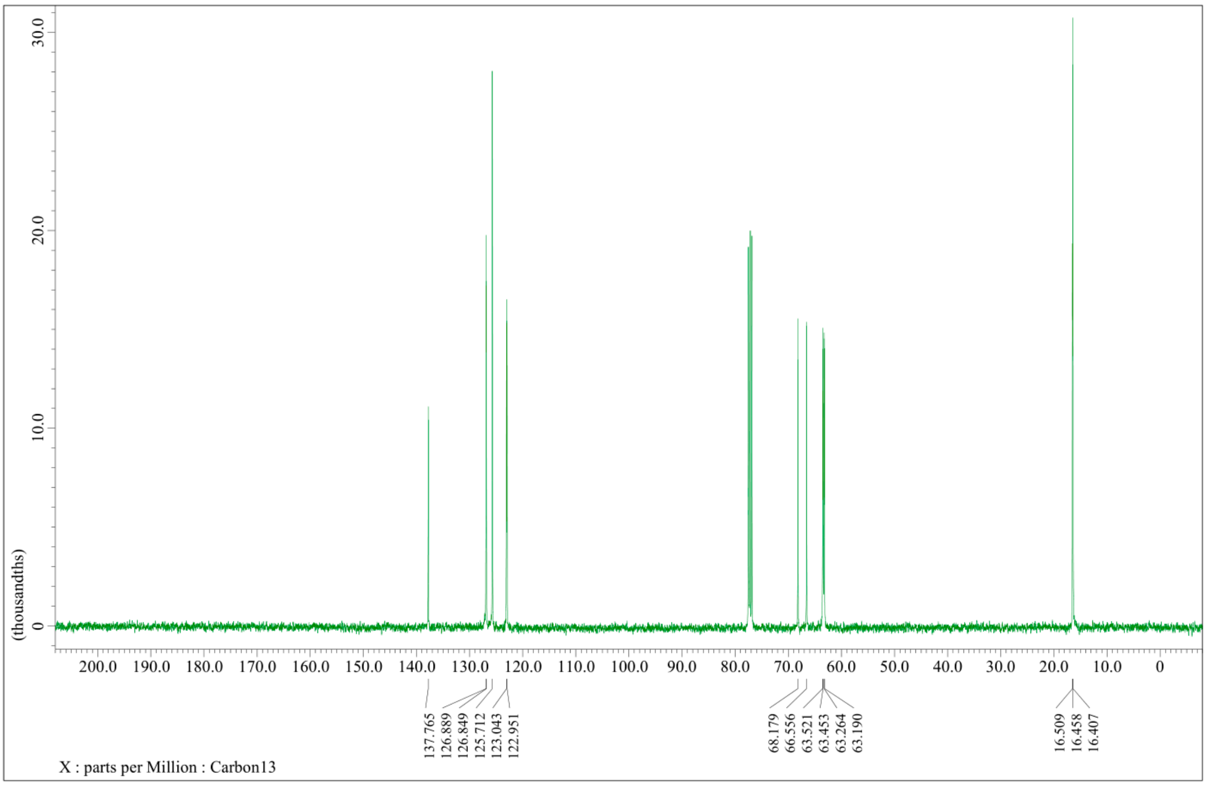

^{31}P NMR spectrum of **3l**

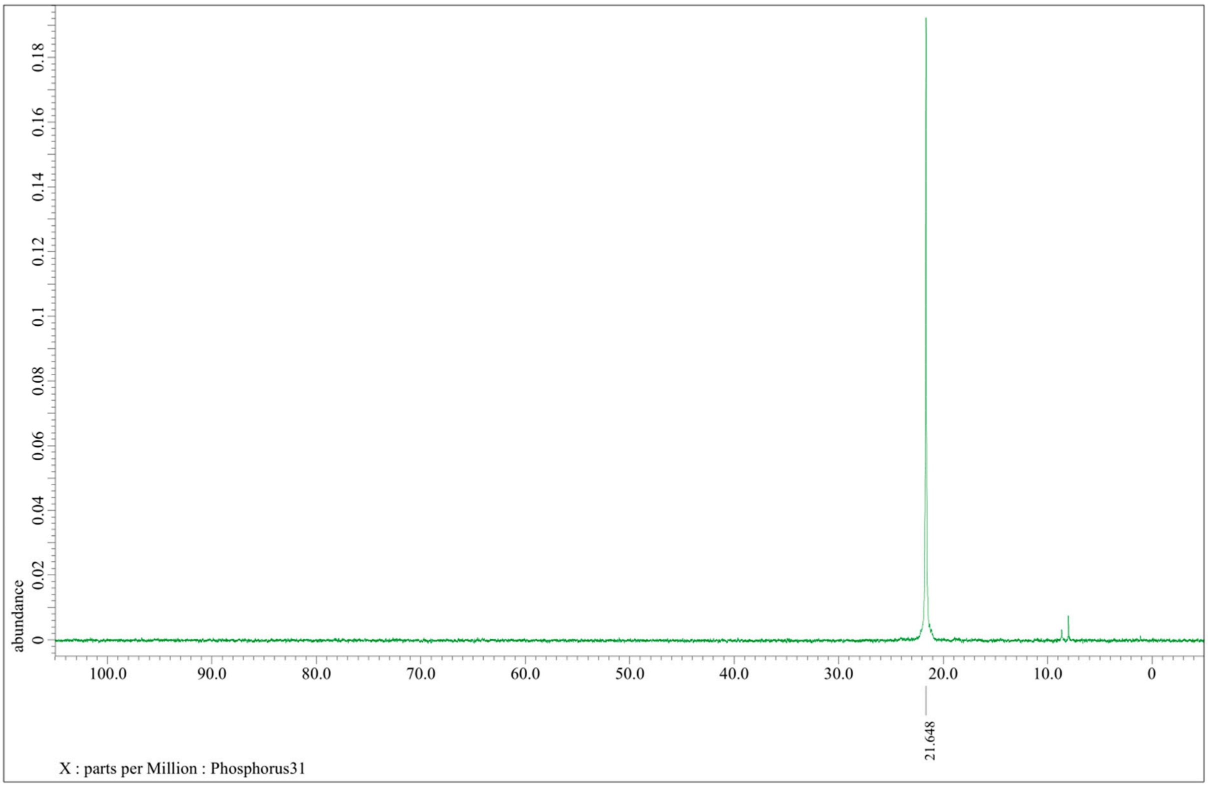

Diethyl (1-hydroxy-3-phenyl)prop-2-enyl-phosphonate (3m)

^{1}H NMR spectrum of **3m**

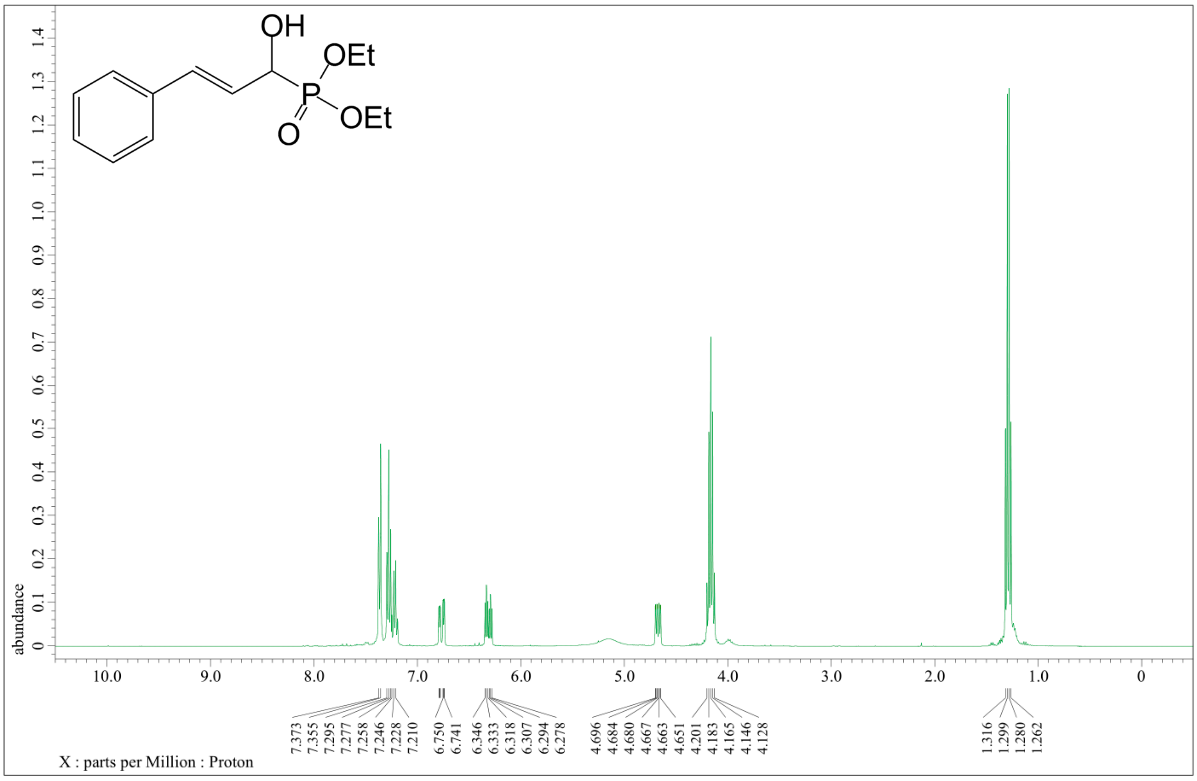

^{13}C NMR spectrum of **3m**

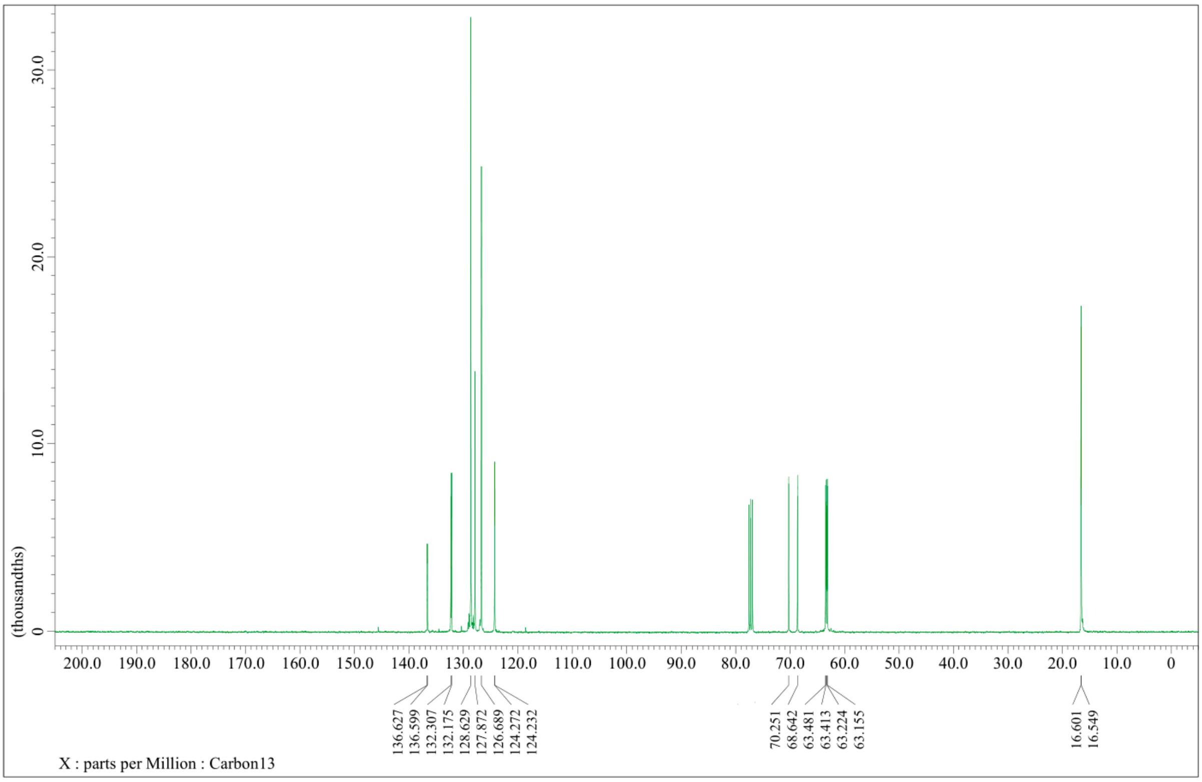

^{31}P NMR spectrum of **3m**

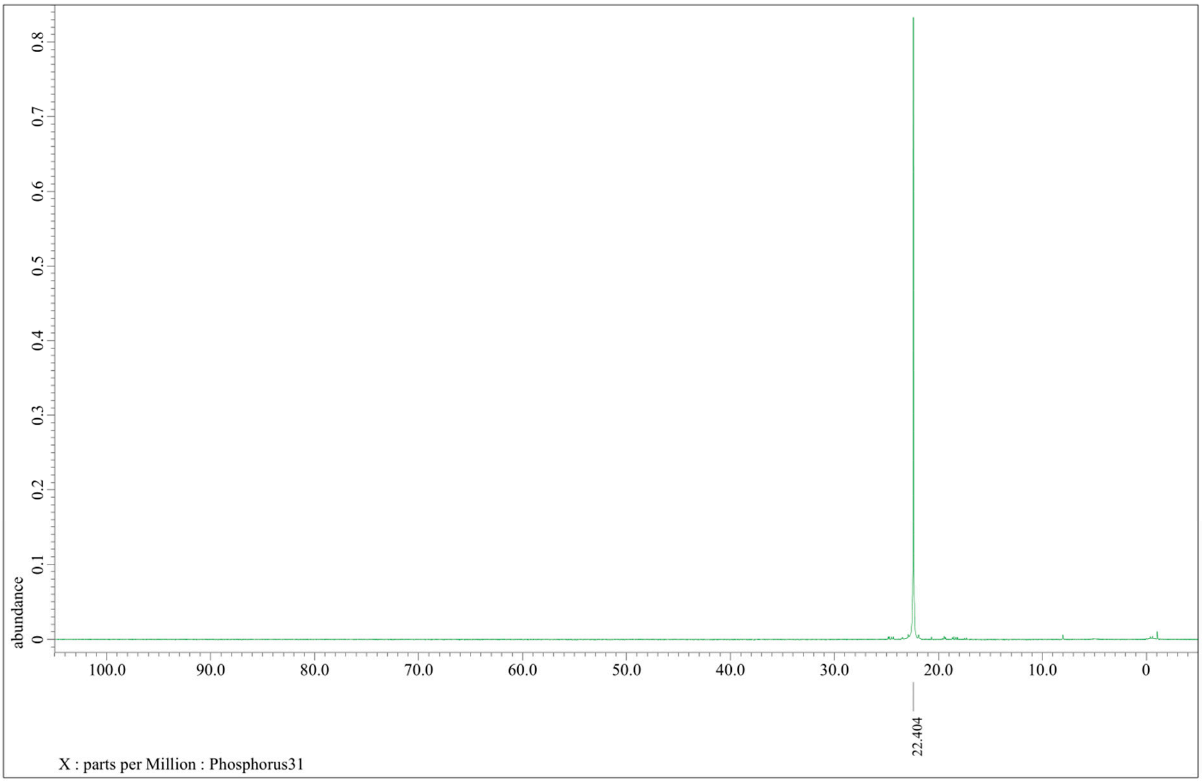

Dimethyl (1-hydroxyphenylmethyl)phosphonate (**5b**)

^{1}H NMR spectrum of **5b**

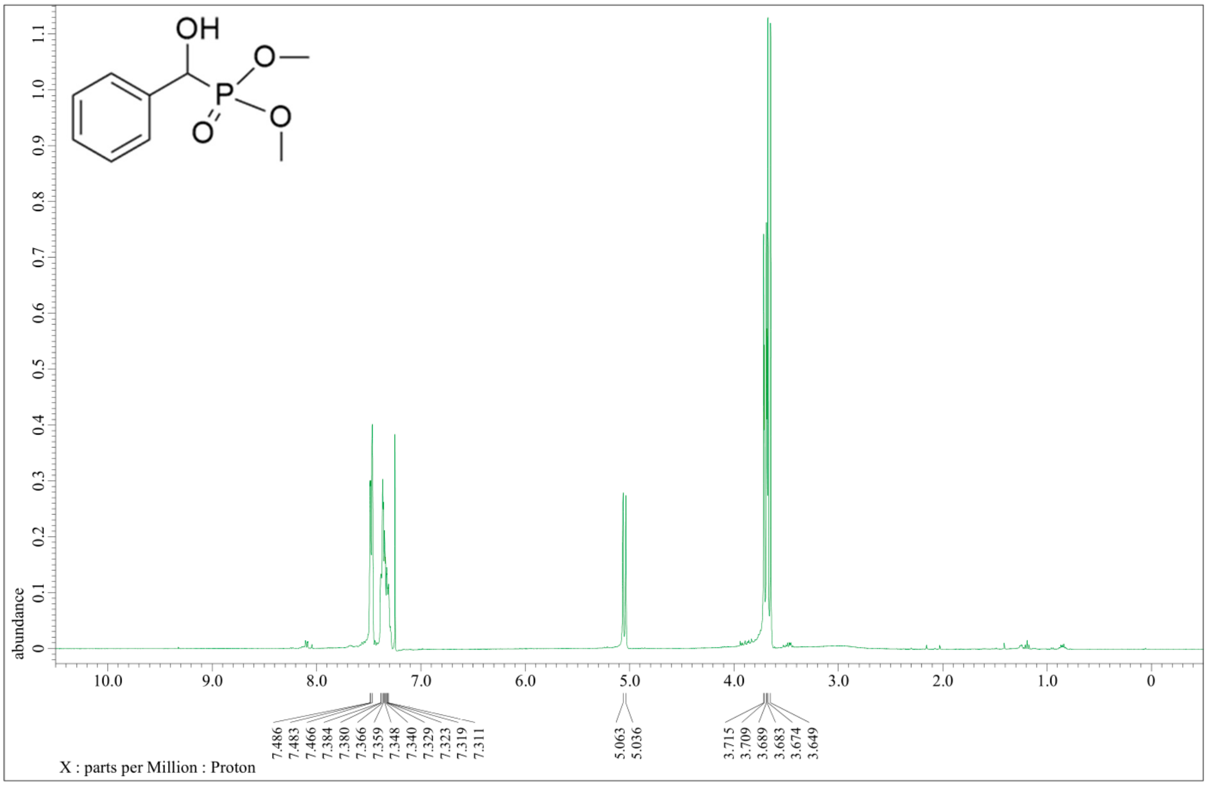

^{13}C NMR spectrum of **5b**

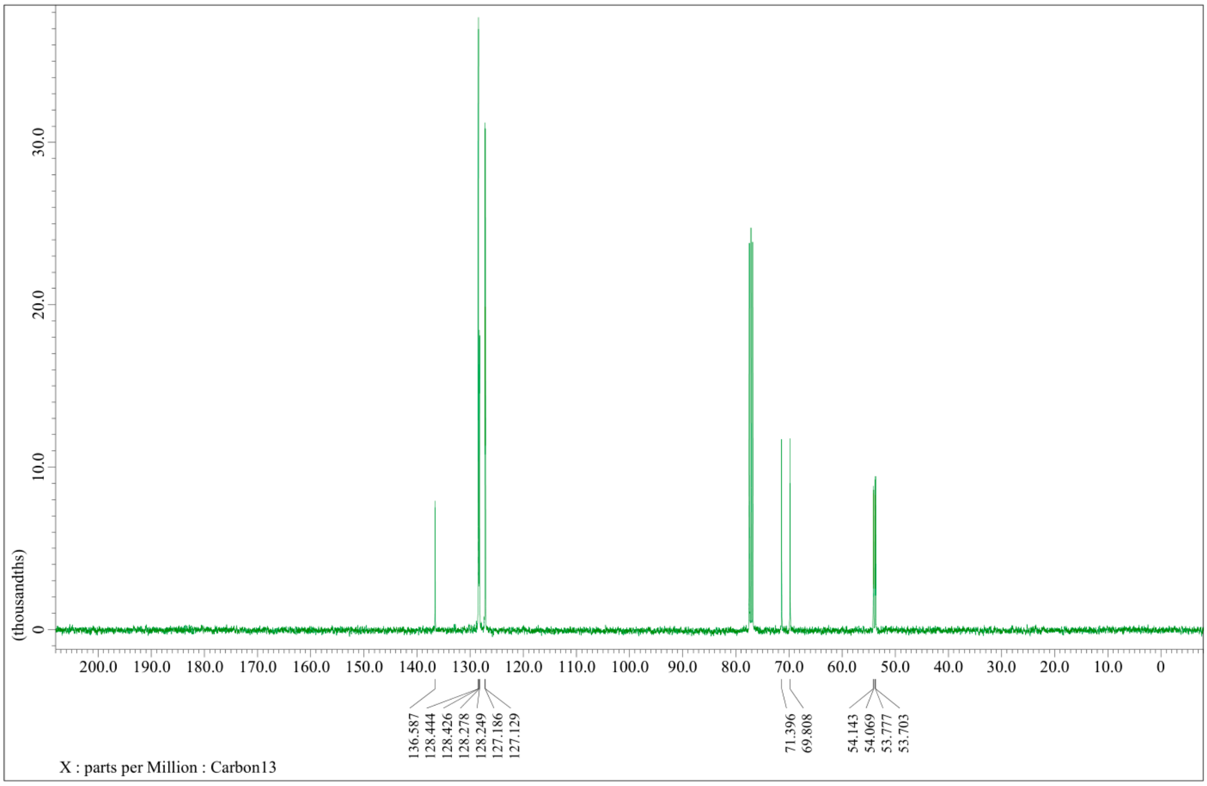

^{31}P NMR spectrum of **5b**

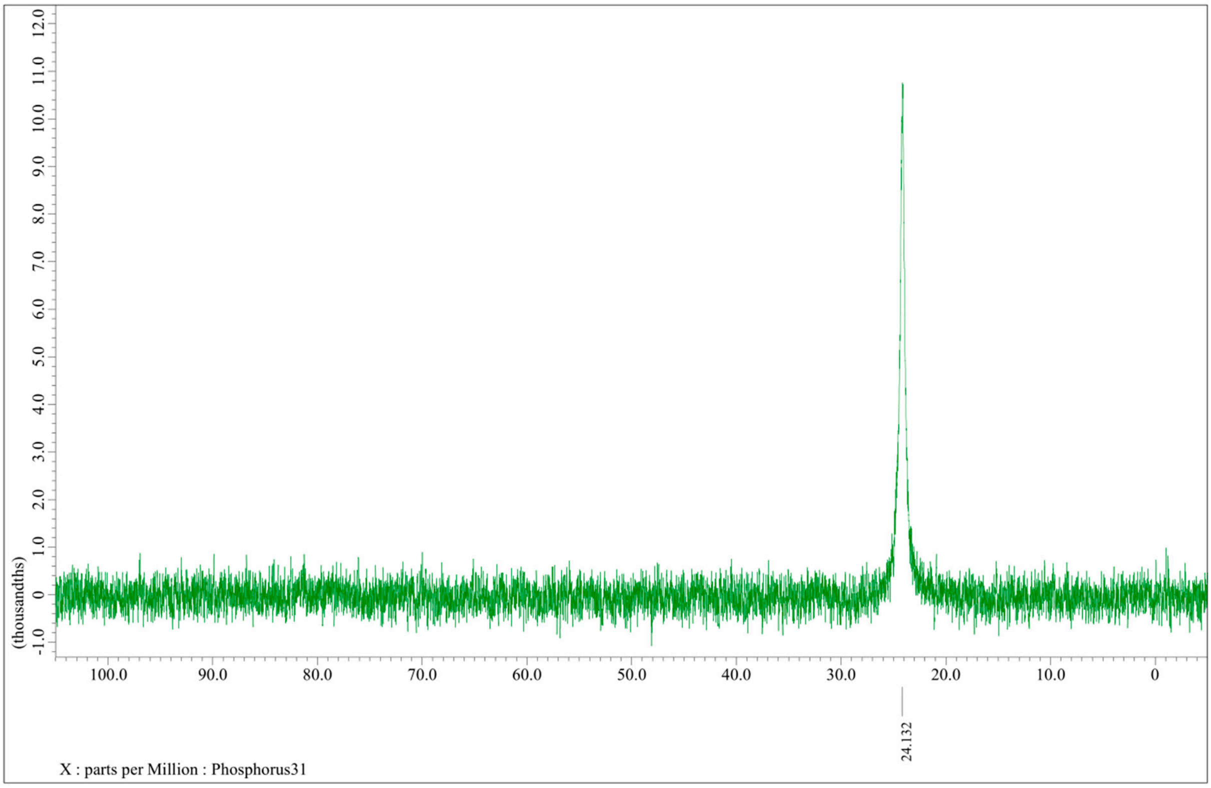

Diisopropyl (1-hydroxyphenylmethyl)phosphonate (5c)

^{1}H NMR spectrum of **5c**

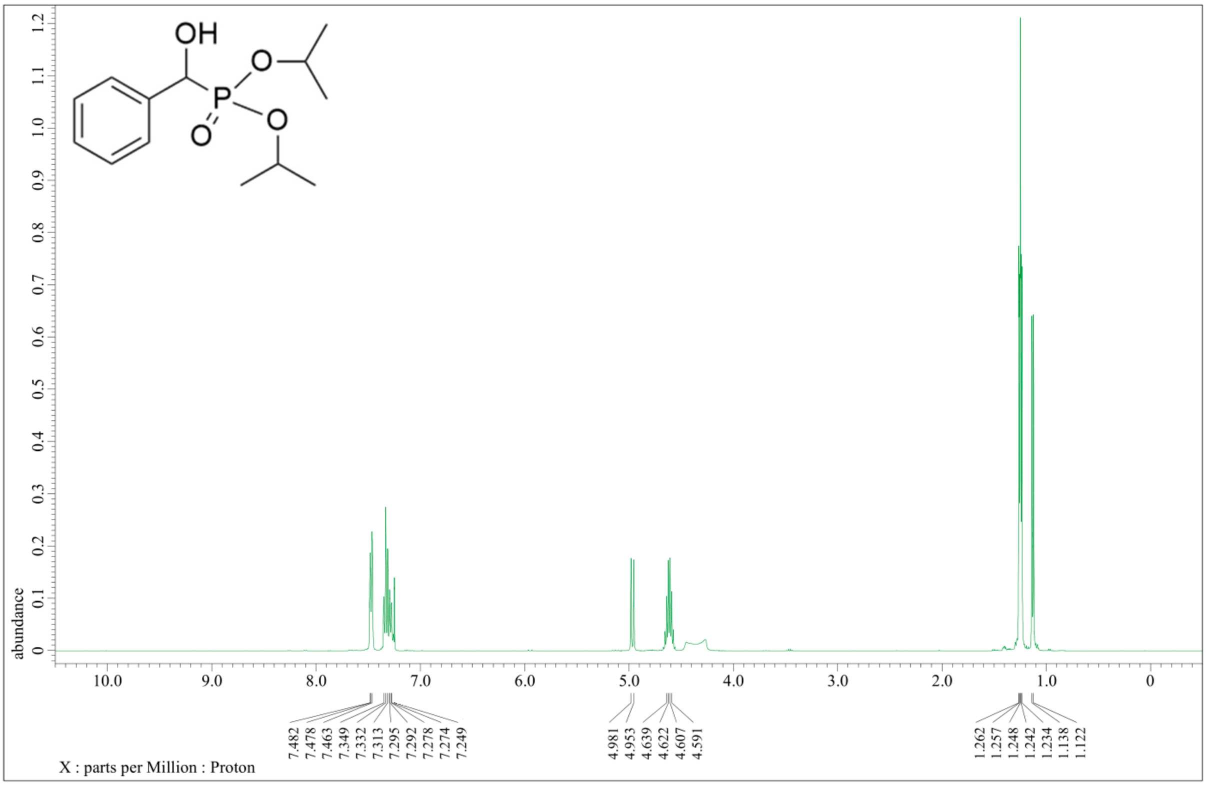

^{13}C NMR spectrum of **5c**

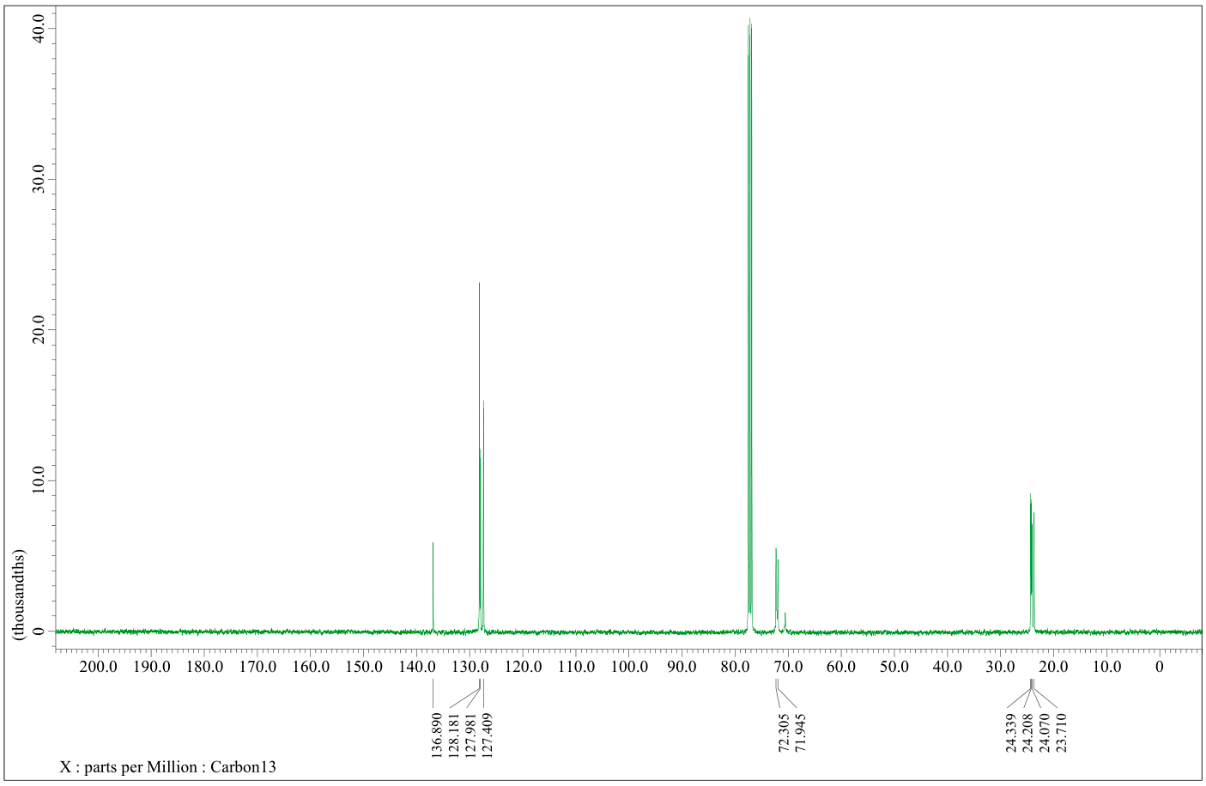

^{31}P NMR spectrum of **5c**

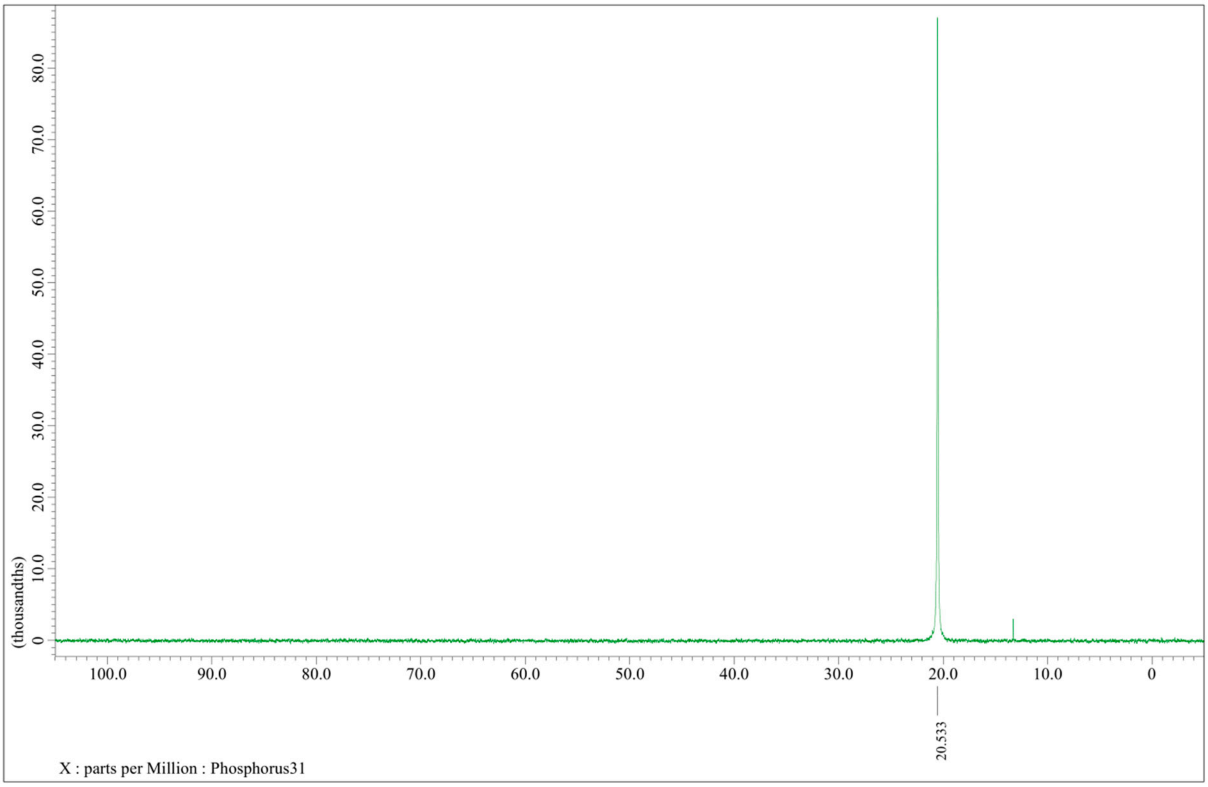

Dibenzyl (1-hydroxyphenylmethyl)phosphonate (5d)

^{1}H NMR spectrum of **5d**

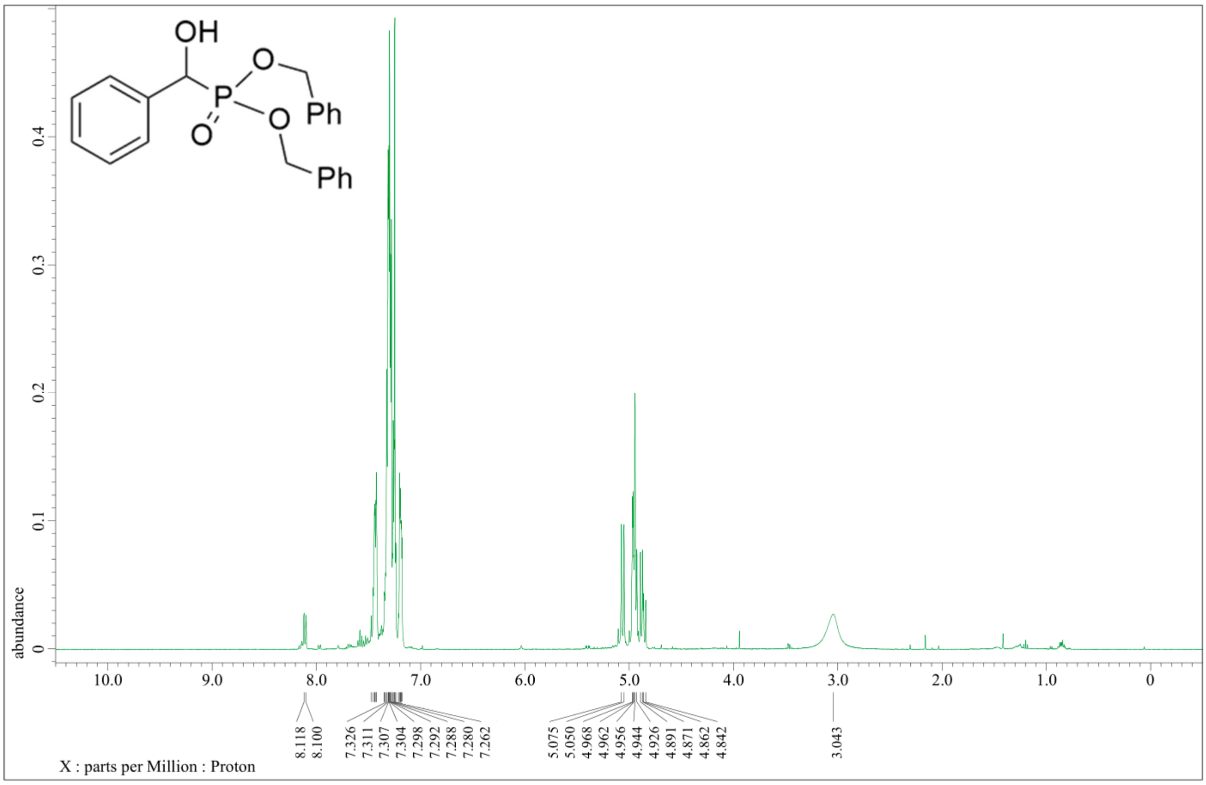

^{13}C NMR spectrum of **5d**

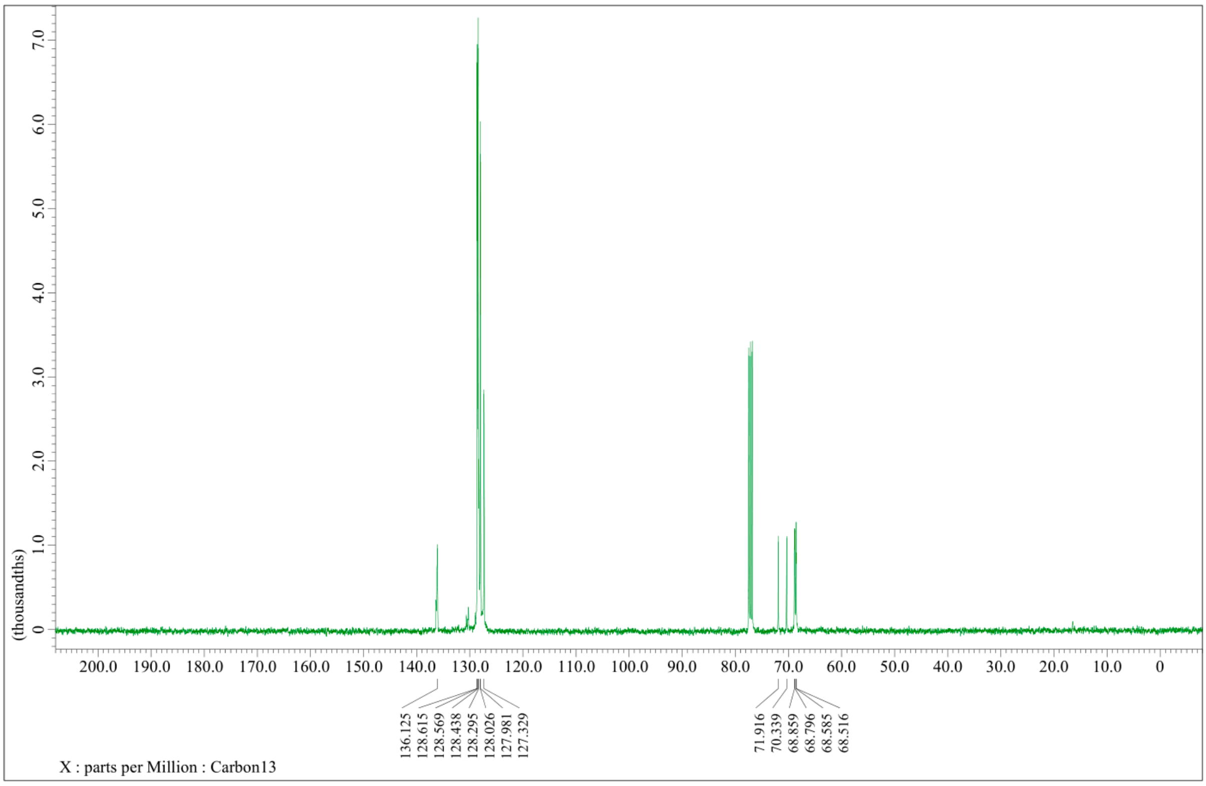

^{31}P NMR spectrum of **5d**

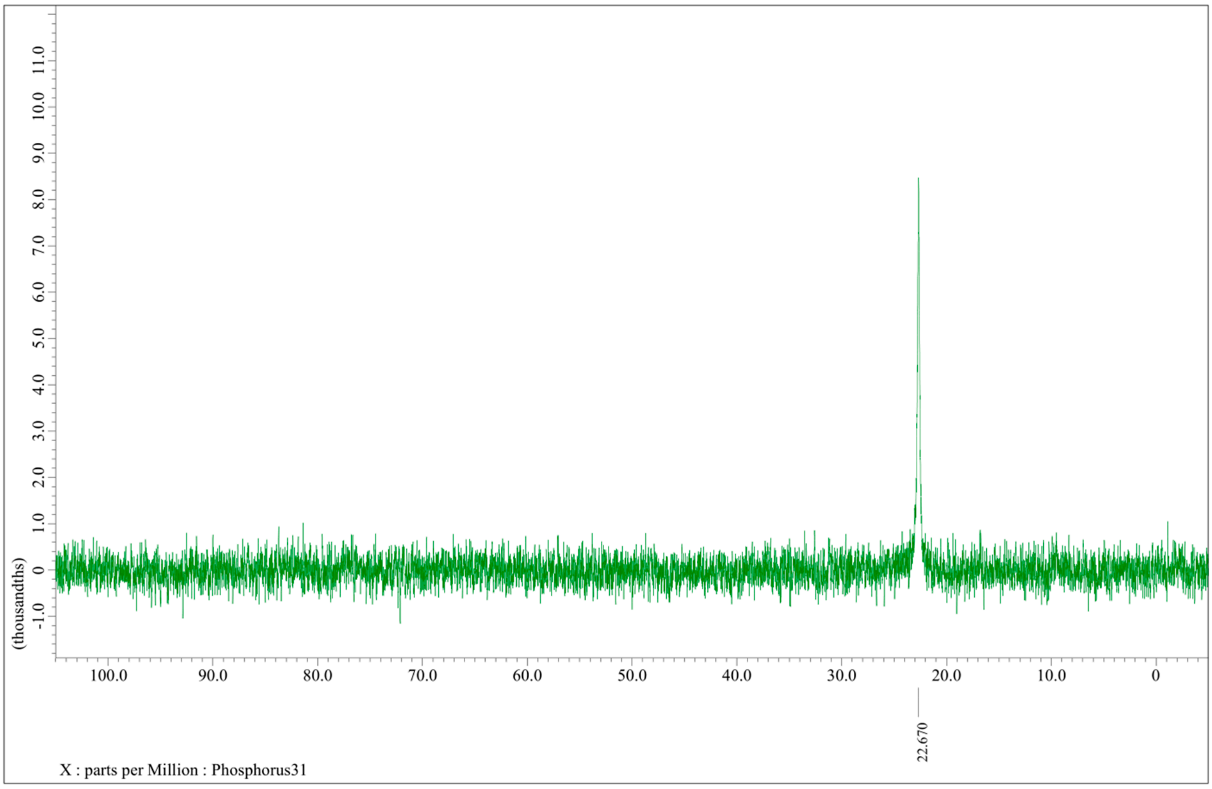

Dimethyl 1-hydroxy-3-methyl-butylphosphonate (5e)

^{1}H NMR spectrum of **5e**

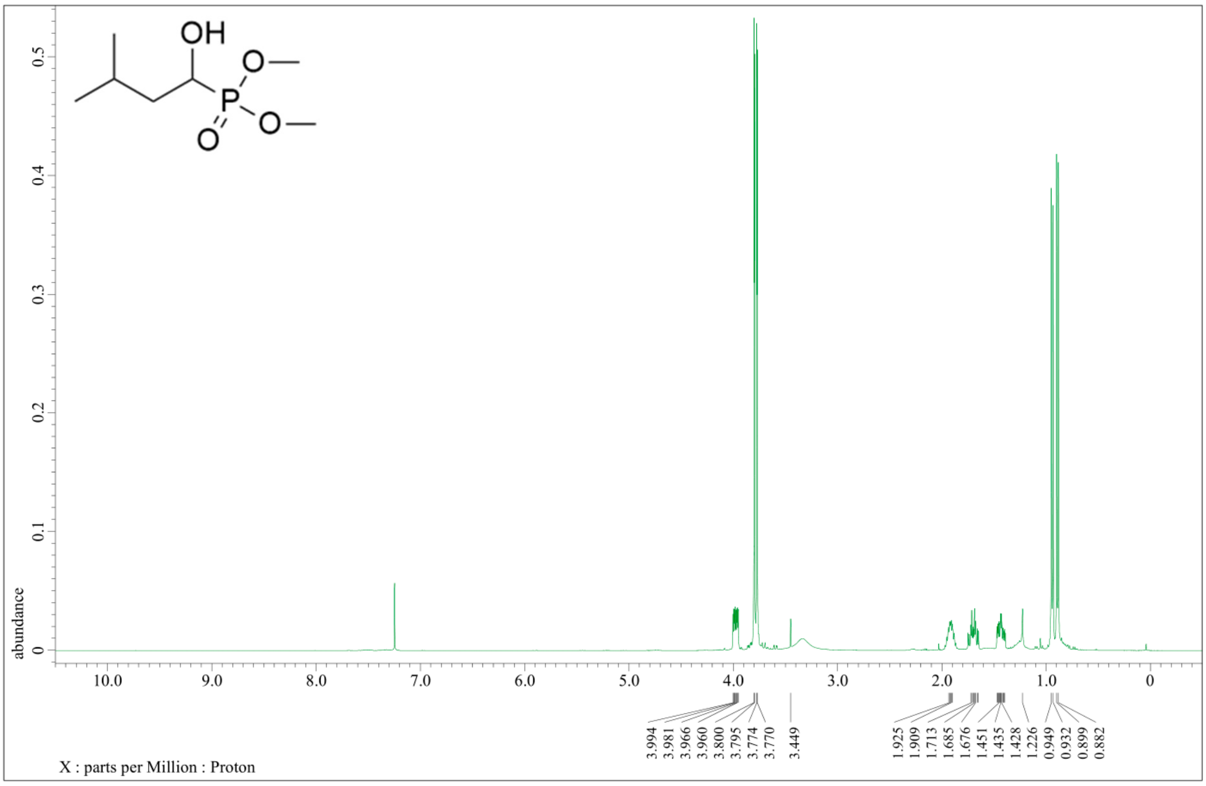

^{13}C NMR spectrum of **5e**

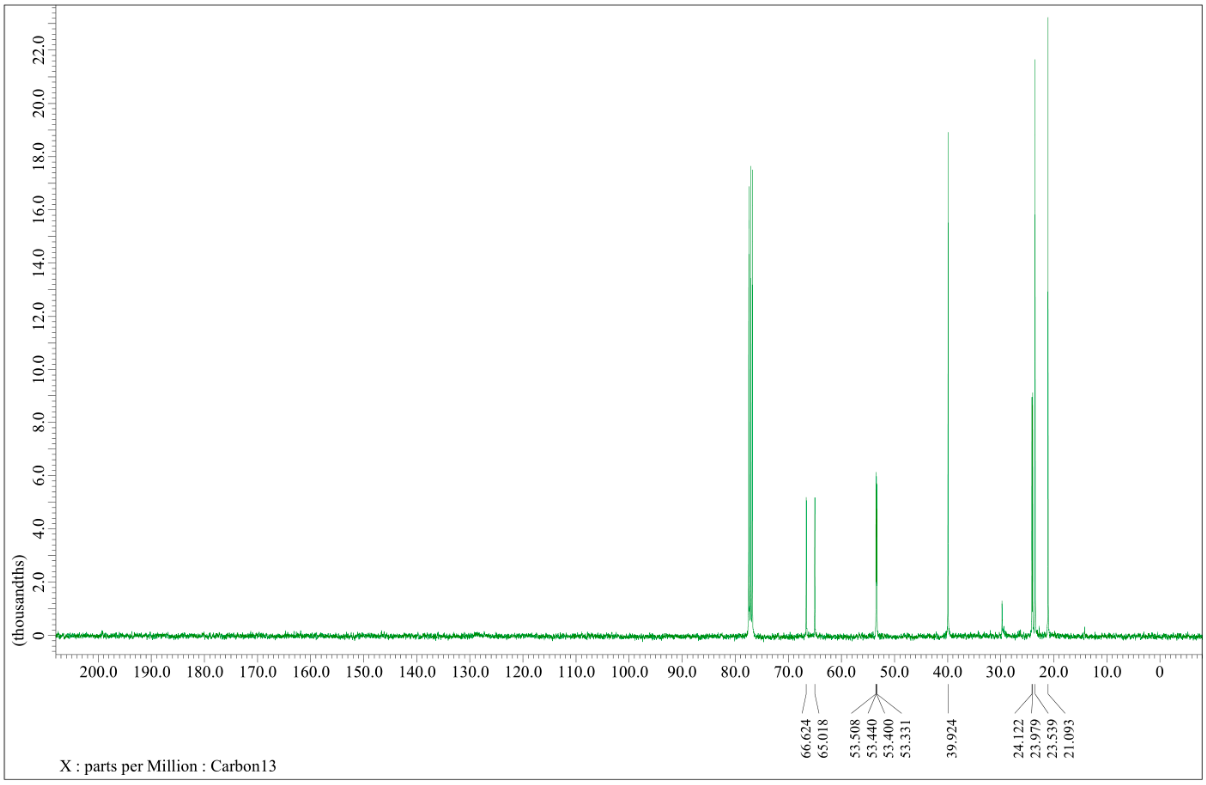

^{31}P NMR spectrum of **5e**

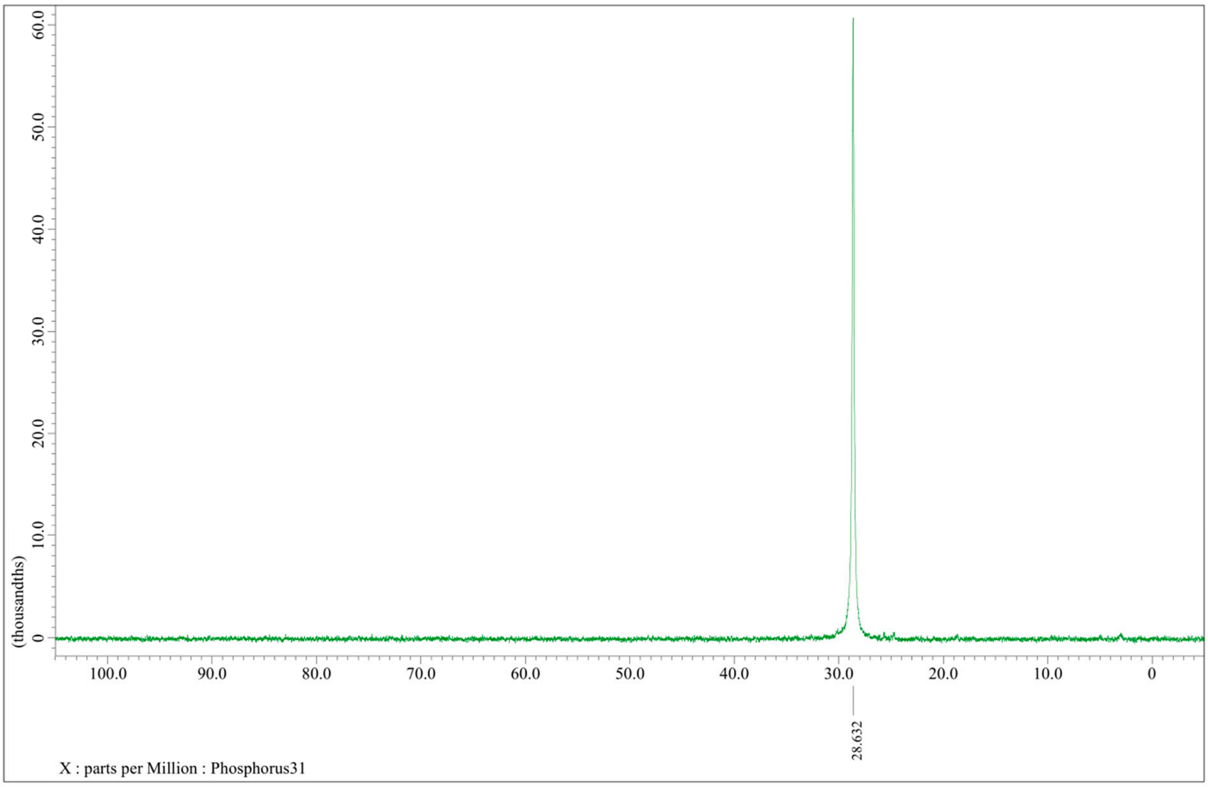

Dibenzyl 1-hydroxy-3-methyl-butylphosphonate (5f)

^{1}H NMR spectrum of **5f**

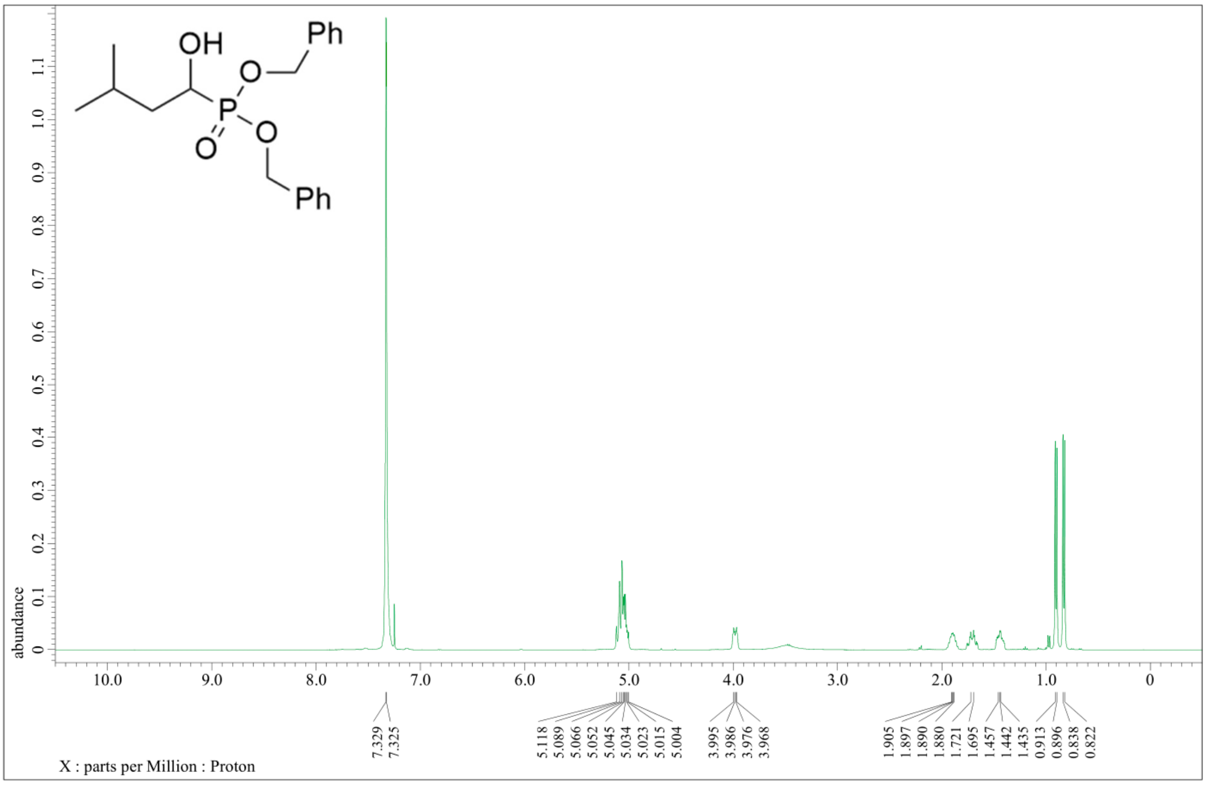

^{13}C NMR spectrum of **5f**

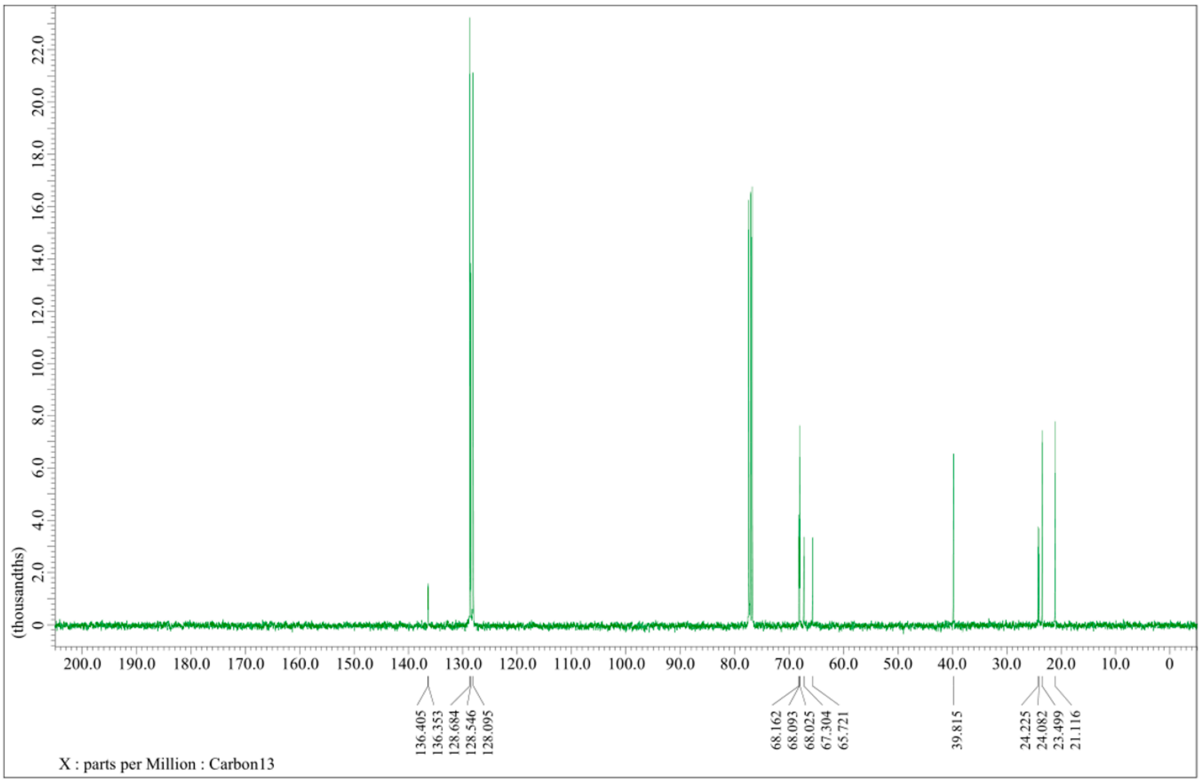

^{31}P NMR spectrum of **5f**

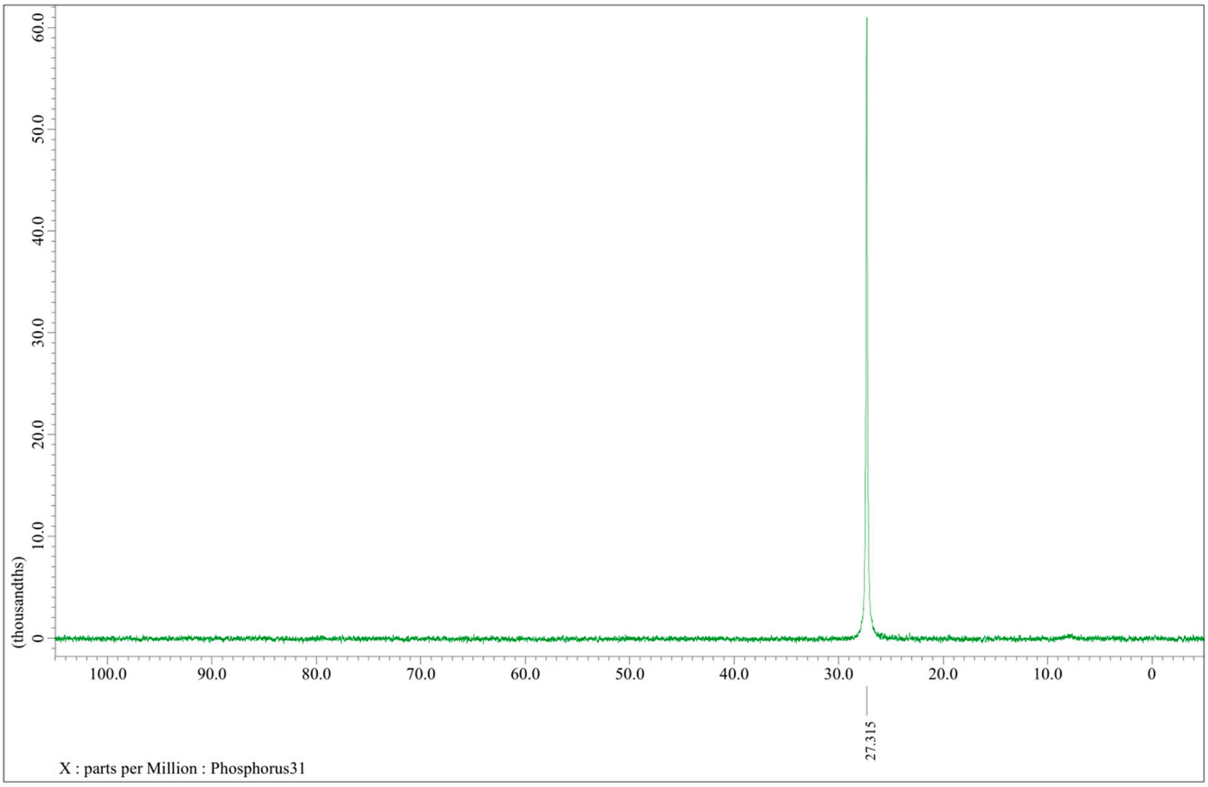

Dimethyl 1-hydroxy(pyridine-3-yl)methylphosphonate **(5g)**

^{1}H NMR spectrum of **5g**

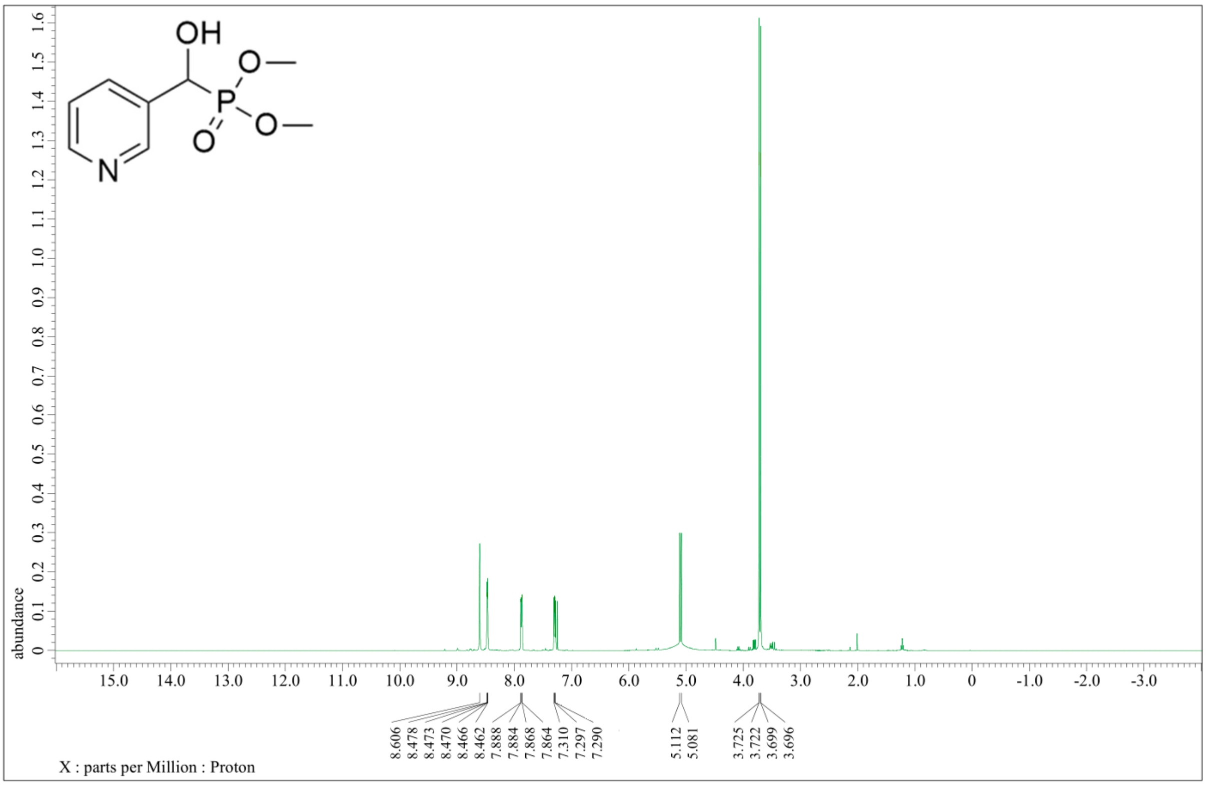

^{13}C NMR spectrum of **5g**

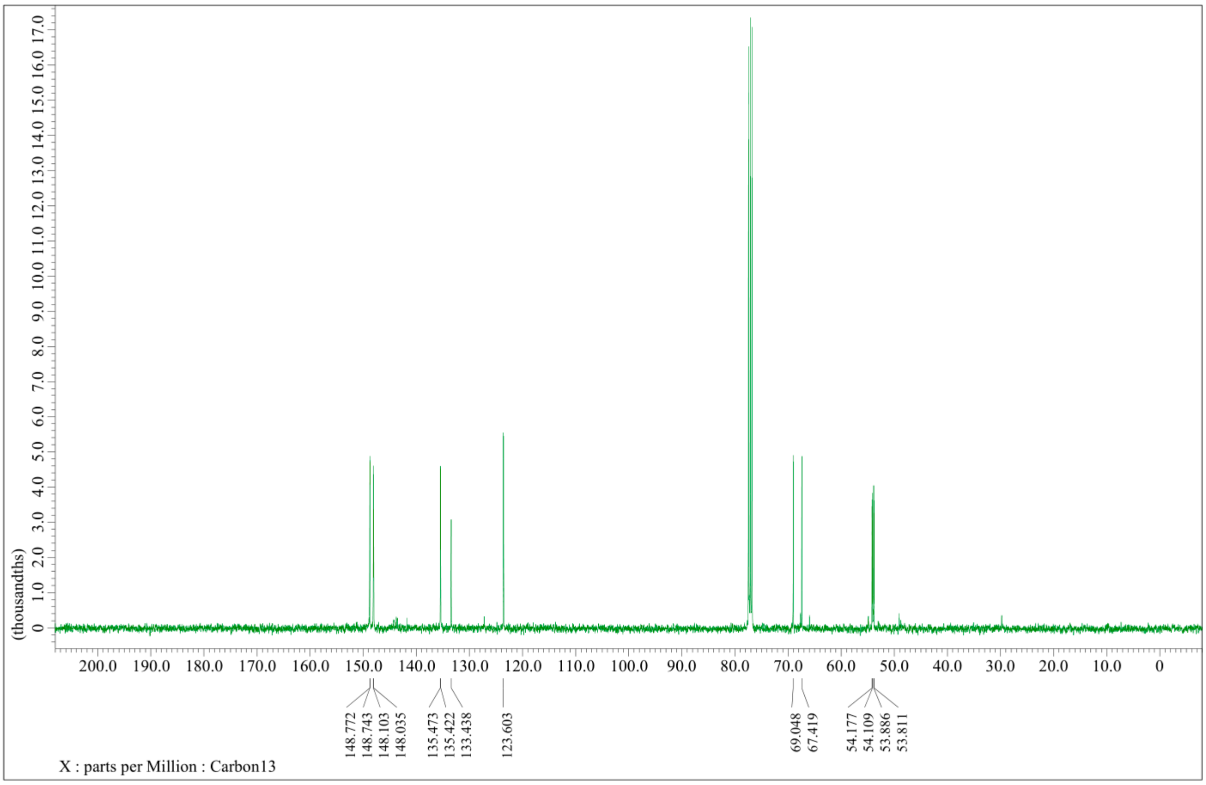

^{31}P NMR spectrum of **5g**

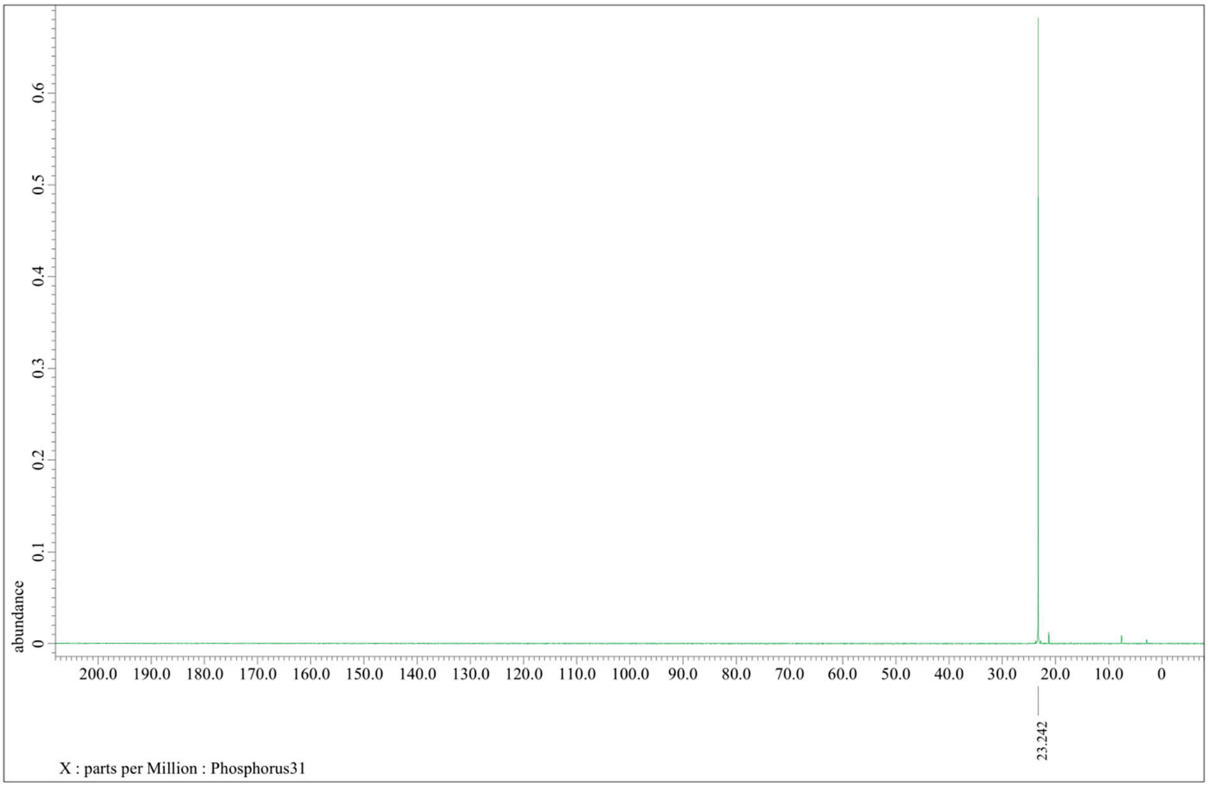

Dibenzyl 1-hydroxy(pyridine-3-yl)methylphosphonate (5h)

^{1}H NMR spectrum of **5h**

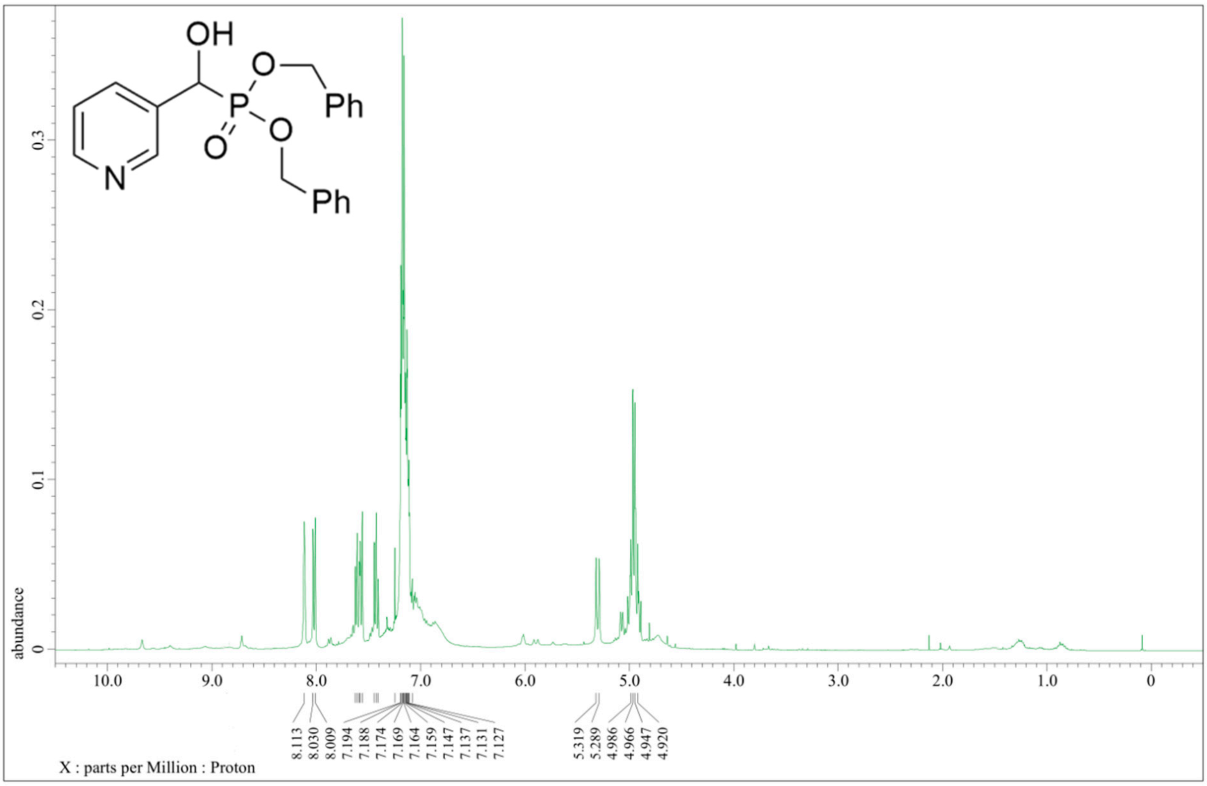

^{13}C NMR spectrum of **5h**

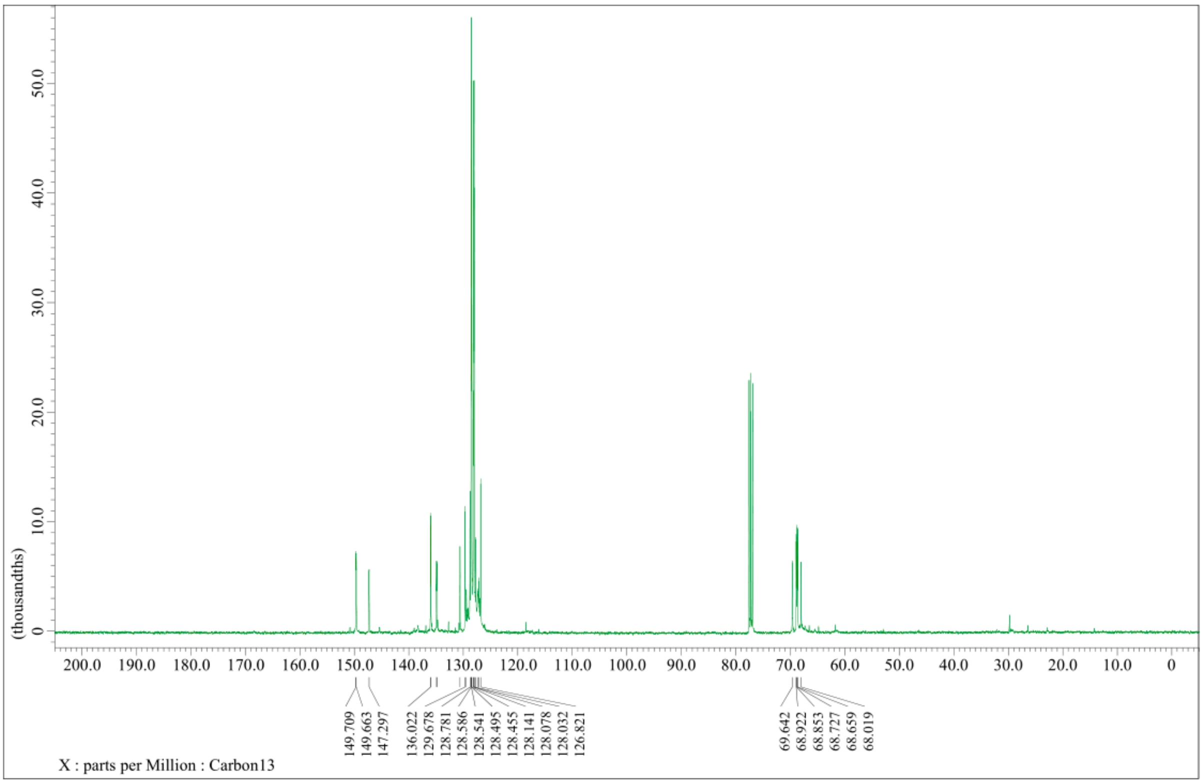

^{31}P NMR spectrum of **5h**

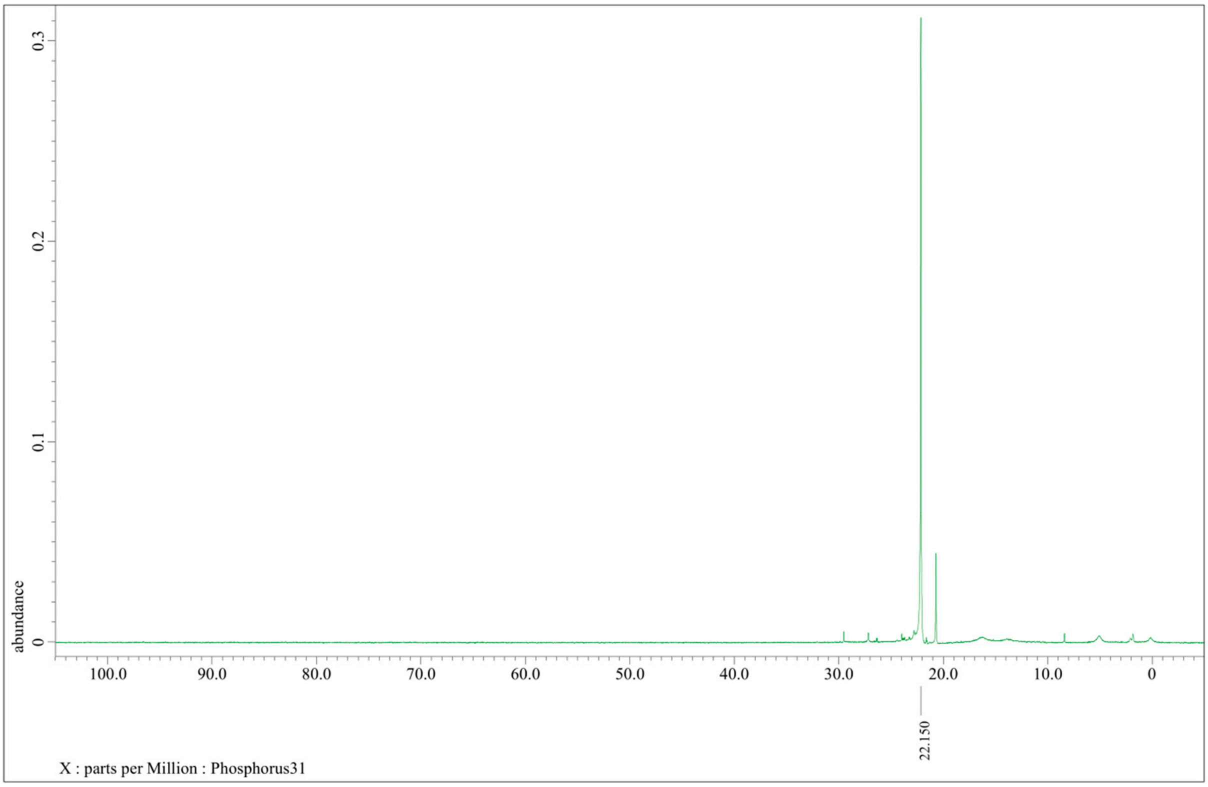

Theoretical calculations

- Phosphite deprotonation – site dependency:

$(Mg_{1.0}Zn_{0.0})O$ - phosphite<br>stabilization energy ($kcal.mol^{-1}$)	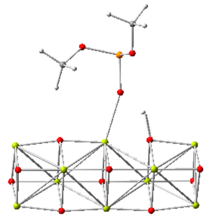<br>46.68	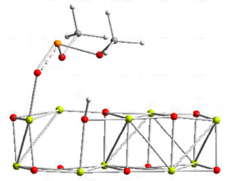<br>65.75	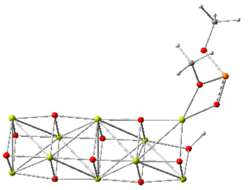<br>81.84
$(Mg_{0.9}Fe_{0.1})O$ - phosphite<br>stabilization energy ($kcal.mol^{-1}$)	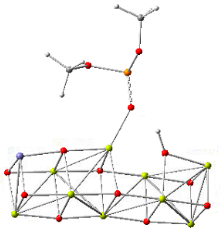<br>54.39	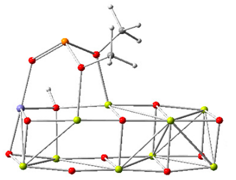<br>58.26	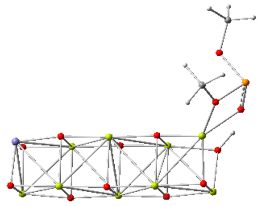<br>81.74
$(Mg_{0.9}Zn_{0.1})O$ - phosphite<br>stabilization energy ($kcal.mol^{-1}$)	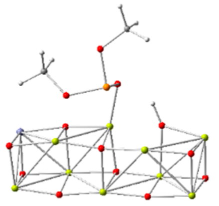<br>46.99	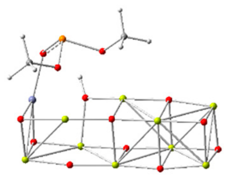<br>64.73	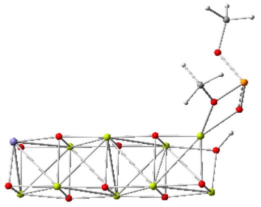<br>81.81

Table of stabilization energies for clusters between oxides and phosphite at different positions. White, grey, red, orange, yellow, light purple and dark purple spheres represent respectively hydrogen, carbon, oxygen, phosphorous, magnesium, iron and zinc atoms.

- C-P bond formation:

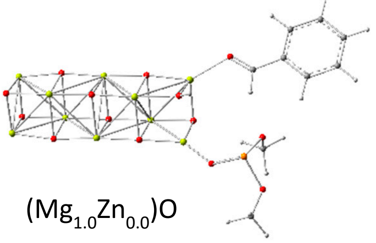

$(Mg_{1.0}Zn_{0.0})O$

$d_{C\text{-}P}$ = 4.31 Å

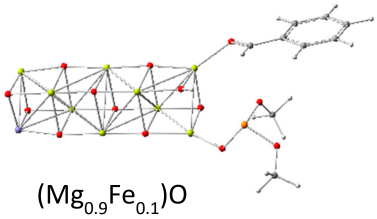

$(Mg_{0.9}Fe_{0.1})O$

$d_{C\text{-}P}$ = 3.62 Å

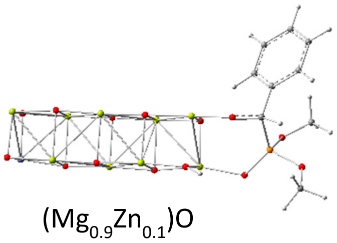

$(Mg_{0.9}Zn_{0.1})O$

$d_{C\text{-}P}$ = 1.93 Å

Geometry optimized conformation when the aldehyde is put in presence of the phosphite-metal oxide complex. The formation of the α-Hydroxyphosphonate is only observed with MgZnO oxide. C-P bond distance is expressed in angstroms. White, grey, red, orange, yellow, light purple and dark purple spheres represent respectively hydrogen, carbon, oxygen, phosphorous, magnesium, iron and zinc atoms.
